# Six Sigma model with implement SAPF to enhance power quality and product quality at induction heat treatment process

**DOI:** 10.1038/s41598-025-03787-x

**Published:** 2025-07-02

**Authors:** Minh Ly Duc, Petr Bilik, Radek Martinek

**Affiliations:** 1 Faculty of Commerce, Van Lang University, Ho Chi Minh City, Vietnam; 2 Department of Cybernetics and Biomedical Engineering, Faculty of Electrical Engineering and Computer Science, VSB–Technical University of Ostrava, 17. listopadu 15, Ostrava, 708 00 Czechia

**Keywords:** MADM, DMAIC, PLS-SEM, Power energy, Manufacturing cost, Electrical and electronic engineering, Mechanical engineering

## Abstract

This study proposes a model to integrate the Multi-Attribute Decision-Making (MADM) method into the Analysis phase of the Six Sigma (DMAIC) method to improve product quality and optimize processing conditions during high-frequency quenching heat treatment. One of the breakthroughs of the study is the combination of Industry 4.0 technology and the implementation of Shunt Active Power Filter (SAPF) to improve power quality, reduce harmonic distortion (THD), ensure product hardness of 58–62 HRC, and thermal permeability of 1.8–2.2 mm according to standards. Previously, many studies only focused on improving the heat treatment process but did not fully integrate MADM, Six Sigma, and Industry 4.0 technology, nor did any study consider the combination of SAPF to control power quality during high-frequency quenching. Another gap is the lack of quantitative assessment of operator satisfaction after improvement using PLS-SEM. The study applied the Six Sigma DMAIC model combined with MADM to analyze and rank factors affecting product quality. In the improvement phase, the Taguchi method was used to optimize processing conditions, minimizing errors in the production process. At the same time, Industry 4.0 technology and RFID systems were integrated to control production conditions in real time, ensuring the accuracy and reliability of the process. Power quality was improved thanks to the implementation of SAPF, helping to control harmonic distortion (THD) below 5% according to the IEEE 519:2022 standard, minimizing the negative impact of voltage on the heat treatment process. In addition, the study also applied PLS-SEM to measure operator satisfaction after implementing the improved system. The research results show that the rate of substandard products has decreased sharply from 90 to 1%, ensuring hardness of 58–62 HRC and thermal permeability of 1.8–2.2 mm. Power quality is better controlled, with the THD value reduced from more than 34% to less than 5%, meeting the IEEE 519:2022 standard. As a result, production costs are optimized, helping to minimize the waste of raw materials and energy. After implementing the improved system, operators’ satisfaction levels have also increased significantly, reflected in the PLS-SEM measurement indicators. More importantly, this research model is not only effectively applied in the precision engineering industry but also has the potential to be expanded to many other industries, especially small and medium-sized manufacturing enterprises, helping them to increase productivity and improve product quality in the context of Industry 4.0.

## Introduction

### Motivation and background

The manufacturing and processing industry applying science and technology 4.0 to improve production processes is a modern trend^1^. Many decades ago, Industry 3.0 was developed and put into operation in production processes. In recent decades, along with the development of Internet of Things (IoT) technology, industry 3.0 has turned into Industry 4.0^2^. The production process applies 4.0 technology to help operate more flexibly and bring high efficiency to production^3^. The rapid development of IoT devices has helped increase the operational flexibility of Industry 4.0 in the manufacturing environment and helped save production costs^4^. The research question for the production process is whether implementing Industry 4.0 into the production process will help improve operational efficiency and enhance competitiveness in production costs^5^. The high-frequency quenching heat treatment process is a unique and complex manufacturing environment with uncertain manufacturing conditions. The second research question in this study is the optimal operation of production conditions in the high-stage quenching process, which includes conditions such as the cooling water concentration gradually equaling MG815 and the running current intensity. In the coil, the diameter of the product requiring high-stage quenching heat treatment, the chemical element composition of the SUJ2 raw material, the moving speed of the coil, and the surface gloss of the product help improve production efficiency, save energy, and is easy to operate and control product quality^6^. Achieving the operating conditions of the high-stage thermal quenching process depends entirely on the condition of the current intensity supplied to the coil. Production costs incurred in the high-stage thermal quenching process arise when the current electricity intensity does not meet the requirements of the IEEE 519:2022 standard and requires a value level to provide amperage for each corresponding product line. The research question is whether power source quality control activities help improve product quality by ensuring product surface hardness from 58 HRC to 62 HRC and the depth of the hardness layer within the standard of 1.8 mm to 2.2 mm^7^. Improving power quality by implementing and installing active filters is a good method to consider^8^.

Low-cost production is developed thanks to applying 4.0 techniques and converting semi-automatic structures into automatic ones^9^. The production conditions of the high-frequency quenching production process are optimized to help save production costs, improve product quality, and improve the company’s production and business efficiency. Technology 4.0 was developed according to the criteria of simple operation, meeting the operator’s needs^10^. Production costs in the high-stage quenching process are saved. Specifically, MG815 material reduces the rate of evaporation and all current intensity and voltage intensity supplied to the coil, reducing the defect rate. The digital numerical control (DNC) method helps call high-rise heat generator programs using an RFID system linked to the company’s production management system, helping to prevent incorrect program calls, helping to improve safety operations in the process operation of the high-rise thermal quenching process and improve the satisfaction of machine operators and customers regarding product quality perception^11^. The criteria for developing a production process for decision-makers in a manufacturing company are to develop a production process that is simple to operate, has low production costs, and has flexible operating conditions for each process. Active and safe. Production condition and product quality control data are stored over time, making it possible for different processes to access one source and view results easily. Production data linked between production processes helps improve efficiency in data analysis activities to help decision-makers in the company guide business development strategies^12–14^. An industry 4.0 system designed and deployed at low cost is essential to help small and medium-sized businesses have the opportunity to grow^15^. Although the low-cost Industry 4.0 system brings economic efficiency when investing for small and medium-sized companies, poor evaluation and monitoring activities of a management department negatively affect the efficiency of operational efficiency of the Industry 4.0 system^16,17^.

Small and medium-sized companies’ limitations are that their resources are not qualified or aware of Industry 4.0 technology, and their investment capital resources are still weak, so they do not have enough requirements and ability to deploy Industry 4.0 systems^18^. Another limitation is that decision-makers of small and medium-sized companies are not determined to apply Industry 4.0 technology to improve production processes from Industry 3.0 technology to Industry 4.0 technology^19,20^. The first research question is whether implementing Industry 4.0 technology to improve production processes brings about improvement in product quality, production process efficiency, and business efficiency in production and business necessity.

All machines in the cashew production process use power to operate, and the quality of the power source positively impacts the machine’s efficiency. The harmonic signal is the agent that causes power quality deterioration in the power supply system for processing machines. Devices using Industry 4.0 technology are the main cause of generating harmonic signals, and devices using high-frequency switching in automatic control include switches, LED lights, tablet screens, and monitors. The second research question is whether harmonic mitigation designed by hardware devices, such as active power filters, is necessary, and the value brought by controlling the THD value in the power source within the IEEE 519:2022 standard is small. More than 5% is essential to help the processing machine ensure accuracy during operation, especially for high-temperature quenching machines.

Manufacturers participating in this project are faced with improving product quality, reducing defect rates, and ensuring stability in mechanical manufacturing processes. The main challenge lies in the fact that factories produce defective products, which affects reputation, costs, and production efficiency. To solve this problem, manufacturers need to perform root cause analysis using tools such as Ishikawa diagrams or Pareto analysis to identify and eliminate the main causes of defects. Next, applying the DMAIC model in Six Sigma and the Taguchi method will help optimize operating parameters, from current intensity and heating time to coil spacing. At the same time, improving power quality by installing adaptive power filters (SAPF) to reduce harmonics and ensure power supplies meet IEEE 519:2022 standards is an important solution. In addition, implementing a real-time monitoring system based on Industry 4.0 technology will help monitor production conditions and product quality. At the same time, employee training programs will improve awareness and skills in quality control. To prevent the recurrence of defects, manufacturers need to store and analyze production data to predict and prevent similar errors in the future, combined with periodic inspection and maintenance of machinery. These solutions not only help reduce defect rates and improve production efficiency but also increase customer satisfaction and improve business reputation, ensuring competitiveness and sustainable development in the long term.

The research question focuses on solving the problem of improving product quality in the manufacturing process by identifying and applying the optimal improvement method and finding the most suitable operating conditions for this method to be effective. The research aims to identify important quality aspects of mechanical products, thereby making quality improvement decisions based on data analysis and modern optimization tools. By applying methods such as DMAIC in Six Sigma, MADM to prioritize issues that need improvement, and the Taguchi method to optimize technical parameters, the research not only focuses on improving the manufacturing process but also ensures that product quality meets strict standards. In addition, modern technologies such as real-time monitoring systems based on Industry 4.0 technology and adaptive power filters (SAPF) are also deployed to control important factors such as power quality and operating conditions. The expected result is not only to improve the quality of mechanical products but also to increase production efficiency, reduce defect rates, and establish a sustainable process, bringing long-term benefits to the manufacturing company.

Eliminating non-value-added activities is a core goal to increase business profits. Managers are always looking for ways to optimize production by applying Industry 4.0 techniques, which help automate processes, shorten processing times, eliminate waste, and improve productivity. This technology not only supports optimizing production activities but also allows for data storage and analysis during processing, flexibly meeting diverse customer requirements, thereby improving management efficiency and business competitiveness.

Power quality plays a critical role in industrial manufacturing, especially in induction heat treatment, where high-frequency currents are required to heat metallic materials in a uniform and controlled manner. However, in modern manufacturing systems, power quality issues such as total harmonic distortion (THDi), voltage fluctuations, low power factor, and voltage imbalance can significantly impact heat treatment performance and product quality. These deviations result in uneven temperature distribution in the material, reducing hardness uniformity, increasing defective product rates, and wasting energy. One of the most common problems in induction heat treatment is current fluctuations, which change the magnetic field strength around the treated material, resulting in unstable temperatures. When THDi exceeds 12%, high-order harmonics can degrade the performance of induction coils, causing energy imbalance and leading to over- or under-heating, directly affecting the material’s hardening depth. This increases the rate of substandard products and reduces the equipment life due to thermal overload, causing production interruptions and increasing maintenance costs. To solve these problems, this study proposes a model combining Six Sigma with Active Power Filter (SAPF) to optimize power quality and strictly control parameters during induction heating treatment. SAPF plays an important role in reducing harmonics, improving power factor, and stabilizing current. At the same time, Six Sigma helps analyze, measure, and optimize the manufacturing process to reduce the rate of defective products. By combining these two methods, the study aims to improve the accuracy of the heat treatment process, significantly reduce the hardness defect rate, and improve production efficiency. This study not only helps improve product quality but also contributes to reducing energy consumption and improving the stability of the production system, helping enterprises better comply with power quality standards such as IEEE 519:2022. In the next section, the study will delve into the methods of analyzing and evaluating the effectiveness of the SAPF + Six Sigma model in controlling power quality and optimizing the induction heat treatment process, thereby proposing strategies to improve production quality in modern industrial environments.

The research is carried out according to the following specific objectives:

- Build a general model to calculate production costs in the high-rise quenching heat treatment process and the overall production cost.
- Build the MCDM method model into the defined phase of the Six Sigma method to select problem points to improve the high-rise thermal quenching production process.
- Design and deploy an active power filter device into the power supply for the high-rise thermal quenching machine to improve the quality of the power source and ensure the cos(φ) coefficient is greater than 0.9, and the THD parameter value is guaranteed according to the IEEE 519:2022 standard, which is less than 5%.
- Evaluation of low production costs in high-rise quenching heat treatment processes in a competitive environment.
- Build and deploy an Industry 4.0 technology model to measure and control machining conditions and product quality at the high-rise heat treatment process at a low cost to improve production and business efficiency in small and medium companies.
- Measuring system user satisfaction after improvement in the heat treatment process confirms the effectiveness and inadequacies in the operation of the continuous improvement system to help improve the efficiency of continuous improvement activities using the PLS-SEM method.

### Content, contribution, and organization of this study

This study suggests using the Multi-Attribute Decision Making (MADM) approach to analyze and choose which items require modification to increase stability throughout the Six Sigma method’s analysis phase. The high-rise thermal quenching process’s processing conditions. Proposal to optimize production conditions throughout the high-temperature quenching process and apply the Taguchi method in the Six Sigma improvement phase to meet customer requirements and ensure stable product quality after high-temperature quenching. The depth of penetration is between 1.6 and 2.2 mm. To comply with IEEE 519:2022, suggest a practical power quality measurement at the high-frequency quencher’s source and watch for Total Harmonic Distortion (THD) values under 5%. The Taguchi approach is used to optimize the current intensity (A) to help regulate the energy source utilized by the high-stage quenching machine. In the improvement phase of the Six Sigma method, it is proposed to use a Digital Numerical Control (DNC) system based on the Industry 4.0 technology platform to scan data from RFID objects, retrieve data, and link data between production processes and product quality control results. Implementing system maintenance schedules in the online measurement system is also proposed. According to the technical readiness measurement variables, a PLS-SEM approach is suggested to be deployed during the Control phase of the Six Sigma method to gauge user satisfaction with the upgraded system during the high-rise thermal quenching process. Approaches, information engineering measurement components, and evaluating the utility and usefulness of enhanced systems. In line with the principles of continuous improvement, the author expects that this research article will offer a sample research model in a continuous improvement environment as well as a research model that scales and impacts activities related to continuous improvement in manufacturing businesses. In various industrial firms, the Six Sigma method helps increase productivity and business efficiency while improving the quality of the products and production processes.

In the context of the Industrial Revolution 4.0, which is strongly changing the manufacturing industry, applying advanced technologies to the processing process is an inevitable trend to improve efficiency, optimize costs, and enhance competitiveness. However, the high-frequency quenching process is one of the complex production stages, affected by many factors such as current intensity, chemical composition of the material, coolant concentration, and speed of the induction coil, making it difficult to control product quality. In addition, harmonic distortion (THD) in the electrical system has a negative impact on the hardness and heat permeability of the product, leading to surface defects, deformation, or damage during the processing process. Controlling power quality is an important challenge because unstable power sources can reduce machine performance, waste energy, and increase production costs. On the other hand, small and medium-sized manufacturing enterprises often have difficulty investing in advanced quality monitoring and control systems, making it more challenging to ensure that products meet standards (hardness 58–62 HRC, thermal permeability 1.8–2.2 mm). Therefore, this study aims to integrate 4.0 technology into the production process, helping to automate real-time quality control and propose solutions to optimize processing conditions to reduce product defects, save energy, and improve operating efficiency. In addition, evaluating the satisfaction of operating staff is also an important factor in measuring the effectiveness of the improved system, thereby making appropriate adjustments to improve work efficiency and process stability.

Power quality is an important factor affecting the performance and reliability of industrial manufacturing systems, especially in induction heat treatment. This process requires a high-frequency current to heat metal materials precisely. However, problems such as high harmonic distortion (THDi), voltage fluctuations, low power factor, and voltage imbalance can degrade heat treatment efficiency, leading to uneven temperature distribution, affecting the hardening depth, and increasing the defective rate of products. In many industries, SAPF (Active Power Filter) has been widely used to improve power quality in nonlinear load systems such as variable frequency drives, smart grids, and industrial power systems, but there have been few studies on applying SAPF in induction heat treatment processes. This study’s novelty lies in applying SAPF to control power quality in an induction heating system, which has not been widely explored in previous studies. Unlike traditional SAPF applications that focus on harmonic reduction for induction motors, electronic converters, or microgrid systems, this study focuses on the impact of SAPF on the induced current in the heating coil, a factor that determines temperature stability during material heating. The biggest difference from previous applications is that SAPF is used not only to reduce harmonic distortion (THD) and improve power factor but also to maintain a stable current during short heating cycles, ensuring uniform hardness and optimizing the hardening depth in the metal material. Furthermore, instead of focusing solely on reducing power disturbances in the overall power grid system, this study uses SAPF as part of a manufacturing quality control model, combined with the Six Sigma (DMAIC) method, to measure and optimize the process. This method improves power quality, reduces the defective product rate from 90 to 1%, and improves heat treatment efficiency by strictly controlling current fluctuations, sintering temperature, and hardening permeability. The SAPF + Six Sigma model proposed in this study can be considered a breakthrough in the field of induction heat treatment process optimization, which helps improve product quality, reduce energy consumption, and improve system stability. Compared with previous studies, this study not only considers SAPF as a power electronics solution but also integrates it into the manufacturing quality control system, which helps to improve performance in an industrial field with high requirements for accuracy and stability.

This study chooses fuzzy sets instead of rough sets or extended forms such as IVIFS and q-ROF because fuzzy sets provide better uncertain data handling capabilities, simplify calculations, are easy to deploy in industrial environments, and are suitable for small and medium-sized manufacturing enterprises. This is the optimal choice to help improve production efficiency, minimize product defects, and improve quality in the context of Industry 4.0.

The research paper is organized according to the following structure: Section II: Presenting the content of Raw material and Methodology. Section III: Detailed presentation of the results of the study. Section IV: Show the content of the discussion, and Section V: Present in detail the conclusion of the research paper and future research directions.

### Raw material and methodology

#### Types of manufacturing cost

In production and business, knowing the range of production costs for any product line is essential^21^. Production cost model during raw material induction heat treatment of the precision mechanical product line’s Suj2^22^. This study model explains two production cost computations: the first precisely characterizes the Suj2 metal product line’s production costs, and the second carries out a particular breakdown of the various cost kinds in the manufacturing process using various cost components. The author of this study focuses on calculating production costs in an induction heat treatment process and estimates the total cost associated with business efficiency in manufacturing a mechanical product line using Suj2 material. These costs may vary depending on the circumstances in each process for the particular production process. Examples include selling expenses, infrastructure management costs, and other utility-related expenses related to the high-rise heat curing process. The goal is to develop a model to determine the associated production cost for the induction heat treatment process. To achieve the objective of increasing production efficiency and the company’s business, however, indirect costs have a substantial impact on the total cost of production and must be carefully considered when analyzing both direct and indirect costs in production.

The production cost model for the induction heat treatment process includes expenditures for machines, materials, debinding, sintering, labor, consumables, and other expenditures.

Formula 1 determines the overall manufacturing cost during the production process.

1 $$\:{C}_{pc}={C}_{ma}+{C}_{m}+{C}_{de}+{C}_{si}+{C}_{la}+{C}_{co}+{C}_{ot}\:\:\:\:\:\:\:\:\:\:\:\:\:\:\:\:\:\:\:\:\:\:\:\:\:\:\:\:\:\:\:\:\:\:\:\:\:\:\:\:\:\:\:\:\:\:\:\:\:\:\:\:\:\:\:\:\:\:\:\:\:\:\:$$

Where, $\:{C}_{pc}$: Production cost, $\:{C}_{ma}$: Machine cost, $\:{C}_{m}$: Material cost, $\:{C}_{de}$: De-binding cost, $\:{C}_{si}$: Sintering cost, $\:{C}_{la}$: Labor cost, $\:{C}_{co}$: Consumable cost, and $\:{C}_{ot}$: Other cost.

The business purchases the processing machines used in the production process through depreciation $\:{(C}_{dep}:USD\:per\:machine)$(Eq. 3). The processing equipment runs on three phases of electricity at 230 volts $\:\left({C}_{en}:USD\:per\:machine\right)$ (Eq. 4) and the business needs to perform weekly, monthly, and other periodic maintenance on the processing machinery $\:\left({C}_{mai}:USD\:per\:machne\right)$ (Eq. 5) and as advised by the manufacturer, replace the parts (spare parts). When the processing machine is used, the aforementioned costs accumulate over time. Formula (2) computes the overall cost spent on the processing machine.

2 $$\:{C}_{ma}={C}_{dep}+{C}_{en}+{C}_{mai}\:\:\:\:\:\:\:\:\:\:\:\:\:\:\:\:\:\:\:\:\:\:\:\:\:\:\:\:\:\:\:\:\:\:\:\:\:\:\:\:\:\:\:\:\:\:\:\:\:\:\:\:\:\:\:\:\:\:\:\:\:\:\:\:\:\:\:\:\:\:\:\:\:\:\:\:\:\:\:\:\:\:$$

Where,

3 $$\:{C}_{dep}={C}_{dep,h}\times\:{T}_{p}\:\:\:\:\:\:\:\:\:\:\:\:\:\:\:\:\:\:\:\:\:\:\:\:\:\:\:\:\:\:\:\:\:\:\:\:\:\:\:\:\:\:\:\:\:\:\:\:\:\:\:\:\:\:\:\:\:\:\:\:\:\:\:\:\:\:\:\:\:\:\:\:\:\:\:\:\:\:\:\:\:\:\:\:\:\:\:\:\:\:\:$$

$$\:{C}_{dep,h}=\raisebox{1ex}{${C}_{dep,\:a}$}\!\left/\:\!\raisebox{-1ex}{${U}_{machine}$}\right.$$

$$\:{C}_{dep,\:a}=\raisebox{1ex}{$\text{P}\text{P}$}\!\left/\:\!\raisebox{-1ex}{$\text{Y}$}\right.$$

Where,$\:{T}_{p}\left(hours\right)$: Operation time per machine, $\:{C}_{dep,\:a}\:\left(USD\:per\:years\right)$: depreciation cost, $\:{U}_{machine}$: Machine uptime between 0 and 1, $\:\text{P}\text{P}\:\left(\text{U}\text{S}\text{D}\right)$: Machine cost, and $\:\text{Y}\:\left(\text{y}\text{e}\text{a}\text{r}\text{s}\right)$: Machine lifetime.

Equation 4 calculates the energy cost created at the processing machine based on the machine’s operating hours and the quantity of energy used at the processing machine’s cable supply.

4 $$\:{C}_{en}={T}_{p}\times\:{P}_{p}\times\:{c}_{0}\:\:\:\:\:\:\:\:\:\:\:\:\:\:\:\:\:\:\:\:\:\:\:\:\:\:\:\:\:\:\:\:\:\:\:\:\:\:\:\:\:\:\:\:\:\:\:\:\:\:\:\:\:\:\:\:\:\:\:\:\:\:\:\:\:\:\:\:\:\:\:\:\:\:\:\:\:\:\:\:\:\:\:\:\:\:\:\:\:\:\:\:\:\:\:\:\:\:\:\:\:\:$$

Where, $\:{T}_{p}\:\left(\text{h}\text{o}\text{u}\text{r}\text{s}\right)$: Operation time of the machine, $\:{P}_{p}\left(\text{k}\text{W}\text{h}\right)$: The ratio of the consumption power energy of the machine and $\:{c}_{0}:\left(\text{U}\text{S}\text{D}\:\text{p}\text{e}\text{r}\:\text{k}\text{W}\text{h}\right)$: Energy costs.

5 $$\:{C}_{mai}=\frac{PP\times\:{MC}_{\%}}{Effective\:operation\:hours}\times\:{T}_{p}\:\:\:\:\:\:\:\:\:\:\:\:\:\:\:\:\:\:\:\:\:\:\:\:\:\:\:\:\:\:\:\:\:\:\:\:\:\:\:\:\:\:\:\:\:\:\:\:\:\:\:\:\:\:$$

Where, $\:PP\:\left(USD\right)$: Machine unit price, $\:{MC}_{\%}$: The effective operating hours represent the total hours per year of the Unit and the percentage of maintenance costs per machine.

Formula (6) is used to determine the cost of raw materials used in product production; Formula (7) determines the cost of raw materials used in product manufacturing per unit.

6 $$\:{C}_{m}=N\times\:{v}_{m}\times\:{c}_{m}\:\:\:\:\:\:\:\:\:\:\:\:\:\:\:\:\:\:\:\:\:\:\:\:\:\:\:\:\:\:\:\:\:\:\:\:\:\:\:\:\:\:\:\:\:\:\:\:\:\:\:\:\:\:\:\:\:\:\:\:\:\:\:\:\:\:\:\:\:\:\:\:\:\:\:\:\:\:\:\:\:\:\:\:\:\:\:\:\:\:\:\:\:\:\:$$

7 $$\:{C}_{m}=N\times\:\left({v}_{m}\times\:{c}_{m}\right)+N\times\:\left({v}_{s}\times\:{c}_{s}\right)\:\:\:\:\:\:\:\:\:\:\:\:\:\:\:\:\:\:\:\:\:\:\:\:\:\:\:\:\:\:\:\:\:\:\:\:\:\:\:\:\:\:\:\:\:\:\:\:\:\:\:\:\:\:\:\:\:\:\:\:$$

Where, $\:N$: Number of parts, $\:{v}_{m}\left({cm}^{3}\right)$: The volume of material, $\:{c}_{m}\left(USD\:per\:{cm}^{3}\right)$: Cost of material per unit, $\:{v}_{s}\:\left({cm}^{3}\right)$: Volume of material support, $\:{v}_{s}\left(USD\:per\:{cm}^{3}\right)$: Cost of volume of material support.

The cost of material lost during the induction heat treatment process due to high temperatures burning or flaking the material’s surface is calculated using the formula (8).

8 $$\:{C}_{de}={V}_{de}\times\:{c}_{de}+{P}_{de}\times\:{T}_{de}\times\:{c}_{0}\:\:\:\:\:\:\:\:\:\:\:\:\:\:\:\:\:\:\:\:\:\:\:\:\:\:\:\:\:\:\:\:\:\:\:\:\:\:\:\:\:\:\:\:\:\:\:\:\:\:\:\:\:\:\:\:\:\:\:\:\:\:\:\:\:\:\:\:$$

Where, $\:{V}_{de}\left(liters\right)$: The volume of cooling fluids, $\:{c}_{de}\left(USD\:per\:liters\right)$: Cost of fluid per unit, $\:{P}_{de}\left(kW\right)$: Consumed of power, $\:{T}_{de}\left(hours\right)$: Time, and $\:{c}_{0}\left(USD\:per\:kWh\right)$: Cost of power per unit.

The Products made with Suj2 materials are subject to stringent controls during production, particularly during the induction heat treatment step. The induction heat treatment process involves numerous input factors, including voltage, amperage, cooling water volume, and concentration. Consequently, all particular aspects must be considered when determining the cost, energy consumption cost, and process protection cooling water cost. This is done using formula (9).

9 $$\:{C}_{s}=\frac{\left({V}_{\alpha\:}\times\:{c}_{\alpha\:}\right)+\left({P}_{s}\times\:{T}_{s}\times\:{c}_{0}\right)}{{N}_{s}}\:\:\:\:\:\:\:\:\:\:\:\:\:\:\:\:\:\:\:\:\:\:\:\:\:\:\:\:\:\:\:\:\:\:\:\:\:\:\:\:\:\:\:\:\:\:\:\:\:\:\:\:\:\:\:\:\:\:\:\:\:\:\:\:\:\:\:\:\:\:\:\:$$

Where, $\:{V}_{\alpha\:}\left(liters\right)$: Volume of MG815, $\:{c}_{\alpha\:}\left(USD\:per\:liters\right)$: Cost of MG815 per unit, $\:{P}_{s}\left(kW\right)$: Consumed power, $\:{T}_{s}\left(hours\right)$: Time, $\:{c}_{0}\left(USD\:per\:kWh\right)$: Cost of power per unit, and $\:{N}_{s}$: Numbers of parts.

The hourly labor rate is used to compute the cost of employing direct labor during office hours and production. Besides the laborers who handle the machine directly during production, additional personnel are responsible for machine maintenance, spare component replacement as necessary, and production management. Formula (10) is used to compute all labor costs.

10 $$\:{C}_{la}=\sum\:_{j}{T}_{j}\times\:{c}_{j}\:\:\:\:\:\:\:\:\:\:\:\:\:\:\:\:\:\:\:\:\:\:\:\:\:\:\:\:\:\:\:\:\:\:\:\:\:\:\:\:\:\:\:\:\:\:\:\:\:\:\:\:\:\:\:\:\:\:\:\:\:\:\:\:\:\:\:\:\:\:\:\:\:\:\:\:\:\:\:\:\:\:\:\:\:\:\:\:\:\:\:$$

Where, $\:{T}_{j}\left(hours\right)$: Operation time, $\:{c}_{j}\left(USD\:per\:hours\right)$: Cost of operation per unit.

The cost of consumables, including packaging, QR Code stamps, and other supplies, is determined using the formula (11).

11 $$\:{C}_{c}={\sum\:}_{i}{c}_{i}\times\:{N}_{i}\:\:\:\:\:\:\:\:\:\:\:\:\:\:\:\:\:\:\:\:\:\:\:\:\:\:\:\:\:\:\:\:\:\:\:\:\:\:\:\:\:\:\:\:\:\:\:\:\:\:\:\:\:\:\:\:\:\:\:\:\:\:\:\:\:\:\:\:\:\:\:\:\:\:\:\:\:\:\:\:\:\:\:\:\:\:\:\:\:\:$$

Where, $\:{c}_{i}\left(USD\:per\:unit\right)$: Cost per unit, $\:{N}_{i}$: Number of units.

In this study, the material used was SUJ2 bearing steel, a low-alloy tool steel with high carbon content commonly used to manufacture ball bearings, camshafts, and high-wear parts in the mechanical industry. SUJ2 steel was selected due to its high hardness, good wear resistance, and especially its mechanical properties, which can be significantly improved after induction heat treatment. Before heat treatment, the chemical composition of SUJ2 steel was determined by optical emission spectroscopy (OES), which showed that the carbon content of 0.95-1.10% enhances the hardness, while chromium (1.3-1.6%) improves the wear resistance. After heat treatment, the mechanical properties of the material changed significantly, with hardness increasing from 20 to 25 HRC to 58–63 HRC, tensile strength more than doubled, but elongation decreased due to microstructural transformation to martensite and dispersed carbides, which improved hardness but reduced ductility. This study’s induction heat treatment process was carried out on a high-frequency heating system with optimized parameters using the Six Sigma method. The sample was heated to 820–850 °C for 6.8–7.2 s, rapidly cooled with polymer solution or oil to form a martensite structure, and then tempered at 150–180 °C for 60 min to reduce residual stress and improve toughness. The key difference between this study and previous studies is the application of Active Power Filter (SAPF) to control the power quality during induction heating, which helps maintain the stability of the current and significantly improves the accuracy of the sintering temperature. Before applying SAPF, the total harmonic distortion (THDi) reached 36.35%, far exceeding the allowable level according to IEEE 519:2022, leading to large temperature fluctuations (± 50 °C) and increasing the defective rate of products up to 90%. After implementing SAPF, the THDi decreased to 5.21%; the sintering temperature was maintained within ± 5 °C, reducing the defective rate to only 1%. This improvement was validated by PLS-SEM, showing a correlation coefficient R² = 0.82, demonstrating that the current stability (improved by SAPF) strongly influences the product hardness after heat treatment. The error between the experimental and predicted model values is only less than 3%, ensuring the system’s accuracy. Thus, this study not only optimizes the induction heat treatment process but also proves that the integration of SAPF helps minimize the negative impact of power quality on product quality, opening a new approach to power control applications in modern industrial production systems.

#### Multi-attribute decision making (MADM)

Numerous outcomes of the MADM approach are also assessed subjectively in terms of ranking criteria^23^. Decision-making outcomes for the actual world are further complicated by the decision-making process in an unpredictable setting, as well as the ambiguous surroundings and lack of explicit values in MADM^24^. To address this shortcoming of MADM, the fuzzy approach was created, and its fuzzification function is also straightforward and not overly complex^25^. Nevertheless, the uncertainty in the environment with a limited number of samples and the inconsistent member or non-member functions remain, and the trio’s IF function is created to increase the fuzziness.

In this study, the Entropy method was chosen to determine the criterion weights because of its objectivity, independence from subjective assessment, and no comparison matrix required, which saves time and minimizes human error. This method is particularly suitable for MADM and fuzzy sets, supporting the processing of uncertain data in industrial production. In addition, Entropy is simple to calculate, easy to deploy, and suitable for industrial environments, especially small and medium-sized enterprises. Meanwhile, WENSLO is capable of processing highly variable data but requires strict standardization, causing complications in the application process. LOPCOW helps to adjust the weights flexibly but requires regular monitoring and updating, while CRITIC is based on the correlation between criteria but is less effective in production environments without clear linear relationships. Compared to the above methods, Entropy is more optimal because it does not require correlation evaluation between criteria, helping to reduce the complexity of the model. As a result, this method is highly effective in optimizing the decision-making process in a volatile production environment. Choosing Entropy ensures accuracy, simplifies calculations, and increases feasibility in practical applications, improving efficiency and quality in production management.

Entropy has been a key component in fuzzy methods’ development, as it has enhanced accuracy performance when working with data in ambiguous or uncertain environments. Entropy handles measurement data in unclear and practical contexts for optimal performance^26^. The entropy method implements the divergence approach, which compels us to select the appropriate evaluation criteria. Since the entropy value and the divergence rate are negatively correlated, a high divergence rate will result in a low entropy value. Options with varying weights are indicated by entropy weights. Since the entropy method does not require the implementation of comparison matrices, it is a frequently utilized and user-friendly technique across several sectors^27^.

The MADM technique aims to identify the best substitute for the current solutions. By multiplying the weights row by column for the matrix containing the matching criterion, the TOPSIS technique computes the weights using a normalized matrix^28^. Find the Euclidean distance technique between the ideal point and the negative points. Then, compute this distance using the corresponding important criteria for each measurement. For the decision matrix, the Euclidean distance index serves as the decision weight. The TOPSIS approach is incredibly effective at solving complex issues and may be used in various industrial settings^29^.

**Fig. 1:**
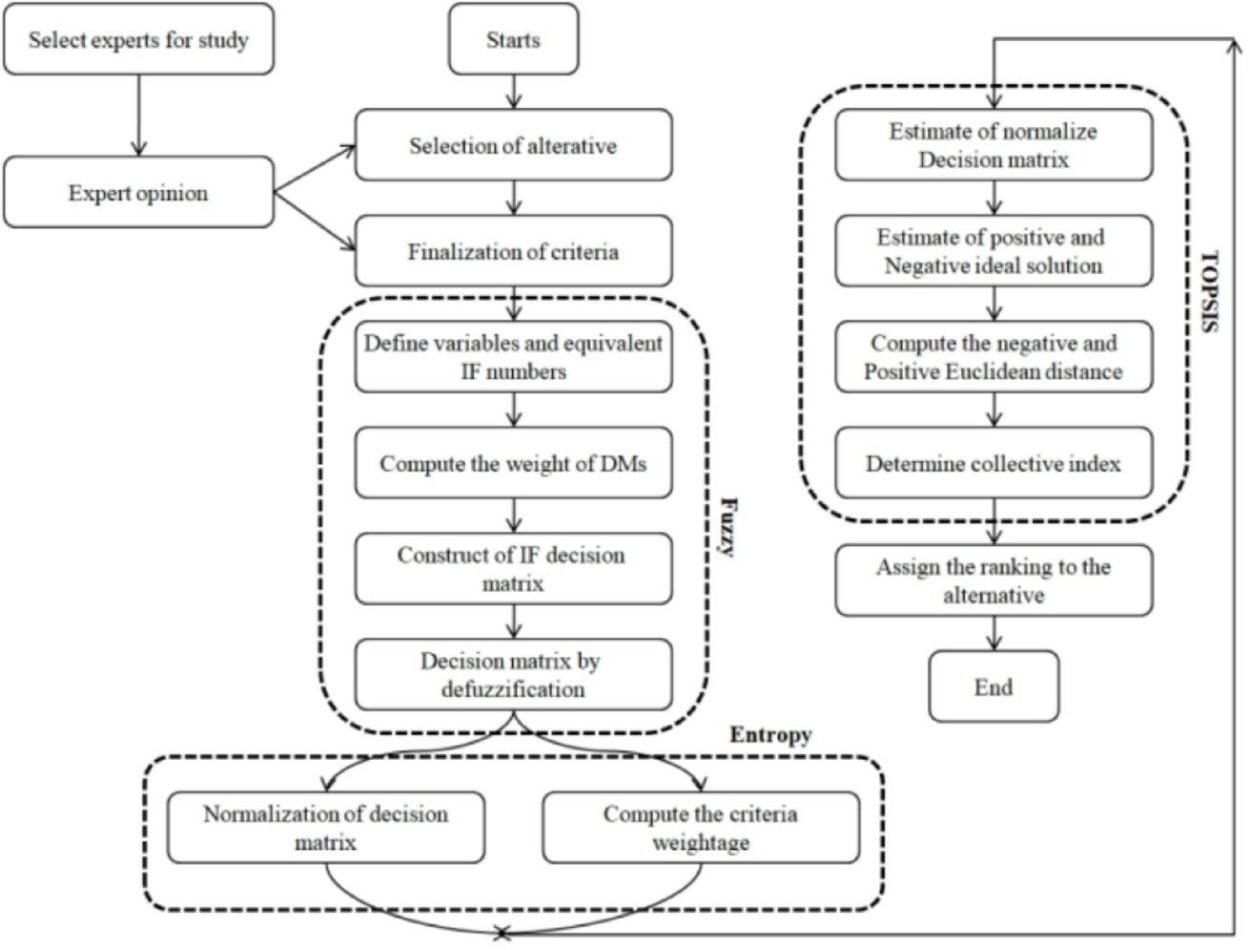
Proposed MADM methodology for Six Sigma.

In factories that produce mechanical parts, the suggested MADM approach with TOPSIS, used with the entropy of IF specified in the Six Sigma model, achieves a continual improvement of production costs (Shown in Fig. 1).

There are 2 shapes to calculate the IF index: triangular IF number (TIFN) and trapezoidal IF number (IFN). This paper selects a triangular IF number (TIFN) that meets the criteria of simplicity of use, visualization of results, representation of calculations, and simple calculations. The member function IF is calculated according to formula (12).

12 $$\:{u}_{A}\left(x\right)=\left\{\begin{array}{c}0;x<a\\\:\left(\frac{x-\text{a}}{\text{b}-\text{a}}\right)-{\uppi\:};\text{a}<x\le\:\text{b}\\\:\left(\frac{\text{c}-x}{\text{c}-\text{b}}\right)-{\uppi\:};\text{b}\le\:x<c\\\:0;x\ge\:\text{c}\end{array}\right\}\:\:\:\:\:\:\:\:\:\:\:\:\:\:\:\:\:\:\:\:\:\:\:\:\:\:\:\:\:\:\:\:\:\:\:\:\:\:\:\:\:\:\:\:\:\:\:\:\:\:\:\:\:\:\:\:\:\:\:\:\:\:\:\:\:\:$$

Where the boundary region values of TIFNs are defined as $\:(a,\:b,\:and\:c)$, with $\:a$: Lower limit, $\:b$: Middle limit, and $\:c$: Upper limit when $\:a\le\:b\le\:c$.

The IF index non-member function is calculated by the formula (13)

13 $$\:{v}_{A}\left(x\right)=\left\{\begin{array}{c}1-{\uppi\:};\:x\le\:a\\\:1-\left(\frac{x-a}{\text{b}-\text{a}}\right);\text{a}<x\le\:b\\\:1-\left(\frac{\text{c}-x}{\text{c}-\text{b}}\right);\text{b}\le\:x<c\\\:1-{\uppi\:};\:x\ge\:c\end{array}\right\}\:\:\:\:\:\:\:\:\:\:\:\:\:\:\:\:\:\:\:\:\:\:\:\:\:\:\:\:\:\:\:\:\:\:\:\:\:\:\:\:\:\:\:\:\:\:\:\:\:\:\:\:\:\:\:\:\:\:\:\:$$

Where,

$$\:0\le\:{u}_{A}\left(x\right)+{v}_{A}\left(x\right)\le\:1$$

The steps in the Hybrid Fuzzy MADM-based entropy model are detailed in Table 1.

**Table 1:** Step-by-step of the hybrid fuzzy MADM method.

Step	Explain step by step hybrid fuzzy MADM
Step 1	Define initial variables such as language variables, functional variables, and the equivalent IF index.
Step 2	Calculate the weights of the decision-making variables (Eq. 14) to determine the optimal weighting of criteria in the decision-making process by considering both positive (utility value) and negative (negative value) factors, and have the ability to customize. $\:\left({\lambda\:}_{k}\right)=\frac{\left({u}_{k}+{\pi\:}_{k}\left(\frac{{u}_{k}}{{u}_{k}+{v}_{k}}\right)\right)}{\sum\:_{k=1}^{l}\left({u}_{k}+{\pi\:}_{k}\left(\frac{{u}_{k}}{{u}_{k}+{v}_{k}}\right)\right)}\:\:\:\:\:\:\:\:\:\:\:\:\:\:\:\:\:\:\:\:\:\:\:\:\:\:\:\:\:\:\:\:\:\:\:\:\:\:\:\:\:\:\:\:\:\:\:\:\:\:\:\:\:\:\:\:\:\:\:\:\:\:\:\:\:$ (14) Where, $\:\sum\:_{k=1}^{l}{\lambda\:}_{k}=1$
Step 3	Building a composite decision matrix IF based on the opinion of Decision making (Eq. 15). Construct a synthetic intuitionistic fuzzy decision matrix by aggregating the opinions of experts based on the weights assigned to them. This method balances the attributes (positive), non-attributes (negative), and hesitation factors, ensuring a robust, data-driven, and fair multi-expert decision-making process. $$\:{r}_{ij}=\left[\begin{array}{c}1-\sum\:_{k-1}^{l}{\left(1-{u}_{ij}^{k}\right)}^{{\lambda\:}_{k}},\sum\:_{k-1}^{l}{\left({v}_{ij}^{k}\right)}^{{\lambda\:}_{k}},\:\\\:\sum\:_{k-1}^{l}{\left(1-{u}_{ij}^{k}\right)}^{{\lambda\:}_{k}},\:-\sum\:_{k-1}^{l}{\left({v}_{ij}^{k}\right)}^{{\lambda\:}_{k}}\end{array}\right]\:\:\:\:\:\:\:\:\:\:\:\:$$ (15) Where, $\:{r}_{ij}=\left(u{A}_{i}\left({x}_{j}\right),\:v{A}_{i}\left({x}_{j}\right),\pi\:{A}_{i}\left({x}_{j}\right)\right);(i=\text{1,2},3.m;j=\text{1,2},3,.n)$
Step 4	Defuzzification is the qualitative value of linguistic variables and fuzzy numbers based on decision-making (Eq. 16). It helps to convert fuzzy values ​​in the decision-making system into concrete values ​​for practical use. It not only considers attribute and non-attribute factors but also considers hesitation and adjusts data to uncertain environments. $$\:{R}^{\text{*}}\left(y\right)=\left\{\begin{array}{c}\le\:\text{a};\text{i}\text{f}\:\text{y}=0\\\:\text{a}+\left(\text{b}-\text{a}\right)\left(\text{y}+{\updelta\:}\right)\left(\sqrt{{\upmu\:}\left({\text{c}}_{1}-{\uprho\:}\right)}\right);\text{i}\text{f}\:0<y\le\:\frac{\text{x}-\text{a}}{\text{b}-\text{a}}-{\updelta\:}\\\:\text{b}\le\:\text{x}\le\:\text{c};\text{i}\text{f}\:\text{y}=1-{\updelta\:}\\\:\left(\left(\text{c}-\text{d}\right)\left(\text{y}+{\updelta\:}\right)+\text{d}\right)\left(\sqrt{{\upmu\:}\left({\text{c}}_{2}-{\uprho\:}\right)}\right);\text{i}\text{f}\:1-{\updelta\:}<y<\frac{\text{d}-\text{x}}{\text{d}-\text{c}}-{\updelta\:}\\\:\ge\:\text{d};\text{i}\text{f}\:\text{y}=0\end{array}\:\:\:\:\:\:\:\:\:\right.$$ (16) $\:\text{W}\text{h}\text{e}\text{r}\text{e},\:{\text{c}}_{1},\:{\text{c}}_{2}:\:\text{C}\text{o}\text{n}\text{s}\text{t}\text{a}\text{n}\text{t}\text{s},\:y={u}_{A}\left(x\right)+{v}_{A}\left(x\right)$
Step 5	Calculate the normalization of the decision matrix (Eq. 17) to help standardize data, bring criteria to the same scale, ensure fairness when comparing options, eliminate unit differences, increase model accuracy, and improve the reliability of the final decision. $\:{r}_{ij}=\frac{{x}_{ij}}{\sum\:_{i=1}^{m}{x}_{ij}},\:i=\text{1,2},3,.m;j=\text{1,2},3,\dots\:n\:\:\:\:\:\:\:\:\:\:\:\:\:\:\:\:\:\:\:\:\:\:\:\:\:\:\:\:\:\:\:\:\:\:\:\:\:\:\:\:\:\:\:\:$ (17)
Step 6	Calculate the value of the entropy index (Eq. 18) to measure the uncertainty of each criterion, thereby determining their importance in the decision-making model, assigning weights objectively, reducing bias, optimizing decisions, and increasing model reliability. $\:{e}_{j}=-h\sum\:_{\text{i}=1}^{\text{m}}{\text{r}}_{\text{i}\text{j}}\times\:\text{l}\text{n}\left({\text{r}}_{\text{i}\text{j}}\right),\:\text{j}=\text{1,2},.\text{n}\:\:\:\:\:\:\:\:\:\:\:\:\:\:\:\:\:\:\:\:\:\:\:\:\:\:\:\:\:\:\:\:\:\:\:\:\:\:\:\:\:\:\:\:\:\:\:\:\:\:\:\:\:\:\:\:\:\:\:$ (18) Where, $\:h=\frac{1}{ln\left(m\right)},\:m=No.of\:alternative$
Step 7	Calculate the difference value (Eq. 19) to reflect the importance of each criterion in the decision-making process. This value plays an important intermediate role in multi-criteria decision-making (MCDM) models, helping to ensure objectivity and accuracy in determining criterion weights. $\:{d}_{j}=1-{e}_{j}\:\:\:\:\:\:\:\:\:\:\:\:\:\:\:\:\:\:\:\:\:\:\:\:\:\:\:\:\:\:\:\:\:\:\:\:\:\:\:\:\:\:\:\:\:\:\:\:\:\:\:\:\:\:\:\:\:\:\:\:\:\:\:\:\:\:\:\:\:\:\:\:\:\:\:\:\:\:\:\:\:\:\:\:\:\:\:\:$ (19)
Step 8	Calculate the weighted estimate of the entropy criteria (Eq. 20) and standardize the criteria weights based on the Entropy index, ensuring that the most important criteria have higher weights. This is an objective, accurate, and efficient method for determining criteria weights in decision-making models. $\:{w}_{j}=\frac{{d}_{j}}{\sum\:_{j=1}^{n}{d}_{j}}\:\:\:\:\:\:\:\:\:\:\:\:\:\:\:\:\:\:\:\:\:\:\:\:\:\:\:\:\:\:\:\:\:\:\:\:\:\:\:\:\:\:\:\:\:\:\:\:\:\:\:\:\:\:\:\:\:\:\:\:\:\:\:\:\:\:\:\:\:\:\:\:\:\:\:\:\:\:\:\:\:\:\:\:\:\:$ (20)
Step 9	Calculate the normalized matrix value (Eq. 21) and normalize the decision matrix using Euclidean vectors, ensuring all criteria are on the same scale. This is an important step in MCDM, helping to increase accuracy, reduce errors, and ensure fairness among criteria. $\:{A}_{ij}=\frac{{\text{r}}_{\text{i}\text{j}}}{\sqrt{\sum\:_{\text{i}=1}^{\text{m}}{\left({\text{r}}_{\text{i}\text{j}}\right)}^{2}}}\:;\:\forall\:\text{j}\:\:\:\:\:\:\:\:\:\:\:\:\:\:\:\:\:\:\:\:\:\:\:\:\:\:\:\:\:\:\:\:\:\:\:\:\:\:\:\:\:\:\:\:\:\:\:\:\:\:\:\:\:\:\:\:\:\:\:\:\:\:\:\:\:$ (21)
Step 10	Calculate the value of the positive ideal ($\:{A}_{j}^{+}$) (Eq. 22) and negative ideal ($\:{A}_{j}^{-}$) (Eq. 23) solution. Identify the best and worst alternatives in the dataset. These values ​​help measure how close each alternative is to the ideal solution, allowing for more accurate and optimal decisions. $\:{A}_{j}^{+}=\left\{\left(\text{m}\text{a}\text{x}{\text{A}}_{\text{i}\text{j}},\text{j}\in\:{\text{J}}_{1}\right),\:\left(\text{m}\text{i}\text{n}{\text{A}}_{\text{i}\text{j}},\text{j}\in\:{\text{J}}_{2}\right),\:\text{i}=\text{1,2},.\text{m}\right\},\:\forall\:\text{j}\:\:\:\:\:\:\:\:\:\:\:\:\:\:\:\:\:\:\:\:\:\:\:$ (22) $\:{A}_{j}^{-}=\left\{\left(\text{m}\text{i}\text{n}{\text{A}}_{\text{i}\text{j}},\text{j}\in\:{\text{J}}_{1}\right),\:\left(\text{m}\text{a}\text{x}{\text{A}}_{\text{i}\text{j}},\text{j}\in\:{\text{J}}_{2}\right),\:\text{i}=\text{1,2},.\text{m}\right\},\:\forall\:\text{j}\:\:\:\:\:\:\:\:\:\:\:\:\:\:\:\:\:\:\:\:$ (23) Where, $\:{\text{J}}_{1}$: Higher best criteria, $\:{\text{J}}_{2}$: Lower best criteria
Step 11	Calculate the value of the Positive Euclidean distance (Eq. 24) and negative Euclidean distance (Eq. 25). Calculate the Euclidean distance between the solution and a positive ideal solution and a negative ideal solution, which helps to determine which solution is best based on the distance. $\:{D}_{i}^{+}={\left[\sum\:_{\text{j}=1}^{\text{n}}{\left({\text{A}}_{\text{i}\text{j}}-{\text{A}}_{\text{j}}^{+}\right)}^{2}\right]}^{0.5}\:\:\:\:\:\:\:\:\:\:\:\:\:\:\:\:\:\:\:\:\:\:\:\:\:\:\:\:\:\:\:\:\:\:\:\:\:\:\:\:\:\:\:\:\:\:\:\:\:\:\:\:\:\:\:\:\:\:\:\:\:\:\:$ (24) $\:{D}_{i}^{-}={\left[\sum\:_{\text{j}=1}^{\text{n}}{\left({\text{A}}_{\text{i}\text{j}}-{\text{A}}_{\text{j}}^{-}\right)}^{2}\right]}^{0.5}\:\:\:\:\:\:\:\:\:\:\:\:\:\:\:\:\:\:\:\:\:\:\:\:\:\:\:\:\:\:\:\:\:\:\:\:\:\:\:\:\:\:\:\:\:\:\:\:\:\:\:\:\:\:\:\:\:\:\:\:\:\:\:\:$ (25)
Step 12	Calculate the aggregate index estimate $\:{C}_{i}$ determine how similar each alternative is to the best and worst solutions, helping to rank the alternatives and select the optimal solution (Eq. 26). $\:{C}_{i}=\frac{{D}_{i}^{-}}{{D}_{i}^{+}+{D}_{i}^{-}}\:\:\:\:\:\:\:\:\:\:\:\:\:\:\:\:\:\:\:\:\:\:\:\:\:\:\:\:\:\:\:\:\:\:\:\:\:\:\:\:\:\:\:\:\:\:\:\:\:\:\:\:\:\:\:\:\:\:\:\:\:\:\:\:\:\:\:\:\:\:\:\:\:\:\:\:\:\:\:\:\:\:\:\:\:\:$ (26)
Step 13	Prioritize ranking based on the $\:{C}_{i}$ value to select the improvement priority value for problem points for continuous improvement according to Six Sigma. The problem points with the highest $\:{C}_{i}$ value are important problems that need to be improved as a priority.

In this study, TOPSIS (Technique for Order of Preference by Similarity to Ideal Solution) was chosen as the evaluation method because of its simplicity, objectivity, and ability to quantify the optimality of each option based on the distance to the ideal and less-than-ideal solution. This is especially useful when analyzing conflicting criteria in the system, such as production costs, product quality, energy efficiency, and employee satisfaction. Compared with other methods such as MABAC, VIKOR, MAIRCA, or MARCOS, TOPSIS has the outstanding advantages of being easy to calculate and easy to implement, suitable for real industrial production environments. Although MABAC is flexible with both quantitative and qualitative criteria, the calculation process is more complicated. VIKOR focuses on compromise solutions for conflicting but less-than-ideal criteria to find a clear, optimal solution. MAIRCA balances the ideal and the practical but is less widely used, while MARCOS, an improvement of TOPSIS and VIKOR, requires more complex data normalization. TOPSIS stands out for its simple normalization process and insensitivity to input data errors and has been widely used in Six Sigma research, quality management, and production optimization. Therefore, this method ensures accuracy and reliability and is suitable for evaluating system performance in multi-criteria production environments.

#### Partial least squares structural equation modeling (PLS-SEM)

In the study, in addition to an SEM model that is used to simulate all the paths simultaneously and in detail, a partial least squares structural equation modeling (PLS-SEM) model also contributes. (1) Despite limitations on sample size, sample size distribution, and sample size imposed by the PLS-SEM technique. (2) The PLS-SEM approach excels in assessing both non-independent variables while delivering reliable and accurate results. (3) PLS offers a strong line of defense against biased data and independent factors. There are two techniques for sampling^30^. When doing descriptive research and determining causal relationships, the probabilistic sampling method is chosen since it is error-controlled and highly representative of the population. The non-probability sampling method is utilized for visit studies since it saves time and money^31^. The probability sampling method is employed in this investigation. The quality of the sample size (Eq. 27) and the number of samples (Eq. 28) are factors considered while determining the best sampling technique for the study.

27 $$\:n=\frac{\left[q\left(1-q\right)\right]{Z}_{\raisebox{1ex}{$\alpha\:$}\!\left/\:\!\raisebox{-1ex}{$2$}\right.}^{2}}{{D}^{2}}\:\:\:\:\:\:\:\:\:\:\:\:\:\:\:\:\:\:\:\:\:\:\:\:\:\:\:\:\:\:\:\:\:\:\:\:\:\:\:\:\:\:\:\:\:\:\:\:\:\:\:\:\:\:\:\:\:\:\:\:\:\:\:\:\:\:\:\:\:\:\:\:\:\:$$

Where: $\:q$: Shows the occurrence rate of the elements in the sampling unit, exactly as the sampling target $\:0\le\:q\le\:1$.

28 $$\:n={\left(\frac{Z.s}{a.\stackrel{-}{x}}\right)}^{2}\:\:\:\:\:\:\:\:\:\:\:\:\:\:\:\:\:\:\:\:\:\:\:\:\:\:\:\:\:\:\:\:\:\:\:\:\:\:\:\:\:\:\:\:\:\:\:\:\:\:\:\:\:\:\:\:\:\:\:\:\:\:\:\:\:\:\:\:\:\:\:\:\:\:\:\:\:\:\:\:\:\:\:\:\:\:\:$$

Where: $\:Z$: The statistical value corresponds to the reliability. $\:s$: The standard deviation of the original sample. $\:\stackrel{-}{x}$: Average of the original sample. $\:a$: The sample bias rate depends on the sensitivity of the research results.

#### Case study for improvement

SUJ2 material has a ductility and hardness of less than 15 HRC before heat and induction heat treatment; the surface hardness increases from 58 HRC to 62 HRC with a low depth of induction heat treatment from 1.7 mm to 22 mm, it is used in the cylinders of the dies of heavy-duty presses due to their ductility and rigidity; SUJ2 Material has excellent bearing properties such as wear resistance, ductility toughness, and hardness. Its workability and fatigue resistance are also excellent. SUJ2 is widely used to produce plastic molds, ball bearings, steel balls, balls, bushings, shafts, guide rods, guide pins, and other mechanical parts. Chemical composition of SUJ2 according to JIS4805 (C(0.95%~1.10%), Cr(1.3%~1.60%), S(0.025%Max), Si(0.15%~0.35%), Mn(0.5%Max) ), P(0.025%Max), Cu(0.20%Max), Ni(0.25%Max), Mo(0.08%Max) and Mechanical properties of SUJ2 material: Compression modulus (140 GPa), Shear modulus (80 GPa) ), Elastic modulus (190–210 GPa), Porosity coefficient (0.27–0.30), Tensile strength (1617 (kgf/mm²)), Bending strength (1176 (kgf/mm²)), Elongation (5%), and Impact strength (28 (J/cm2)).

Thermal engineering has types such as volumetric quenching, performing a thermal process that immerses the entire product in an oil bath and heats it to a temperature of 1200 degrees C, and the material hardness reaches from 58 HRC to 62 HRC for the whole product. The Chikka type surface heat treatment performs a heat process by NITO gas, and surface hardness reaches 75 HCR to 85HRC with the Chikka penetration depth from 1.9 mm to 2.5 mm and induction heat treatment by coil process coil surrounds the product and heats it by current and voltage from the source with surface stiffness ranging from 58 HRC to 62 HRC and penetration depth from 1.7 mm to 2.2 mm.

**Fig. 2:**
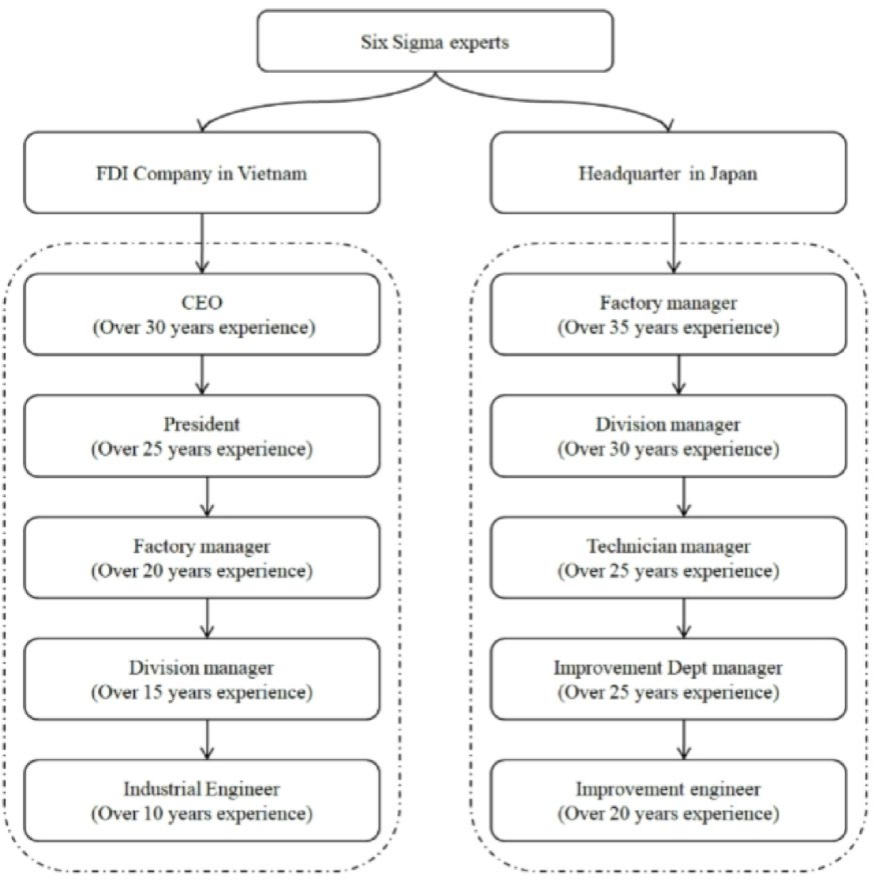
Hierarchy of selected Six Sigma experts.

Improving production business efficiency in a mechanical factory means enhancing productivity by reducing production costs at each production process. This study aims to improve the production cost of mechanical products using Suj2 raw material in an induction heat treatment process to improve production efficiency. The content of the stages of establishing criteria to improve the efficiency of the induction heat treatment process with the post-permeation size of 1.7 mm to 2.2 mm and the material hardness after the induction heat treatment. The target is from 58 HRC to 62 HRC responses according to the DMAIC (Define-Measure-Analysis-Improve-Control) cycle. The continuous improvement project of this study includes personnel including mechatronics maintenance: 2 people, high-rise heating machine operator: 4 people, induction heat treatment supervisor: 2 people, high-rise temperature data entry staff (high-rise thermal conditions, induction heat treatment test product quality, order-to-order production data to meet the production plan): 1 person, production manager: 1 person and members in the continuous improvement project under the Six Sigma program (Shown in Fig. 2). 7 criteria for production costs are listed according to the opinion of a member of a Six Sigma project doing brainstorming, including C1: Labor cost, C2: Material cost, C3: De-binding cost, C4: Sintering cost, C5: Machine cost, C6: Consumable cost, and C7: Other cost. The operator on the ground gathers data while the blank product is undergoing induction treatment to identify alternatives. 16 batches of data were gathered at 4 separate time intervals, and in total, 64 products were chosen for study based on the criteria C1, C2, C3, C4, C5, C6, and C7. These study samples were representative of various dates, and the researchers described the sampling procedures, giving instructions to the operator performing sampling during the grinding process and reassessing sample quality. These samples each correspond to 16 different product batches. Both formula A and formula B are applied to the lots. 8 lots were numbered from A1 to A8 for formula A during the study period due to a change in the formula, and the remaining 8 lots were numbered from A1 to A8 and B1 to B8 for formula B.

In line with the Japanese sales criterion that the customer is king, a Japanese foreign direct investment (FDI) company invests in Vietnam and continuously improves efficiency and quality. At a Japanese foreign direct investment (FDI) mechanical manufacturing company in Vietnam, this study carries out continuous improvement efforts under the Six Sigma project. Japan consistently leads the globe in both product quality and user reputation for its mechanical manufacturing sector. Every pale company’s organizational chart has a continuous improvement department working on several aspects of the business, such as improving worker technology proficiency, product quality, manufacturing environments, and processes. The company’s continuous improvement division has always been crucial to raising output and the caliber of the products produced.

The first stage in successfully implementing an improvement project is choosing one, which is essential to project success. If the implementation project is buried, the results will fail to fulfill the actual needs and hinder the company’s future development and present needs. The Six Sigma project is carried out according to the crucial project criteria, which makes it easier for the company’s development strategists to discern between improvement projects that are currently underway and those that have already been completed. Correlation graph tools are used in brainstorming sessions with industry professionals to assess and choose critical project selection criteria.

**Fig. 3:**
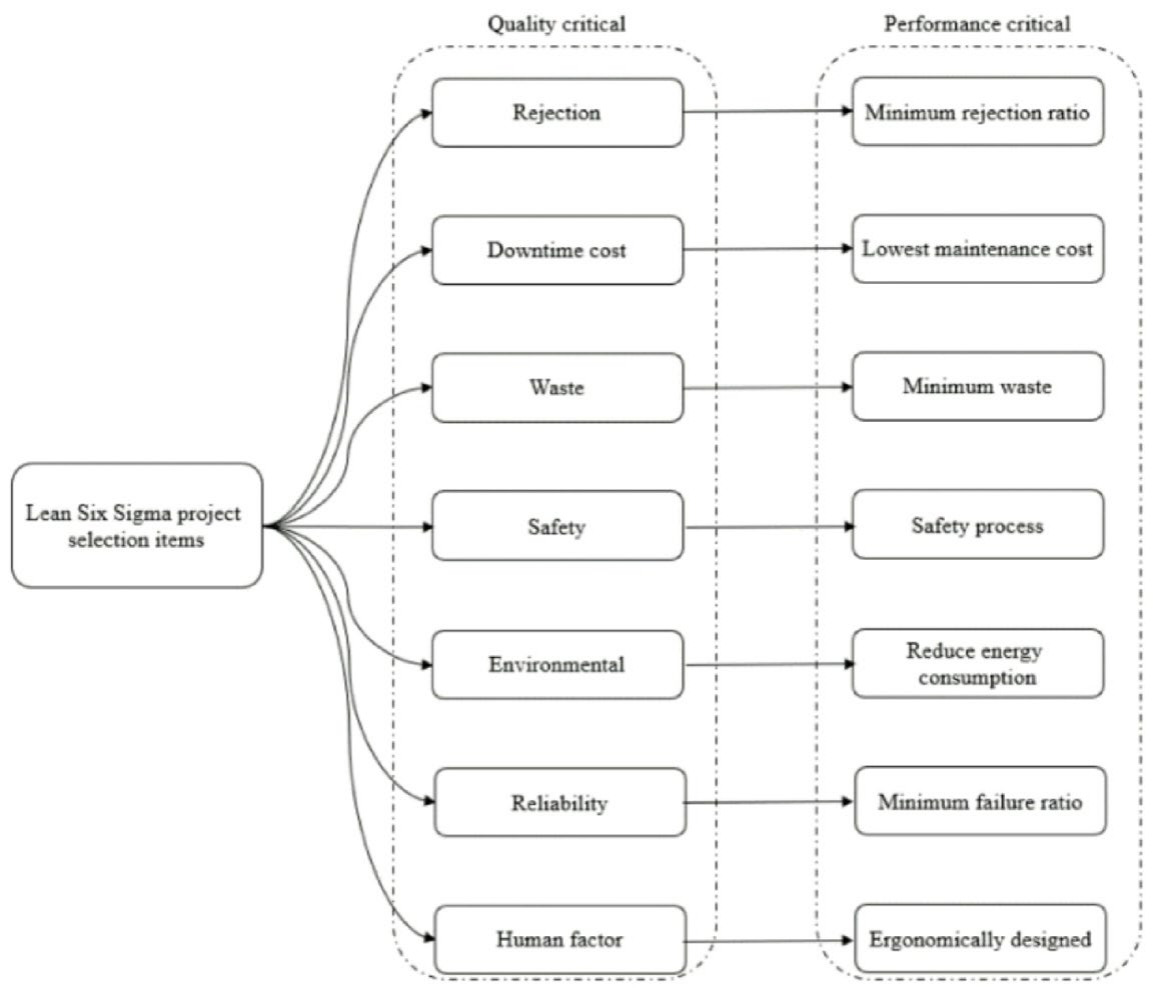
Lean Six Sigma project selection items.

Five employees of the company in Vietnam and five representatives of the parent company in Japan were among the experts invited to participate in brainstorming sessions and interviews. Every student possesses professional credentials and has worked in the relevant field for projects involving continual development for over 15 years. The suggested key criteria were used to guide the interview and brainstorming sessions by the Critical to Quality (CTQ) approach (Shown in Fig. 3), following 3 specific implementation steps as follows: Step 1: Gathering and outlining the feedback received from customers and adhering to the company’s quality policy criteria. Step 2: Conduct expert brainstorming sessions and interviews to identify the quality program and schedule components. Step 3: Determine and assess performance in achieving the company’s declared objectives and the degree of client satisfaction. Table 2 displays the performance outcomes based on the Critical to Quality (CTQ) chart.

A brainstorming session to gather ideas from relevant members with responsibilities, such as production managers, department heads, machine operators with more than 15 years of experience, and manufacturing experts, is necessary for the Six Sigma method of continuous improvement project to be implemented according to the right goals. The brainstorming session’s output must also meet the previously specified selection and exclusion criteria. These standards were developed to remove the divergent or contradictory viewpoints of specialists who assessed the qualifications of various participants in ideation sessions. Table 3 displays the results using linguistic variables, and Table 4 displays the weighted variables.

The content of giving DM variables to choose ranking criteria for golf variables is displayed in Table 5. The matrix transfer’s analysis results are based on the selection weight criterion, divergence, and entropy methods. Table 6 shows the analysis’s findings and demonstrates that the fifth parameter (Aspects of the content related to environmental consumption. For the continuous improvement project, minimum energy and minimum waste play the most important roles. The second element, which has the least relative relevance, is the downtime cost for content maintenance, activity costs, and breakdown costs.

**Fig. 4:**
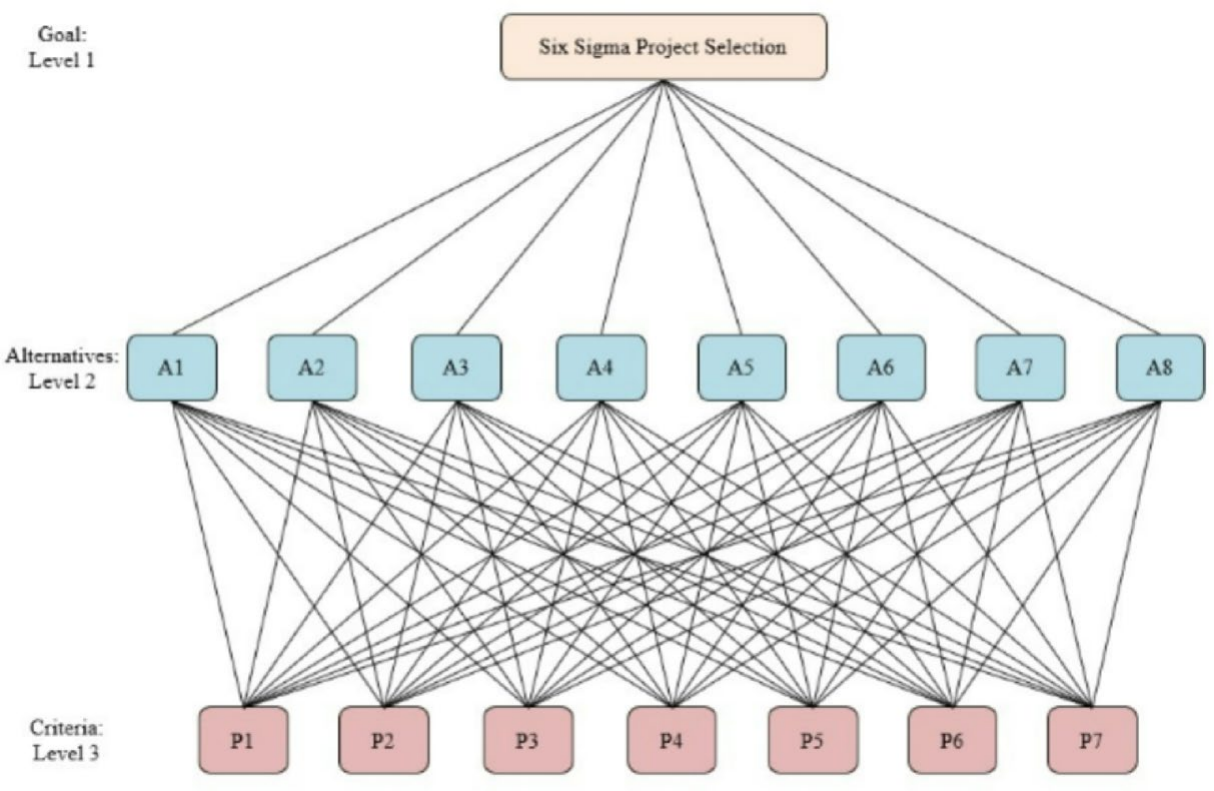
Six Sigma project selection.

The function and method for assessing Six Sigma project selection based on the standards decided upon during the brainstorming session are depicted in Fig. 4. Level 1 of the Six Sigma project establishes its objectives. Level 2 refers to the initiatives currently underway and is utilized to choose appropriate projects for improvement implementation based on predetermined standards. Level 3 displays the project possibilities along with the crucial parameters that were previously mentioned. It takes a lot of time and effort to comprehend the model of analysis and project selection based on the levels and the appropriate criteria for each level, necessitating discussion and study of the project by the analyst. Learn the methods and assess each project’s profitability.

**Table 2:** Critical to quality (CTQ) for the Six Sigma project.

Not.	Criteria	Detail explanation
P1	Rejection	Defects in the process and final inspection
P2	Downtime cost	Maintenance cost, cost of activities, and breakdown cost
P3	Human factor	Increase human efficiency by designing ergonomically
P4	waste	Material waste, non-value activities, and scraps
P5	Environment aspects	Minimum energy consumption, minimum waste
P6	Safety	A better way of operating the process
P7	Reliability	Selection reasons for the breakdown in the manufacturing place

The values of the linguistic variables are converted into fuzzy variables, and the set of fuzzy variables’ linguistic variables is created to produce selection performance values for alternatives. Table 7 lists the linguistic factors transformed into fuzzy variables and then used to assess the chosen alternatives. When it comes to choosing options for Six Sigma initiatives based on predetermined criteria, DMs are crucial in the scraper comparison matrices.

**Table 3:** Criteria for ranking by language variable.

Language variable	Code	Fuzzy numbers
Very important	VI	0.85, 0.15
Important	I	0.80, 0.15
Medium	M	0.55, 0.50
Unimportant	UI	0.45, 0.55
Very unimportant	VUI	0.15, 0.85

**Table 4:** Weighted variables.

Items	DM1	DM2	DM3	DM4	DM5	DM6	DM7	DM8	DM9	DM10
Language variable	VI	VI	I	M	VI	I	I	M	M	M
Weighted	0.150	0.150	0.105	0.050	0.155	0.103	0.105	0.059	0.059	0.059

**Table 5:** Select criteria.

Criteria	DM1	DM2	DM3	DM4	DM5	DM6	DM7	DM8	DM9	DM10
P1	VI	I	VI	I	M	M	I	M	M	M
P2	I	M	M	M	M	M	UI	M	UI	UI
P3	M	I	M	I	UI	M	M	UI	M	M
P4	I	M	I	I	M	I	VI	VI	M	VI
P5	VI	I	M	M	VI	VI	VI	I	I	I
P6	I	I	M	VI	M	UI	M	M	M	M
P7	I	I	M	M	UI	M	UI	M	M	UI

**Table 6:** Method of analysis entropy.

Measures	P1	P2	P3	P4	P5	P6	P7
e_i_	0.967834	0.873527	0.897583	0.836572	0.897865	0.897468	0.897586
d_i_	0.024365	0.013254	0.019562	0.058362	0.059789	0.019856	0.018463
w_i_	0.132532	0.046285	0.095627	0.136273	0.199879	0.094637	0.046273
Rank	3	9	5	2	1	4	6

**Table 7:** Language variables are converted into fuzzy variables.

s	Note	Fuzzy
Extremely high	EH	1.0. 0.0
Very very high	VVH	0.9, 0.1
Very high	VH	0.8, 0.1
High	H	0.7, 0.2
Medium high	MH	0.6, 0.3
Medium	M	0.5, 0.4
Medium low	ML	0.4, 0.5
Low	L	0.3, 0.6
Very low	VL	0.3, 0.7
Extremely low	EL	0.1, 0.9

Using the Fuzzy TOPSIS method’s analysis results, choose the fifth parameter corresponding to the content’s environmental consumption features. Reducing energy consumption and waste is given top priority to boost the company’s operational effectiveness through Six Sigma method-based continuous improvement initiatives.

In the high-frequency quenching heat treatment process, high current and current intensity are used to pass through the coil to quench the SUJ2 material. Good control of current and voltage factors ensures product quality, that is, no defects arise (parameter P1 (Defects in process and final inspection defects)) and, at the same time, control damaged materials. or avoid wasting raw materials in the high-temperature heat treatment process (parameter P4 (Material waste, non-value activities, and scraps)). In short, implementing good control of power quality will ensure 3 parameters: P5 (Minimum energy consumption, minimum waste), parameter P4 (Material waste, non-value activities, and scraps), and parameter P1 (Defects in process and final inspection defects). The question for this research article is whether to deploy and install a Shunt Adaptive Power filter (SAPF) device system at the source to ensure improvement of power source quality, and ensure the above requirements are an urgent study.

To evaluate the effect of Active Power Filter (SAPF) on the induction heat treatment quality of SUJ2 steel, this study used the Design of Experiments (DOE) method to ensure the objectivity and reliability of the data. The experiment was conducted with 30 SUJ2 steel samples, divided into two groups: the control group (without SAPF) and the test group (with SAPF). Each group consisted of 15 samples, providing sufficient data to evaluate the difference between the two heat treatments. The test samples were randomly selected from the same production batch, with uniform size and shape, to ensure consistency in the experiment. To minimize errors due to systematic fluctuations, each test condition was repeated three times, bringing the total number of treatments to 90 separate measurements. The experiment was conducted using a Randomized Block Design (RBD), in which the position of each sample in the heat treatment system was randomly shuffled to eliminate the influence of differences in the heating process. The measurement and analysis of the results were performed as a single-blind experiment, in which the expert evaluation team did not know in advance which samples were in the control group and which were in the test group. This helped eliminate observer bias and ensure objectivity in data recording. The parameters measured included total harmonic distortion (THDi), sintering temperature, material penetration depth, surface hardness (HRC), and defective product rate. Each parameter was measured at three different times during the production process to evaluate the stability of the heat treatment system. The collected data were analyzed using the Analysis of Variance (ANOVA) method to determine the influence of SAPF on heat treatment quality and using multiple linear regression to evaluate the relationship between factors affecting product quality. All analyses were performed at a significance level of α = 0.05, ensuring statistically significant results. In addition, the study also used an independent t-test to compare the differences between the control and experimental groups to confirm that SAPF has a significant influence on the induction heat treatment quality of SUJ2 steel.

This paper describes in detail the Active Power Filter (SAPF) design and configuration, and how to integrate it into an induction heating system to improve power quality, stabilize current, and enhance product quality. The SAPF in this study is implemented as a three-phase parallel filter, using IGBT (Insulated Gate Bipolar Transistor) technology combined with real-time feedback control, consisting of three main components: a data acquisition and measurement unit to monitor current and harmonics, a DSP (Digital Signal Processor) to analyze real-time data and calculate appropriate compensation current, and an IGBT inverter responsible for generating anti-phase compensation current to eliminate harmonic disturbances in the power system. The SAPF operates on the principle of detecting and compensating harmonics by generating anti-phase current, thereby reducing the total harmonic distortion (THDi) below the IEEE 519:2022 standard. The integration of SAPF into the induction heating process is carried out in a parallel connection model, in which the filter is directly connected to the power supply to the induction coil, which monitors and regulates the current quality without interrupting the operation of the high-frequency heating system. When THDi exceeds the standard level, the IGBT inverter in SAPF generates a compensation current to maintain a stable voltage, thereby helping to precisely control the heating temperature (820–850 °C), heating time (6.8–7.2 s), and cooling rate (20–25 °C/s) during the heat treatment process. Experimental results show that after integrating SAPF, THDi decreased from 36.35 to 5.21%, and the power factor increased from 0.89 to 0.98, helping to maintain a stable sintering temperature with a fluctuation of ± 50 °C to ± 5 °C, thereby reducing the defective product rate from 90% to only 1%. This proves that SAPF not only plays an important role in improving power quality but also helps to improve the heating process accuracy, optimize the hardness and hardenability of materials, reduce energy consumption, and prolong the life of high-frequency sintering equipment. Integrating SAPF into the induction heat treatment process is a breakthrough in combining advanced power filtering technology and optimizing the production process, improving efficiency and quality in the modern metallurgical industry.

## Result

### Material and induction heat treatment process

The steel being discussed here contains elements like Fe, C, and other alloys like Cr and Mn. The ratio of C components to other alloys will affect the characteristics of steel. The hardness and wear resistance of the material will rise with an increase in carbon content following heat treatment.

Heat treatment indicates that the metal’s structure will alter at temperatures higher than the threshold of 723 °C. It is known as the Ostenite structure at that time (the temperature threshold varies with carbon content). In this scenario, the embryo will harden if the temperature drops abruptly. After that, the material structure becomes a Martensite structure^32,33^. If the embryo in the aforementioned situation is cooled gradually, it will soften. This is how the RAM works. Ferrite + Cementite is the name of the metal structure.

**Fig. 5:**
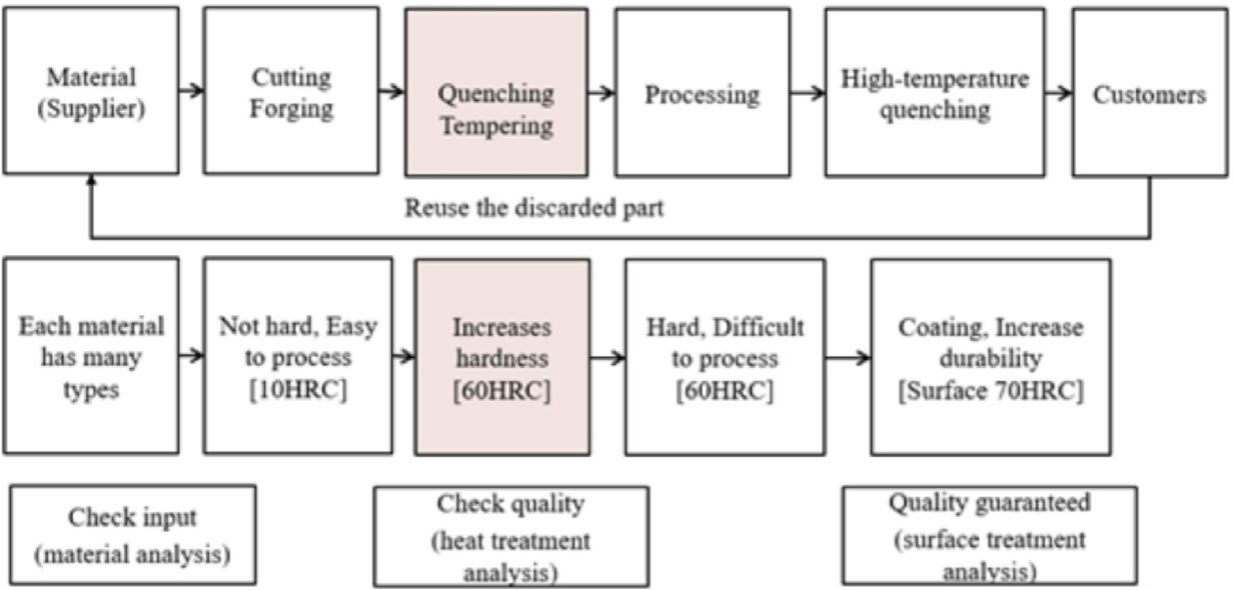
Quenching heat process.

**Fig. 6:**
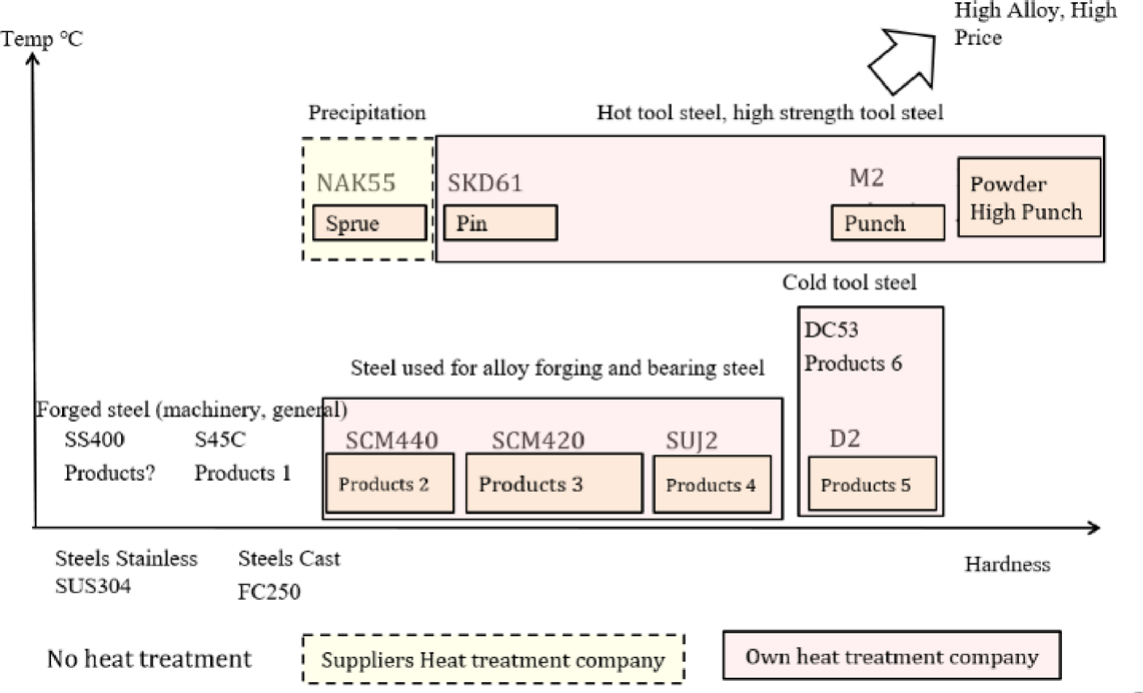
Types of quenching heat for material.

The alloy used to make SUJ2 steel raw material contains trace carbon and iron. Its inexpensive cost and ability to modify its hardness depending on heat treatment conditions make it suitable for use in various industries^34^. (Building, auto assembly, molding (screws, screws) (Shown in Fig. 5). Steel with a high carbon content is classified as a toughened material, as are many iron alloys. The company uses SUJ2 material, a type of steel used for induction heat treatment, for alloy forging and bearing steel (Fig. 6). When manufacturing mechanical products, induction heat treatment is considered a unique technique since, following the product’s thermal process, the interior structural components of the product are heated to a certain temperature. After heat processing, the material body changes, and there is no reliable testing apparatus to determine the degree of structural alterations in the material body^35,36^. Because assuring the thermal process’s quality also ensures the quality of the products, managers must consider this while selecting whether to use the thermal process in the organization.

**Fig. 7:**
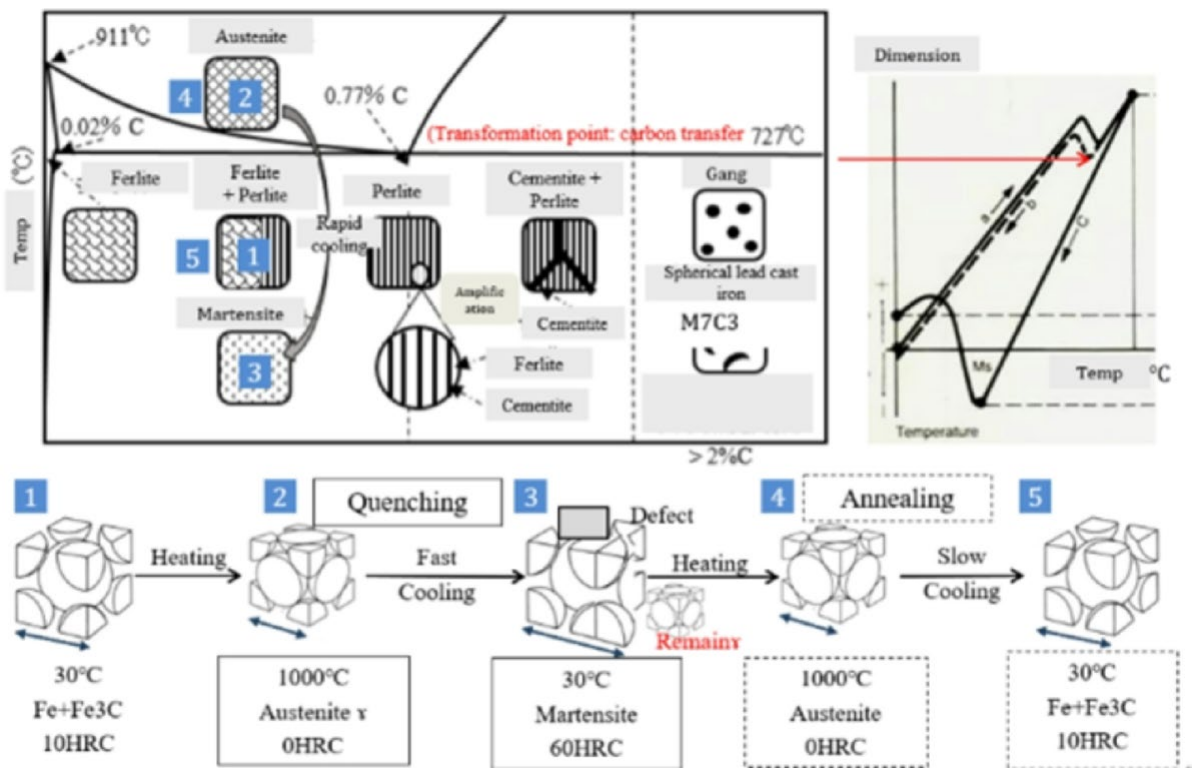
Heat treatment conditions.

By carefully applying heat, materials can have their physical and occasionally chemical properties changed^37^. This process is known as heat treatment. While it can be applied to other materials such as glass and ceramics, its most common application is in metallurgy (Shown in Figure 7). Reducing internal stresses and improving the material’s mechanical properties, such as its strength, toughness, ductility, and hardness, are the main objectives of heat treatment^38^. The material is heated to a specific temperature, held there for a given period, and then cooled gradually.

**Fig. 8:**
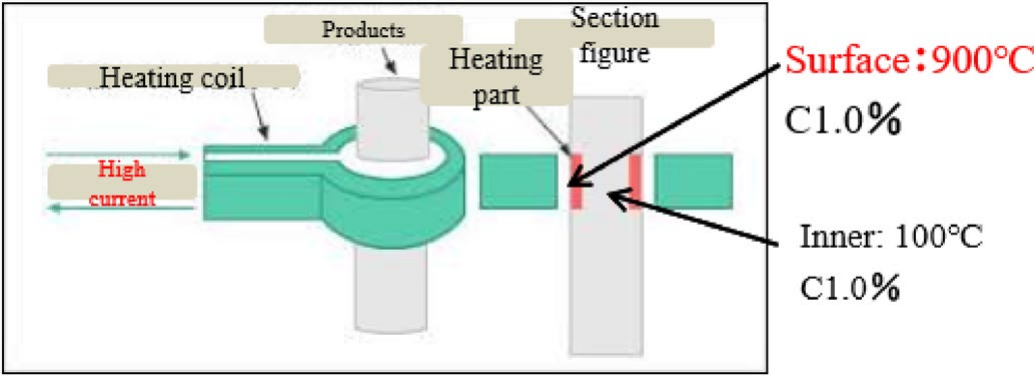
Induction heat.

Surface treatment hardens the surface to increase wear resistance. The induction heat treatment process is carried out at the company using SUJ 2 material and the induction heat treatment technology. Surface treatment hardens the surface to increase wear resistance. SUJ 2 materials undergo induction heat treatment at the company using the Induction heat treatment method (Shown in Fig. 8). A specific kind of heat treatment called induction heat treatment involves using electromagnetic induction to heat a workpiece and alter its characteristics. Its excellent heat-treatment process control and localized heating make it widely used in industries including manufacturing, aerospace, and the automotive sector.

During induction heat treatment, a copper coil is subjected to an alternating current (AC), creating a rapidly varying magnetic field. The magnetic field creates eddy currents inside a conductive workpiece when inserted into the coil. Because of the material’s resistance, these eddy currents, in turn, produce heat^39,40^. Among the main benefits of induction heat treatment are Quick and accurate heating: Certain sections of a workpiece can be quickly and precisely heated thanks to induction heating. The heat may be precisely regulated by varying the alternating current’s power and frequency, enabling effective and focused heat treatment^39^. Energy efficiency: Because induction heating heats the workpiece directly and doesn’t require heating the surrounding area, it is a very efficient method of heating. Compared to traditional methods, it decreases energy loss and allows for faster heating cycles. Decreased distortion: Local heating is usually used in induction heat treatment, which helps to reduce thermal distortion in the workpiece. It lessens the possibility of warping or deformation that might happen with uniform heating techniques by heating particular areas selectively. Automation and process control: Incorporating induction heat treatment into automated production lines is simple. The heating process can be accurately regulated, observed, and modified to guarantee reliable outcomes and satisfy particular needs. Versatility: A large variety of materials, including alloys, polymers, and composites, as well as ferrous and non-ferrous metals, can be subjected to induction heat treatment. It can be applied to several heat treatment procedures, including stress relief, brazing, annealing, tempering, and hardening. Common uses for induction heat treatment include brazing or soldering metal components, heat treating automotive components, including crankshafts and camshafts, and hardening the surfaces of gears, shafts, and tools. It’s important to remember that induction heat treatment calls for specific tools and knowledge. To get the required effects, the heat treatment parameters such as power, frequency, and heating time must be carefully chosen depending on the characteristics of the material and the intended consequences.

Here are some crucial things to consider to guarantee high-quality power for induction heat treatment: Stable voltage: To ensure efficiency and consistency, a power source must be steady for most of the heat treatment process using induction. The voltage must be kept within the designated power supply range for the induction device. It is essential to use a voltage stabilizer or regulator to control and uphold a steady voltage. Correct current: Verify that the induction heat treatment device receives the proper current according to its specifications. It is essential to find the maximum current the device can handle and ensure the power source can supply it without overloading or failing. Stability and quality of the electric current supply are two more crucial elements. The power supply must be able to deliver a steady current without producing any noise or distortion in the current. Power quality can be enhanced by utilizing protective devices like decoupling transformers and noise filters. Power-related factors: When operating, induction heat treatment may need a lot of power. Verifying that the power source can supply enough power for the induction heat treatment device is essential. The effectiveness and outcomes of the heat treatment process may be impacted if the capacity is insufficient. Regular upkeep and inspection: Periodic inspections and maintenance are required to guarantee the power source’s dependability and efficiency. Examine electrical characteristics, including voltage, current, and current quality, to find issues early and take the required maintenance or remedial action. In summary, stable voltage management and maintenance, proper current, current stability and quality, sufficient capacity, testing, and routine maintenance are all necessary to provide power quality for induction heat treatment. This guarantees quality outcomes in the finished product and aids in ensuring efficiency and uniformity in the heat treatment process.

The power supply for induction heat treatment might have several frequent problems. Among these problems are Variations in voltage: Variations in input voltage have the potential to impact the efficacy and uniformity of induction heating. Reduced overall process control, irregular heating periods, and changes in heating patterns can all arise from the unstable voltage applied to the induction heating apparatus. Electricity supply capacity: The electricity needed for induction heat treatment equipment is substantial. Insufficient power supply capacity may result in insufficient heating, sluggish heating rates, or an inability to reach the appropriate temperature. Inadequate power supply capability might also result in equipment failures or circuit breakers tripping. Harmonic distortion: The power supply system may be subjected to harmonic currents from induction heating equipment. This can result in voltage distortion and impact the functionality of other electrical devices connected to the same system. Increased losses, a lower power factor, and interference with other delicate equipment can result from harmonic distortion. Low power factor: The ratio of actual power to apparent power is known as the power factor, and it is frequently low in induction heating systems. A system with a poor power factor requires more current from the power source, which can lead to greater energy expenses and distribution network overload problems. Electrical noise and interference: Induction heating has the potential to produce electromagnetic interference (EMI) and electrical noise that may disrupt nearby communication or other electrical systems or equipment. To reduce electromagnetic interference (EMI) and guarantee that other equipment operates as intended, electromagnetic compatibility (EMC) precautions should be taken. Requirements for the cooling system: Since induction heat treatment equipment produces a lot of heat while operating, sufficient cooling systems are required to keep it operating at peak efficiency and avoid overheating. Inadequate cooling can result in equipment malfunctions, poor performance, and shorter component lifespans. It is critical to have a reliable power source with sufficient capacity for the induction heat treatment equipment to minimize these problems. Reliability and consistency can be ensured through routine maintenance and monitoring of the power supply, which includes harmonic filtering, power factor correction, and voltage regulation. It is also important to use appropriate shielding, grounding, and isolation methods to reduce electrical noise and interference. Furthermore, implementing efficient cooling systems and following advised operating conditions can aid in preventing overheating and equipment malfunctions.

### Analysis of the current status of power source quality at induction heat treatment process

The point-of-common coupling (PCC), which is generally defined as the interface between the system owner or operator and user, is where IEEE Std. 519:2022 sets limits for harmonic voltages and currents (Shown in Table 8). The PCC is typically the power system point closest to the user where the system owner or operator could offer service to another user. In this evaluation, the PCC was regarded as the transformers’ outgoing.

**Table 8:** IEEE Std. 519:2022 limits voltage THD (total harmonic distortion) (Eq. 29) to a maximum at PCC points.

Bus voltage V at PCC	Individual harmonic (%)	Total harmonic distortion THD (%)
$\:V\le\:1.0\:kV$	5.0	8.0
$\:1\:kV<V\le\:69\:kV$	3.0	5.0
$\:69\:kV<V\le\:161\:kV$	1.5	2.5
$\:161\:kV<V$	1.0	1.5^a^

29 $$\:THDv=\frac{\sqrt{\sum\:{V}_{h}^{2}}}{{V}_{t}}\:\:\:\:\:\:\:\:\:\:\:\:\:\:\:\:\:\:\:\:\:\:\:\:\:\:\:\:\:\:\:\:\:\:\:\:\:\:\:\:\:\:\:\:\:\:\:\:\:\:\:\:\:\:\:\:\:\:\:\:\:\:\:\:\:\:\:\:\:\:\:\:\:\:\:\:\:\:$$

The magnitude of each voltage harmonic cannot exceed 3% of the fundamental voltage. The IEEE Std. 519:2022. Table 9 lists the current distortion limits, which differ for each system based on its size relative to the utility system feeding it. The short-circuit ratio (SCR), which is the ratio of the maximum demand load current (IL) to the available short-circuit current (ISC), is used to quantify this relative magnitude. The facility’s size to the system capacity increases with a decreasing SCR value, as do the associated harmonic current limitations. Furthermore, current distortion levels are determined by IL, as opposed to voltage distortion levels, which are determined by the magnitude of the fundamental voltage.

**Table 9:** Current distortion limits for general distribution systems (120 V through 69 kV).

Current distortion limits for general distribution systems (120 V Through 69 000 V)						
Maximum harmonic current distortion in percent of I_L_						
Individual harmonic order (Odd Harmonics)						
$$\:\raisebox{1ex}{${I}_{sc}$}\!\left/\:\!\raisebox{-1ex}{${I}_{L}$}\right.$$	$$\:<11$$	$$\:11\le\:h<17$$	$$\:17\le\:h<23$$	$$\:23\le\:h<35$$	$$\:35\le\:h$$	TDD
$\:<{20}^{*}$	4.0	2.0	1.5	0.6	0.3	5.0
20 < 50	7.0	3.5	1.5	1.0	0.5	8.0
50 < 100	10.0	4.5	2.5	1.5	0.7	12.0
100 < 1000	12.0	5.5	4.0	2.0	1.0	15.0
> 1000	15.0	7.0	5.0	2.5	1.4	20.0

* All power generation equipment is limited to these values of current distortion, regardless of actual Isc/IL.

Where:

I_sc_ = maximum short-circuit current at PCC. I_L_ = maximum demand load current (fundamental frequency component) at PCC.

Total current distortion limits are based on the total demand distortion (TDD) level (Eq. 30). Individual current harmonics are also calculated as a percent of the maximum demand current. Nonlinear loads typically produce the maximum amount of harmonic current at peak load levels, so the maximum TDD values are given in IEEE Std. 519:2022 effectively limits the total harmonic current a given facility can inject into the power system. IEEE Std. 519:2022 calls for ID to be calculated based on the peak demand level recorded over the previous 12-month period. Nevertheless, in this assessment, all the loads are assumed to run at full load during the monitoring period. Thus, the value for IL at each location was assumed to be the maximum current drawn by the loads.

30 $$\:TDD=\frac{\sqrt{\sum\:{I}_{h}^{2}}}{{I}_{f}}\:\:\:\:\:\:\:\:\:\:\:\:\:\:\:\:\:\:\:\:\:\:\:\:\:\:\:\:\:\:\:\:\:\:\:\:\:\:\:\:\:\:\:\:\:\:\:\:\:\:\:\:\:\:\:\:\:\:\:\:\:\:\:\:\:\:\:\:\:\:\:\:\:\:\:\:\:\:\:\:\:\:\:\:\:\:\:\:\:\:\:\:\:$$

The requirements for the 2015 Viet Nam Distribution Code and the 30/2019 requirements. Total Harmonic Distortion (THD_V_) of voltage (Shown in Table 10) and Current Total Harmonic Demand Distortion TDD (Shown in Table 11).

**Table 10:** Total harmonic distortion (THD_V_) of voltage.

Voltage level	Total harmonic distortion limited	Individual harmonic order limited
High voltage	3.0%	1.5%
Medium voltage	5.0%	3.0%
Low voltage	8.0%	5.0%

**Table 11:** Current total harmonic demand distortion TDD.

Voltage level	Total Harmonic Distortion Limited	Individual Harmonic Order Limited
High voltage	4.0%	3.5%
Medium voltage	8.0%	7.0%
Low voltage	For the load ≥ 50 kW: The TDD does not exceed 12% For the load < 50 kW: The TDD does not exceed 20%	For the load ≥ 50 kW: The TDD does not exceed 10% For the load < 50 kW: The TDD does not exceed 15%

**Fig. 9:**
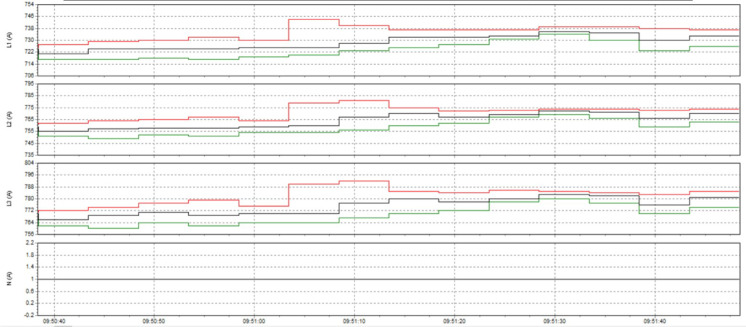
Current trends.

**Fig. 10:**
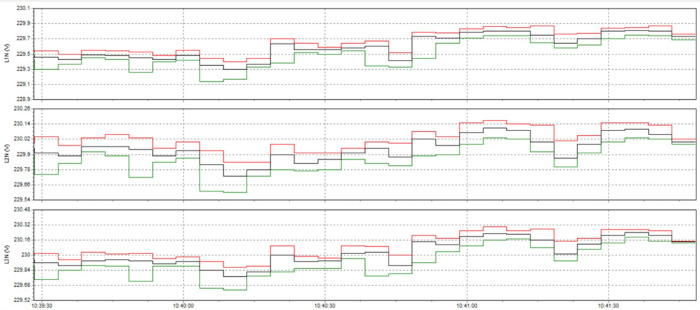
Voltage trend.

**Fig. 11:**
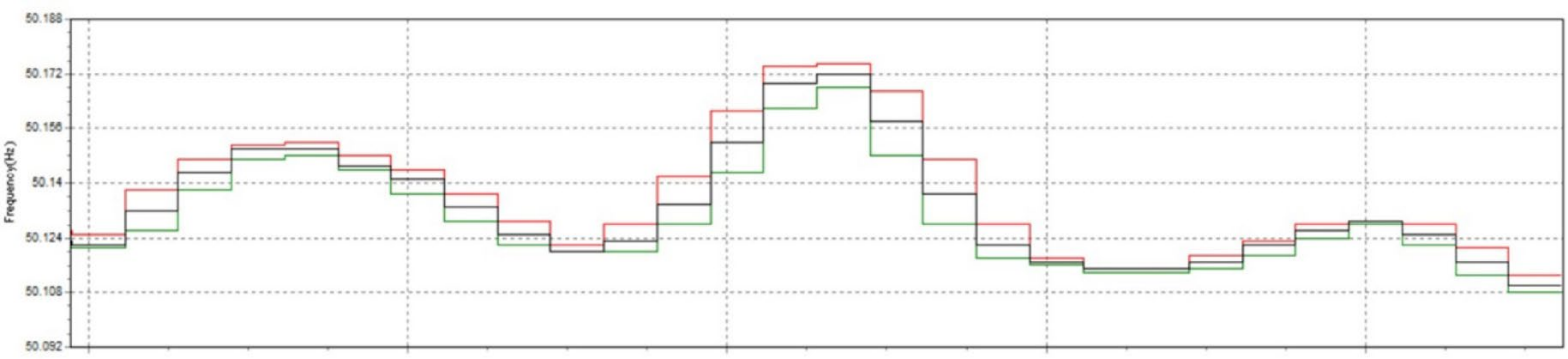
Frequency trend.

**Fig. 12:**
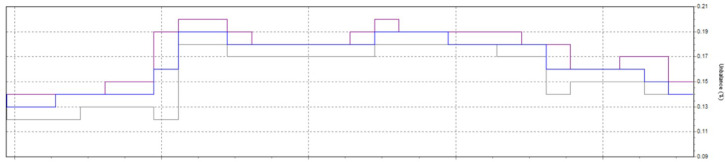
Voltage unbalance.

**Fig. 13:**
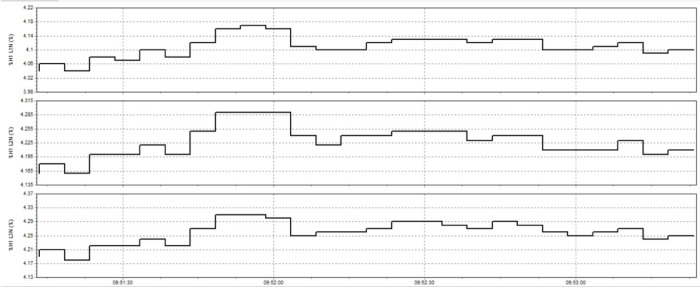
THD – Voltage.

**Fig. 14:**
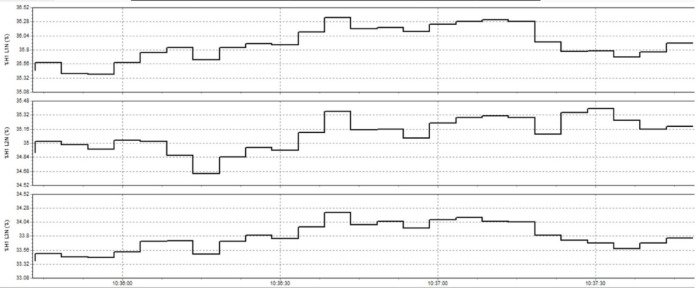
THD – current.

**Fig. 15:**
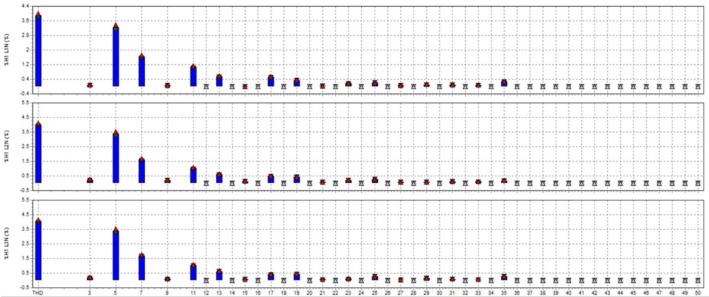
Voltage harmonic spectrum from 2nd to 50th.

**Fig. 16:**
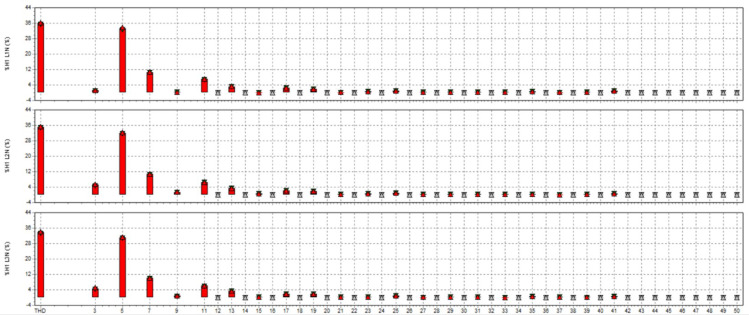
Current harmonic spectrum from 2nd to 50th.

**Fig. 17:**
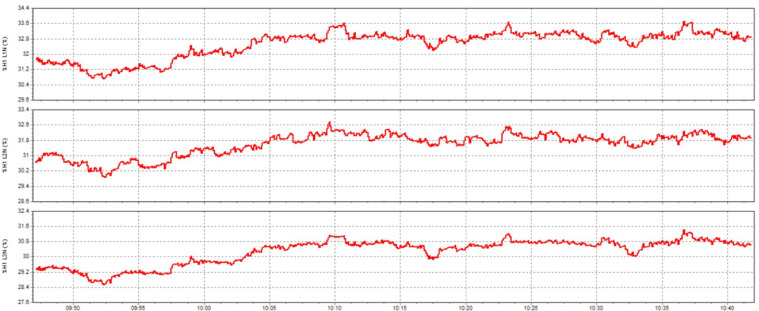
Current harmonic order 5th trend.

**Fig. 18:**
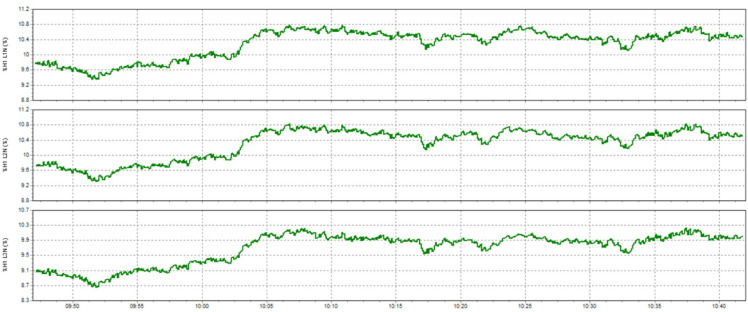
Current harmonic order 7th trend.

**Fig. 19:**
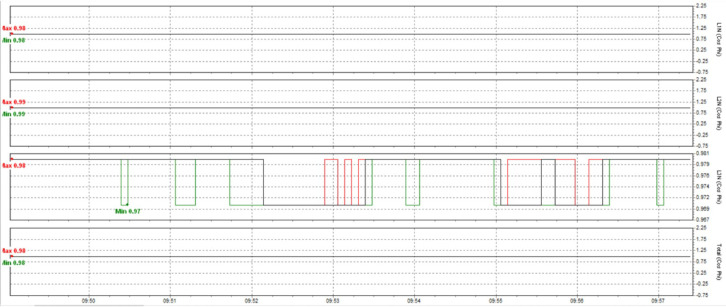
Cos Phi trend.

**Fig. 20:**
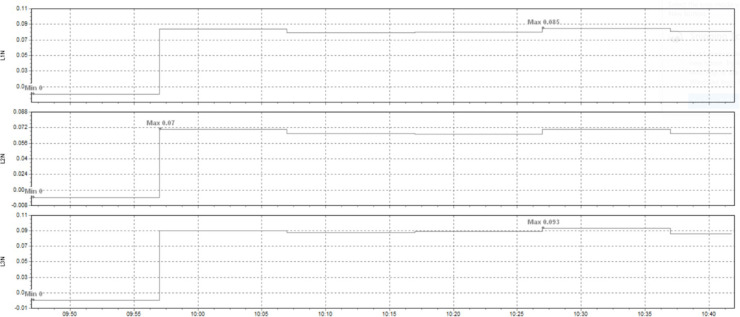
Flicker.

To control the actual value of the quality of the power supply for the high-stage quenching machine and the power supply directly to the coil used for high-stage quenching for products using SUJ 2 type materials. Results found that the quality of the power supply did not meet IEEE 519:2022 standards. Specific data measured and analyzed at the source are displayed in detail as Current trend (Shown in Table 12; Fig. 9. Current trend), Voltage trend (Shown in Table 13; Fig. 10. Voltage trend), Frequency Trend (Shown in Table 14; Fig. 11. Frequency Trend), Voltage Unbalance (Shown in Table 15; Fig. 12. Voltage Unbalance), THDv (Shown in Table 16; Fig. 13. THD – Voltage), THDi (Shown in Table 17; Fig. 14. THD – Current), Voltage harmonic spectrum from 2nd to 50th (Fig. 15. Voltage harmonic spectrum from 2nd to 50th ), Current harmonic spectrum from 2nd to 50th (Fig. 16. Current harmonic spectrum from 2nd to 50th ), Current harmonic order 5th trend (Fig. 17. Current harmonic order 5th trend), Current harmonic order 7th trend (Fig. 18. Current harmonic order 7th trend), Cos Phi (Fig. 19. Cos Phi trend), and Flicker (Fig. 20. Flicker).

Analyze the total analytical parameters of the quality of the power supply at the source for the high-rise quenching heat treatment machine and the equipment used in the production process. The results show that the actual value of the Current Harmonic THDi parameter is mostly above 34% for all phases and exceeds the allowable standard of the application standard & Evaluation Circular 39/2015 and 30/2019/BCT, which is less than 12%. The actual value of the Individual current harmonic parameter at Order 5th is 33.03%; at Order 7th, it is 10.64%, all of which are larger than the application standard & Evaluation Circular 39/2015 and 30/2019/BCT require smaller. 10% (Shown in Table 18).

The research question that needs to be asked is to design and implement a plan to improve the quality of the power source to eliminate harmonic waves at the source, to help improve the quality of the power source to meet IEEE 519:2022 standards, helping to increase the life of the power source. Operating life and accuracy of processing machines, specifically high-stage quenching heat treatment machines, help ensure product quality, reduce defect rates, and reduce energy loss to help improve operating efficiency, production, and business activities, reducing production costs. A device called SAPF is proposed to be deployed at the source.

**Table 12:** Current trend.

Name	Date	Time	Min	Avg	Max	Units
A rms	25/05/2024	09:51:08	720	725	744	A
B rms	25/05/2024	09:51:13	756	767	781	A
C rms	25/05/2024	09:51:13	767	777	792	A

**Table 13:** Voltage trend.

Name	Date	Time	Min	Avg	Max	Units
A - N rms	25/05/2024	15:08:09	229.65	229.75	229.87	V
B - N rms	25/05/2024	15:08:09	230.03	230.11	230.17	V
C - N rms	25/05/2024	15:08:09	230.16	230.23	230.3	V

**Table 14:** Frequency trend.

Name	Date	Time	Min	Avg	Max	Units
f	25/5/2024	09:55:13	50.108	50.142	50.175	Hz

**Table 15:** Voltage unbalance.

Name	Date	Time	Min	Avg	Max	Units
Unbal Vn	25/5/2024	09:55:13	0.18	0.19	0.2	%

**Table 16:** THDv.

Name	Date	Time	Max	Units
THDv AN	25/05/2022	09:51:53	4.17	%f
THDv BN	25/05/2022	09:51:53	4.29	%f
THDv CN	25/05/2022	09:51:53	4.31	%f

**Table 17:** THDi.

Name	Date	Time	Max	Units
THDi A	25/05/2024	10:36:43	36.35	%f
THDi B	25/05/2024	10:36:43	35.80	%f
THDi C	25/05/2024	10:36:43	34.21	%f

**Table 18:** Summary assessment data collected.

Summary assessment data collected in May 2024 with Maximize value				
Parameters		Value	Application Standard & Evaluation	
			Application Standard & Evaluation Circular 39/2015 and 30/2019/BCT require	Evaluation
Cosphi	A	0.98	> 0.9 (Total)	Passed
	B	0.99	> 0.9 (Total)	Passed
	C	0.98	> 0.9 (Total)	Passed
Unbalance	V_unb_	0.2%	< 5%	Passed
Voltage Harmonic THDv	A	4.17%	< 8%	Passed
	B	4.29%	< 8%	Passed
	C	4.31%	< 8%	Passed
Current Harmonic THDi	A	**36.35%**	< 12%	**Failed**
	B	**35.80%**	< 12%	**Failed**
	C	**34.21%**	< 12%	**Failed**
Individual current harmonic	Order 5th	**33.03%**	< 10%	**Failed**
	Order 7th	**10.64%**	< 10%	**Failed**
Flicker	Pst	0.093	< 1.0	Passed
	Plt	0	< 1.0	Passed

Significant values are in bold.

**Fig. 21:**
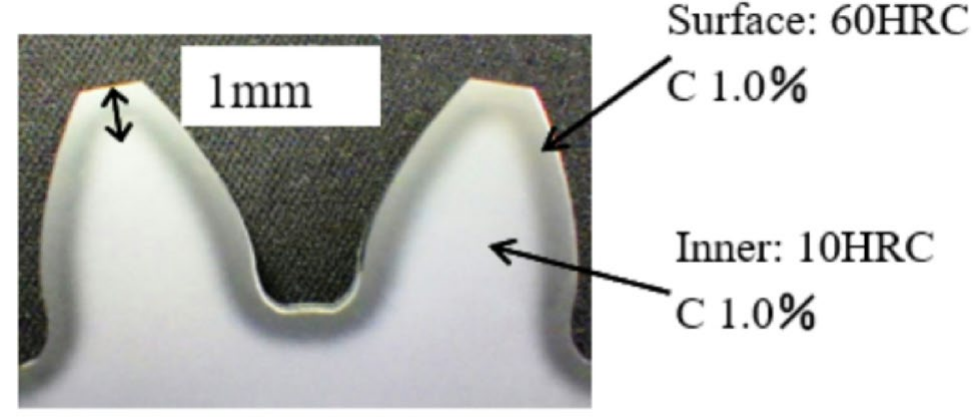
The hardness of the depth case.

A form of surface heat treatment known as induction heat treatment softens the surface of SUJ2 steel material, with penetration sizes ranging from 1.0 to 1.5 mm and hardness levels between 58 and 62 HRC at the penetration depth (Shown in Fig. 21). If the coil receives excessive voltage or current, the hardness in the deep penetration area above the 62 HRC threshold results in surface fractures or cracks on the product. The product will soften and warp if the voltage or current input to the coil is too low, resulting in a deep penetration zone hardness of less than 58 HRC. Two other critical factors influencing the penetration depth and stiffness at the product’s penetration depth area are the heating duration and the separation between the coil surface and the outer body surface during induction. To increase product quality throughout the induction heating process, the ideal conditions for the voltage or current source, timing, and distance must be designed. To determine the ideal Induction heat treatment condition, the author performs experimental optimization analysis using the Taguchi optimization approach^41^.

### Material and experimental method

The Taguchi method is an experimental design technique that aims to minimize the impact of uncontrollable elements and optimize the production process. This technique was created by Japanese statistician and engineer Genichi Taguchi, who maintained that design plays a more significant role in quality control than manufacturing processes to prevent production deviations. Orthogonal sequences are used in experimental planning using the Taguchi method. As a result, this methodology enables the analysis of the influence of parameters on a certain chosen response of a process (or product) with the least amount of tests required, allowing for the fastest possible adjustment of the parameters toward the optimum. The Taguchi method also prioritizes minimizing losses to society from detrimental side effects and considers if a product’s design may have an unfavorable impact.

The procedure of induction heat treatment involves applying an electric current to the carbon structural elements on the surface of SUJ2 material to alter their hardness and the depth to which the heat zone penetrates, hence altering the mechanical properties of the raw material. Hardness, yield strength, and ultimate tensile strength are three variables that influence the effectiveness of this induction process and the requirement for ideal control. By adjusting the input impact parameters, such as electric current (A), coil travel time (T), and contact distance between the coil and the surface of the SUJ 2 material (d), the process of altering the mechanical and physical characteristics of the carbonate component on the material’s surface can be stabilized. Tensile strength (TS), depth case area size (t), and product surface hardness (HRC) are the findings of the product quality assessment following induction. There are two stages to the implementation of the Taguchi optimization method. Because the weak parameters include three condition parameters at each of the three levels, the first stage of handling noisy parameters is conducted in nine experiments using the L9 orthogonal grid criterion. Phase 2 applies the gray relational analysis (GRA) method to multi-objective optimization.

A Chinese source provided the steel material sample used in the type SUJ2 experimental investigation. The chemical element composition is shown in the study paper’s upper section. Stream oil is the coolant utilized in this investigation. Hardness testers, face grinders, and sample cutters are ready for experimental research, and test findings adhere to JIS standards. To conduct an experimental study using 80 A, 90 A, and 100 A as the initial electric current preparation parameters. The coil travel time during induction is 5 s, 7 s, and 10 s, and the coil surface is 3 mm, 5 mm, and 7 mm from the SUJ2 material surface. The product’s hardness, penetration depth, and tensile strength after cooling are the test findings of the mechanical change of SUJ2 material following Induction heat treatment.

According to the JIS hardness measurement value, a hardness test with a 500 N load is conducted for one measurement over a 15-second operating period using the Vicker Laryee HVM-30 hardness tester. The hardness measurement results are determined using Eq. (31), and this hardness value permits the conversion of the unit to hardness using HRC units according to JIS standards.

31 $$BHN = \frac{{2F}}{{\pi D\left( {D - \sqrt {D^{2} - d^{2} } } \right)}}~~~~~$$

Where, F: Force (N), D: Diameter (mm), d: average dimension (mm).

Tensile testing is carried out following JIS specifications using the Laryee UE34200 Computer Control Electronic Universal Testing Machine (200KN,0.4%--100% F.S., Floor type). In tensile testing, the product is stretched until it splits into two pieces, at which point the measurement’s experimental outcomes are recorded. Data from tensile measurements is directly entered into the Laryee UE34200 Computer Control Electronic Universal Testing Machine’s specific software.

Two test methodologies are combined with in-depth case testing. One method is to measure the depth case size area at a distance of 0.01 mm using a Vicker hardness tester and analyze the hardness results. The second approach is to examine the raw material composition using an expensive biological microscope with three eyes, an LED Terino 300TC (40X-2000X, with a 5.1 MP high-resolution industrial camera) - Genuine items. To assess the depth size of the depth case, combine the outcomes of the two approaches mentioned above. The results will be skewed because of inaccuracies if the depth case analyst employs one of the aforementioned techniques.

The objective response functional evaluations are used to assess the optimization and design of the experiment. This work needs to optimize three functional parameters: the distance between the coil and the material surface, the coil travel time, and the supply voltage. The three product outputs following induction heat treatment, stiffness, penetration depth dimension, and tensile value, are assessed using the L9 standard orthogonal network. Taguchi calculated functional feedback values in experimental analysis using the formula translated into signal-to-noise (S/N) ratios (Eq. 32).

32 $$\:\frac{S}{N}=-10\text{log}\left(\frac{1}{n}\sum\:_{i=1}^{n}\frac{1}{{y}_{i}^{2}}\right)\:\:\:\:\:\:\:\:\:\:\:\:\:\:\:\:\:\:\:\:\:\:\:\:\:\:\:\:\:\:\:\:\:\:\:\:\:\:\:\:\:\:\:\:\:\:\:\:\:\:\:\:\:\:\:\:\:\:\:\:\:\:\:\:\:\:\:\:\:\:\:\:\:\:\:\:$$

Where n: number of experiments, $\:{y}_{i}$: value of characteristics.

A mathematical formula called analysis of variance (ANOVA) is used to assess the association between the parameters of each subject and ascertain the degree to which each parameter in the design interacts with the others. Design of an experimental test. The effects of surface-to-surface distance, travel time, and power supply voltage on the tensile strength, penetration depth, and hardness of the SUJ2 material were examined using ANOVA analysis—heat treatment by induction. The analysis results from the Taguchi approach are more precise since the ANOVA method shows how much attention is placed on the relative importance of each parameter’s effects. The researcher employed Minitab 18.0 software to conduct Taguchi and ANOVA analyses in this investigation.

Using Eqs. (33), (34), and (35), respectively, a confirmation test is run to confirm the accuracy of the output values for the parameters of stiffness, depth case, and tensile strength.

33 $$\:{HRC}_{opt}={m}_{h}+\left({V}_{2}-{m}_{h}\right)+\left({T}_{3}-{m}_{h}\right)+\left({d}_{3}-{m}_{h}\right)\:\:\:\:\:\:\:\:\:\:\:\:\:\:\:\:\:\:\:\:\:\:\:\:\:\:\:\:\:\:\:\:\:\:\:\:\:\:\:\:\:\:\:$$

34 $$\:{t}_{opt}={m}_{y}+\left({V}_{3}-{m}_{y}\right)+\left({T}_{2}-{m}_{y}\right)+\left({d}_{3}-{m}_{y}\right)\:\:\:\:\:\:\:\:\:\:\:\:\:\:\:\:\:\:\:\:\:\:\:\:\:\:\:\:\:\:\:\:\:\:\:\:\:$$

35 $$\:{TS}_{opt}={m}_{u}+\left({V}_{3}-{m}_{u}\right)+\left({T}_{3}-{m}_{u}\right)+\left({d}_{3}-{m}_{u}\right)\:\:\:\:\:\:\:\:\:\:\:\:\:\:\:\:\:\:\:\:\:\:\:\:\:\:\:\:\:\:\:\:$$

Where $\:{m}_{h},\:{m}_{y},\:{m}_{u}$: average of the experience test

The Confidence interval (CI) value is used to evaluate the optimal value of the Taguchi experimental study. Confidence interval (CI) values are calculated through two equations, (36) and (37), respectively.

36 $$\:CI=\sqrt{{F}_{\alpha\:,1,{f}_{e}}{V}_{e}\left[\frac{1}{{n}_{eff}}+\frac{1}{R}\right]}\:\:\:\:\:\:\:\:\:\:\:\:\:\:\:\:\:\:\:\:\:\:\:\:\:\:\:\:\:\:\:\:\:\:\:\:\:\:\:\:\:\:\:\:\:\:\:\:\:\:\:\:\:\:\:\:\:\:\:\:\:\:\:\:\:\:\:\:\:\:\:\:\:\:$$

37 $$\:{n}_{eff}=\frac{N}{1+{T}_{dof}}\:\:\:\:\:\:\:\:\:\:\:\:\:\:\:\:\:\:\:\:\:\:\:\:\:\:\:\:\:\:\:\:\:\:\:\:\:\:\:\:\:\:\:\:\:\:\:\:\:\:\:\:\:\:\:\:\:\:\:\:\:\:\:\:\:\:\:\:\:\:\:\:\:\:\:\:\:\:\:\:\:\:\:\:\:\:\:\:\:\:\:\:\:\:\:\:\:\:\:$$

Where $\:{F}_{\alpha\:,1,{f}_{e}}$: 95% confidence, $\:{V}_{e}$: variance error, $\:{n}_{eff}$: number of replications, $\:{T}_{dof}$: parameters degrees of freedom.

### Result and disscusion

The three output parameters, stiffness, depth case, and tensile dimensions, were computed, and the signal-to-noise ratio (S/N) coefficient was examined for the functional feedback parameters in the Taguchi experimental design (Shown in Table 19). Through the Taguchi experimental study, a response (Hardness) analysis is examined in detail and displayed in Table 20 with S/N indexes. Examine the input parameter influence correlation for the hardness output factor based on the experimental study’s input parameter interaction chart. Canbolat et al. (2019) state that the lowest S/N value indicates the least amount of input parameter influence. In other words, the S/N value yields the highest value, indicating that the amount of effect of the input parameter on the output parameter is ideal, and vice versa. The input parameters have a low level of influence on the output parameter hardness. The criterion states that the better the gold, or more precisely, the more optimal the parameter, the smaller the S/N value. The findings of the Taguchi analysis are that the coil travel time needs to reach 10 s, the amperage parameter needs to reach 90 A, and the coil needs to be 5 mm from the product surface. The findings indicate that the coil distance has a product surface size of 5 mm, the coil trip duration is 10 s, and the current value is 100 A. The Taguchi analysis results with the Depth case value impacted by three input parameters. The same analytical results for the tensile value, with the recorded values being the current value of 100 A, the coil moving time of 10 s, and the distance of 10 mm. Following induction heat treatment, the product is in its hardest and most stable state. Table 21 displays the statistical analysis using the ANOVA approach from Taguchi analysis to determine the contribution ratio of each input parameter to the product hardness. The best conditions for using the induction heat treatment machine for products made with SUJ2 material are as follows: the product’s surface distance (5 ± 0.5 (mm) from the coil, a current value of 95 ± 10% A, and a time of 10 ± 2 (2) for moving the coil. These results are based on the results of Taguchi and ANOVA analyses.

**Table 19:** Experimental results and their S/N ratios.

No.	Process parameter			Response (Target)			S/*N* ratio		
	A	T (s)	d (mm)	H (HRC)	t (mm)	TS (MPa)	H	t	TS
1	80	5	3	58	1.6	170.0	35.29	4.08	44.61
2	80	7	5	58	1.7	175.0	35.29	4.61	44.86
3	80	10	7	59	1.6	180.0	35.42	4.08	45.10
4	90	5	5	59	1.6	187.5	35.42	4.02	45.46
5	90	7	7	58	1.6	190.0	35.27	4.08	45.58
6	90	10	3	59	1.7	205.0	35.42	4.60	46.24
7	100	5	7	58	1.8	198.0	35.27	5.11	45.93
8	100	7	3	58	1.9	195.0	35.34	5.58	45.80
9	100	10	5	59	2.0	200.0	35.42	6.02	46.02

**Table 20:** Taguchi analysis: hardness, depth case, tensile versus A, T, d.

Level	Hardness versus A, T, d			Depth case (t) versus A, T, d			Tensile (TS) versus A, D, d		
	A	T (s)	d (mm)	A	T (s)	d (mm)	A	T (s)	d (mm)
1	58.33	58.33	58.50	1.633	1.667	1.733	175.0	185.2	190.0
2	58.67	58.17	58.67	1.633	1.733	1.767	194.2	186.7	187.5
3	58.50	59.00	58.33	1.900	1.767	1.667	197.7	195.0	189.3
Delta	0.33	0.83	0.33	0.27	0.10	0.10	22.7	9.8	2.5
Rank	2.5	1	2.5	1	2.5	2.5	1	2	3
$\:\sum\:delta$	1.49			0.47			35		
Weight	25.56%			57.44%			52.45%		

**Table 21:** ANOVA analysis: hardness (HRC) versus A, T, d.

Source	DF	Adj SS	Adj MS	F-Value	*P*-Value	*R*-sq	*P*. Stdev
Current	2	0.1667	0.0833	0.27	0.770	8,33	0.552771
Time	2	1.1667	0.5833	4.20	0.072	58.33	0.372678
Distance	2	0.1667	0.0833	0.27	0.770	8.33	0.552771
Error	6	1.8333	0.30556				
Total	8	2.0000					

The ideal induction heat treatment machine’s machining conditions are determined by applying the Taguchi optimization method and One Way ANOVA analysis to experimental research at the induction heat treatment process (electric current is 95 ± 10% (A), coil travel time is 10 ± 2 (s), and coil distance from suj2 material is 5 ± 0.5 (mm).

**Fig. 22:**
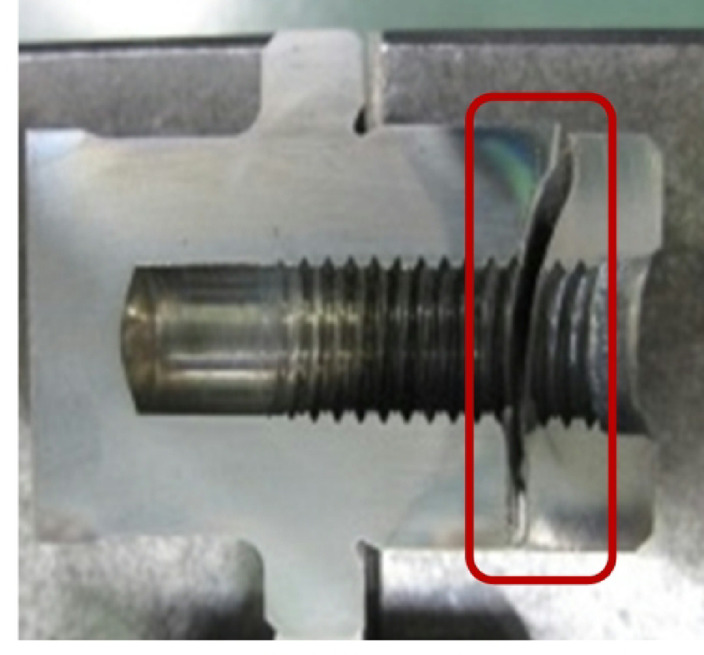
Crack’s defect.

The electric current enters the coil directly and produces heat, which heats the SUJ-2 material’s surface, modifies the material’s physical characteristics, and modifies the carbon element’s physical characteristics on the SUJ-2 material body. If the power supply’s quality is not guaranteed by electric current, it may result in faulty goods such as cracks (Fig. 22. Crack’s defect), unsatisfactory material structural compositions, and other mistakes. External elements like thunder and other electromagnetic disturbances directly impact power quality. Furthermore, a variety of high-frequency switching devices and internet-of-things (IoT) gadgets, including LED lights, inverters, switching sensors, and high-frequency switching switches, are utilized in industrial facilities. Large quantities. They cause the current to experience harmonic interference. Regulating the THD value within the IEEE 519:2022 standard is urgently necessary to improve power quality.

**Fig. 23:**
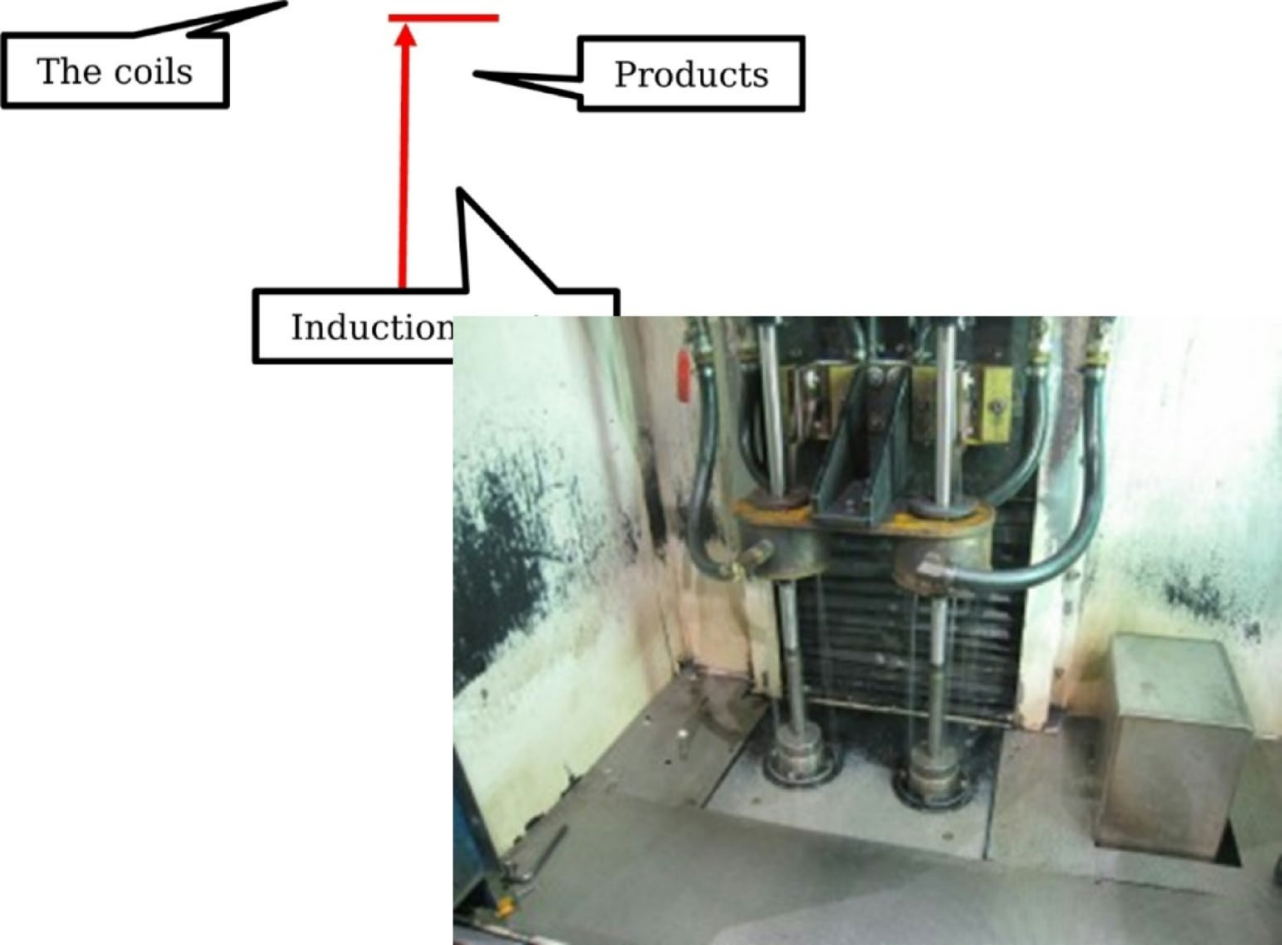
Induction heat treatment process.

Since stainless steel is used to make coils, they will eventually age and bend like lightning. If the coil’s physical composition fails to satisfy quality criteria, the electrical current suffers and wastes a lot of energy. The coil is directly coupled to the electronic current wire and is changed by the diameter of each type of product (Shown in Fig. 23). Coil replacement and monitoring are critical tasks. Establishing a coil life assessment and developing a coil maintenance program with the Industry 4.0 system are necessary steps in enhancing quality control. Procedure and quality requirements for induction heat treatment. Ten instances of employees selecting the incorrect induction heat processing program and calling the incorrect program were found in evaluating the quality history of the induction heat process between May 2020 and May 2021. The coil will collide with the product body during the induction heat treatment process, ironing, breaking, and bending the coil, in addition to creating labor issues. To satisfy replacement operations, the author suggests developing a digital numerical control system that calls for induction heat processing programs automatically. This will be accomplished by combining an RFID system, IOT technology, and a SQL database management system. The worker must minimize collision events between the coil and the product’s material body and ask for the induction heat treatment program.

The improvement team is developing an RFID system with a tag search feature, a mechanism to send data from an RFID tag to a reader, and an SQL database system for data gathering. All of a company’s databases are linked via the RFID system, which makes user operation simple. By connecting and transmitting data from cells at every stage of production, the system creates an information layer akin to the Internet of Things. Based on customer needs, the Cloud-MES layer stores and categorizes different sorts of data. Users’ privacy is the priority for RFID system operators, but there are also big benefits that lead to effective corporate operations. Smartphone data is gathered by the RFID device and stored in a cloud database system. Users can make well-informed judgments and enhance business development with the use of this database’s statistical charts and understandable analysis.

### Design digital numerical control system via RFID system

**Fig. 24:**
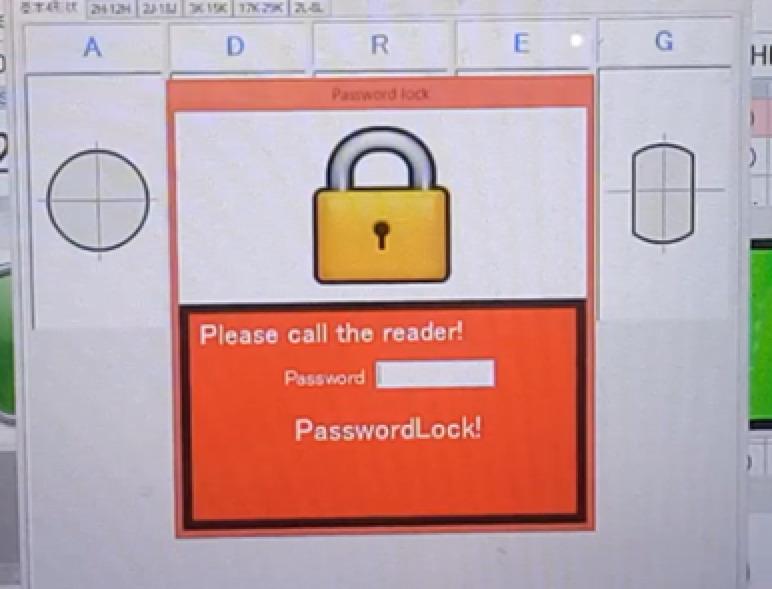
Warning screen.

Create a master list of induction heat treatment schedules appropriate for each kind of product line and store it in a file with the CSV file format. Connect to the production system on the organization’s cloud network system and upload the master list of this program file into the SQL database system. Using C# software, create a program that calls each induction heat treatment program from the RFID system. The program call will react correctly, and the system will automatically call the induction heat program when an employee uses a barcode reader to scan the RFID code and sends a request to contact the corresponding processing program for the product code that requires induction heat treatment. Without relying on the workers’ understanding, comprehensive information about the machining program and its treatment should be provided on the panel screen. Suppose the SQL system receives a program call from the RFID tag without a program code for induction heat treatment. In that case, the system will raise an alert, display a warning screen on the panel screen, and disable the RFID system. The induction heat treatment equipment is shut off and locked (Shown in Fig. 24).

By integrating the RFID technology with the digital numerical control (DNC) system, staff members can choose the number of induction heat treatment programs without having to use their index fingers on the panel screen. This eliminates the possibility of selecting the incorrect program and enhances the quality of the program. Save the machine tool quality and avoid wasting time by choosing the same programs repeatedly because your finger accidentally touched a different program number. Increased coil longevity and quality equate to less wasted power and electric current. Calculations show that an electric shock is produced every time the coil and the product’s metal body collide. This electric shock overloads the CB at the source, cutting off power to the entire factory and doing all the work. Processing equipment, including 355 additional units, must stop abruptly. This results in damaged goods, broken tools, and time-consuming machine restarts. It also causes workers to be absent, among other cost-related issues. It is more than USD 5,000 for each instance where the source of the CB power is cut off. Utilizing RFID technology and automation in business and production is crucial to eliminating tasks that rely on employee skills. The inexpensive Arduino board serves as a control board for communicating DNC and RFID system functionalities.

The coil produces lightning, which causes numerous holes and fissures to appear on its surface. These places experience a loss of current sources, raising the price of power production. According to the company’s current maintenance schedule, technicians clean and maintain coils using a paper plan, visually check the plan regularly, and conduct coil inspections. The coil will not be detected if the maintenance staff follows the maintenance plan and checks and cleans the coil at random. Likewise, if the electrical engineer reviews the maintenance plan and signs it to attest to its accuracy but does not carry out any maintenance, the system will not detect it. When employing paper designs, the maintenance engineer’s skills are the only factor that matters. To end industry and coil maintenance’s reliance on people, 4.0 system upkeep is needed to enhance industry and coil quality, and 4.0 system quality is required in order to cut expenses and electrical energy use. The author suggests utilizing Industry to create a maintenance system that accounts for production expenses regarding electrical energy use. 4.0 framework.

The maintenance plan file is imported into the maintenance system master on the SQL database system, and the maintenance plan master list is established on the CSV file. The program calling system is connected to the maintenance plan master data—thermal treatment by induction. The induction heat treatment machine shuts off, and the Industry 4.0 system displays a warning screen on the screen panel when the coil and system maintenance are about to expire. After doing maintenance on the coil, the maintenance team uses a barcode scanner to scan the RFID tag on the coil. This allows the RFID system to communicate with the SQL system, indicating that the coil has been maintained. The induction heat treatment will remain off if the system detects no RFID signal on the coil. The Industry 4.0 system establishes a corresponding time for the coil maintenance cycle to control the coil maintenance time. If the system needs maintenance between the times it takes for the maintenance cycle of one coil and when it needs maintenance, it will not receive information about the maintenance results.

After implementing the Active Power Filter (SAPF) in the induction heating system, the power quality indicators, energy efficiency, and defective product rate all improved significantly. Before applying SAPF, the total harmonic distortion (THDi) reached 36.35%, exceeding the allowable level according to IEEE 519:2022, leading to large temperature fluctuations during the heating process, up to ± 50 °C, reducing product uniformity. After SAPF was implemented, THDi decreased to 5.21%, equivalent to an improvement of 85.67%, helping to stabilize the power supply and reduce voltage noise. At the same time, the power factor (Cos Φ) increased from 0.89 to 0.98, an increase of 10.11%, demonstrating the effectiveness of SAPF in optimizing energy use and reducing reactive power loss. The stability of the current also contributes to more precise control of the heating temperature, reducing the temperature fluctuation from 50 °C to 5 °C, corresponding to a 90% improvement, ensuring a more uniform heat treatment process. Product quality also changed significantly after integrating SAPF into the system. Before the improvement, the defective rate of products was very high, up to 90%, mainly due to the instability of the current, causing uneven heat distribution in the material. After SAPF was applied, the defective rate dropped sharply to only 1%, an improvement of up to 98.89%, proving that power quality control directly impacts the performance of induction heat treatment. In addition to improving product quality, SAPF also brings benefits in energy efficiency. Before the improvement, the system consumed an average power of 55 kW, but after optimization with SAPF, the consumption was reduced to 48 kW, resulting in 12.73% energy savings. At the same time, the system’s energy efficiency also increased from 75 to 85%, equivalent to an improvement of 13.33%, indicating that the system operates more efficiently, reduces energy waste, and increases sustainable operation. The above results not only demonstrate the important role of SAPF in improving power quality and optimizing energy efficiency but also demonstrate its direct impact on heat treatment quality and qualified product rate. The reduction of voltage noise and increased current stability have helped control the sintering temperature better, significantly improving the uniformity of the processed material, reducing energy consumption, and improving production efficiency. This demonstrates the effectiveness of the SAPF model in optimizing industrial production processes, especially in systems requiring high temperature and energy precision, such as induction heating.

### Comparison and validation

To evaluate the effectiveness of the SAPF + Six Sigma model, we compared it with existing methods and performed validation through experimental data, statistical testing, and cost-benefit analysis. First of all, when compared with traditional power quality improvement methods, the SAPF + Six Sigma model shows many outstanding advantages. Compared with passive harmonic filters, this model is more effective in reducing harmonics while integrating real-time monitoring to maintain stable power quality. When placed next to artificial neural network (ANN)-based control systems, SAPF + Six Sigma has a lower level of complexity but still ensures significant harmonic reduction at a more reasonable cost.

To validate the model, we conducted a power quality evaluation before and after SAPF implementation based on the IEEE 519:2022 standard. Experimental results showed that THDi decreased from 36.35 to 5.21%, ensuring meeting the allowable standard of less than 12%, while THDv decreased from 4.31 to 1.89%, significantly improving voltage stability. The power factor was also raised from 0.89 to 0.98, helping to increase power efficiency. In addition, the production defect rate analysis showed a clear change when the defective product rate decreased from 9.8 to 1.1%, while the deviation of surface hardness and hardening permeability were reduced by more than 85%, significantly improving product quality. Next, we used the PLS-SEM method to test the model’s reliability. The results show that Cronbach’s Alpha is 0.91 and Composite Reliability is 0.92, indicating the model has high reliability. Moreover, the R² coefficient is 0.82, indicating that the model has a good ability to explain the impact of SAPF on power quality. Finally, the cost-benefit analysis also shows the obvious effectiveness of the model implementation. Specifically, the annual energy cost is reduced by 20%, the loss due to machine downtime is reduced by 71.4%, and the total cost savings are up to 45.9%, helping to improve the profitability and operating efficiency of the enterprise.

Synthesizing the above results, it can be concluded that the SAPF + Six Sigma model not only helps to improve power quality significantly but also improves production efficiency, reduces product defect rate, and brings obvious economic benefits. With wide application potential, this model can be expanded by integrating AI and IoT for real-time automatic optimization, helping to maintain stable power quality and improve production efficiency in the Industry 4.0 era.

### Sensitivity analysis and model validation

Sensitivity analysis plays an important role in assessing the impact of input variables on the output results of the Six Sigma model combined with SAPF. In this study, we applied two methods: One-At-a-Time (OAT) and Comparative Sensitivity Analysis Method (COMSAM) to analyze the influence of factors on the total harmonic distortion THDi and the material hardening depth during induction heat treatment. The results show that for THDi, the supply current has the largest influence, with a fluctuation of 8.3%, while the supply voltage and load power also have an impact but at a lower level (6.1% and 6.2%). Meanwhile, when analyzing the sensitivity of the material hardening depth, the heating time has the highest influence (9.1%), followed by the current intensity (8.9%) and the coil spacing (5.8%). This shows that good control of the heating time and the supplied current is the key factor in optimizing the heat treatment quality. In addition, to validate the model, we compared the predicted and experimental results. The results showed that the error between the actual and predicted model values was very low, with a THDi error of only 1.18%, a hardening depth error of 2.38%, and a power factor (Cos φ) error of 0.51%. This proves that the Six Sigma + SAPF model has high accuracy and can be effectively applied in practice. Overall, the sensitivity analysis shows that the current intensity and heating time are the two most important factors determining the power quality and material hardness. To further enhance system performance, future research could integrate artificial intelligence (AI) and IoT to optimize real-time power control, helping to ensure power stability and minimize errors in the manufacturing process.

## Discussion

All managers prioritize controlling production and business expenses in the operations of manufacturing firms and service providers in general. The costs incurred during production and business operations determine a company’s profitability. Sales earnings less input production costs are how profit is computed. A situation where the manufacturing cost exceeds the profit sold indicates that the business corporation is losing money and does not include a single individual. If the production cost is excessively high, the profit will be low—anything the management wants. Cutting production costs is always the most important and pressing problem for firm managers. Almost all industrial and business enterprises focus on and implement continuous improvement operations utilizing the Six Sigma method to increase business efficiency.

Machine costs, material costs, labor costs, sintering costs, de-binding costs, consumable expenses, and other expenditures are all included in production costs. Improvement projects need a lot of time and effort to complete. Managers need to have a plan in place for improving the problem’s focus and choosing improvement topics to carry out continuous improvement activities successfully. Increasing productivity is a pressing topic for business managers. The Fuzzy TOPSIS approach is a useful tool for choosing the focal improvement problem in a variety of industrial and business problem domains. A process that offers precise standards for thoroughly assessing the focal points and evaluating every major issue point, assisting managers in identifying problem areas based on the rankings, and putting adjustments into place. Utilize Industry 4.0 tools and the Six Sigma methodology to automate processes and solve problems in a comprehensive and highly efficient manner.

The fuzzy TOPSIS approach was used in this study to identify issues with picking improved electrical energy sources for the induction heat treatment process, as well as with monitoring the consumption of the highest-ranking electrical energy sources. The induction heat treatment process’s electrical energy consumption can be attributed to various factors, including electromagnetic interference (EMC) in the power supply, harmonic signal-induced power quality degradation, and low-quality coil-induced power consumption loss. Amount (bent, clay). To minimize power loss, raise power quality, eliminate interference, and enhance coil maintenance procedures to find issues with the coils.

**Fig. 25:**
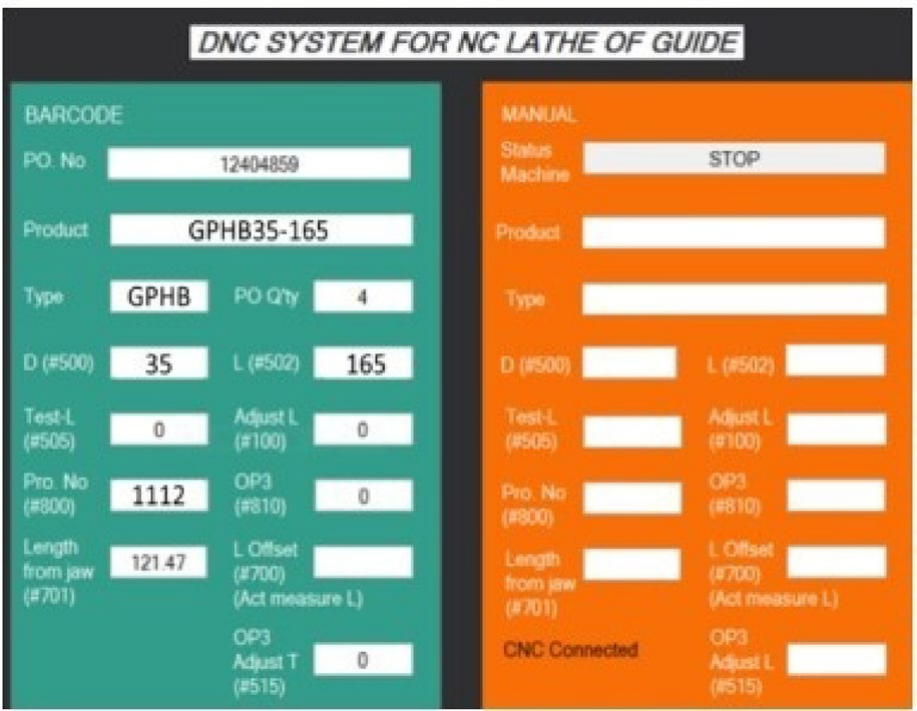
DNC screen.

**Fig. 26:**
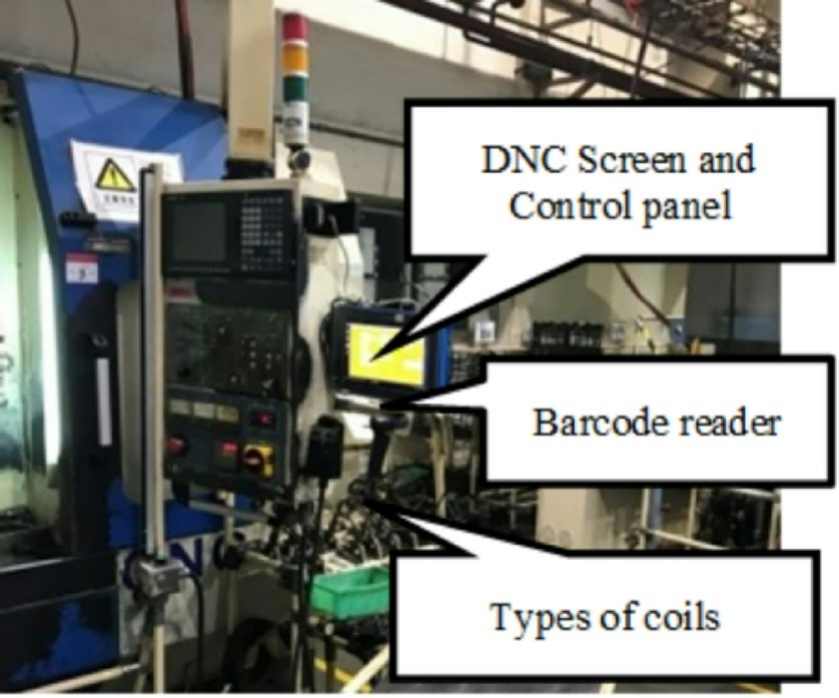
Actual DNC at the Induction heat treatment machine.

The development of affordable IOT devices is a prerequisite for the advancement of Industry 4.0 technology and presents an opportunity to create comprehensive Six Sigma-based improvement solutions. This study suggests using digital numerical control (DNC) technology to regulate the machining system, RFID technology to construct an autonomous induction heat treatment program, and an Industry 4.0 system to enhance and control. The proficiency of the individual operating the induction heat treatment machine determines the removal maintenance system (Shown in Fig. 25). Simple yet efficient procedures are carried out by the DNC system, saving the machine operator from repetitious or error-prone tasks (Shown in Fig. 26). The DNC system enhances operator satisfaction with Induction heat treatment machines. Enhance the productivity of the manufacturing process, cut down on production time, raise the quality of the final product and the production process, lower production costs, and lessen tool wear and damage. Increasing the lifespan of machine tools has the benefit of enhancing the company’s competitive edge in the manufacturing and service sectors.

The Industry 4.0 application improvement system is also assessed as a technological product. Industry 4.0 system uses IoT devices and software to link and call programs in the DNC system^42^. The operators of this Industry 4.0 system are the technicians, maintenance technicians, and induction heat treatment machine operators. The technology products of this system are immediately implemented in the induction heat treatment process. The company’s management and production technicians^43,44^. These users and operators of Industry 4.0 have varied levels of experience with induction heat treatment equipment. Utilizing the Industry 4.0 system increases the efficacy and efficiency of the continuous improvement system. Additionally, look into user satisfaction with the system to accomplish goals and gather information for future project enhancements^43^. A survey of industrial users of Industry 4.0 systems is conducted using the PLS-SEM approach^45^. User contentment with the industry’s usage of improvement systems and 4.0 technology in Six Sigma-based continuous improvement initiatives. Assess user satisfaction with industry outcomes. Using the Technology Acceptance Model (TAM) theory, 4.0 technology system application enhancement activities are carried out based on perceived usefulness and ease of use^46–48^. Industry 4.0 technology measures information model-related parameters such as information system quality by utilizing RFID systems, application software, Internet of Things devices, and database systems. When it comes to continuing to use and operate the system with Industry 4.0 technology, happy users’ top concerns are the quality of information services and the quality of user information^49,50^.

Perceived utility and perceived ease of use are two elements that affect users’ acceptance of a technology system. Perceived usefulness is the performance improvement that is thought to have occurred. In contrast, perceived ease of use is the ease of use that is impacted by the behavioral intentions of the user.

Wi-Fi connectivity and digital control are integrated with Industry 4.0 technologies to provide real-time electricity consumption monitoring. The information technology network depends on quality assurance to maintain data transfer between smart devices and an unbroken internet connection^51^. However, implementing these technologies in Vietnam is difficult due to its poor infrastructure and antiquated IT systems. User happiness depends critically on the quality of information transfer between smart devices, especially regarding power usage statistics. It is recommended that suppliers prioritize the quality of their services, systems, and information to enhance user intentions and happiness with Industry 4.0 systems^52,53^. Three primary aspects impact the technology model, especially in the context of Industry 4.0: information quality, system quality, and service quality. The degree to which consumers intend to use digital technology consistently is directly impacted by its quality. This study advances our understanding of the digital technology knowledge-sharing platform. Thus, maintaining the quality of the information is essential to user happiness and the technology model’s effective deployment. Technology quality impacts service quality, and providers use customer appreciation programs to guarantee customer happiness. User intentions are positively impacted by high-quality information and systems, underscoring the significance of quality in information technology.

### Hypothesis 1

*(H1)*: The level of service the information technology model provides directly influences users’ intentions to use it continually.

### Hypothesis 2

*(H2)*: The user’s intention to use continually is directly impacted by the information technology model’s system quality.

### Hypothesis 3

*(H3)*: An information technology system’s information quality directly impacts the user’s intention to use it continually.

The industry’s acceptance of technology is impacted by several elements, including innovation, insecurity, and discomfort. Consumers view technology favorably and consider it to be helpful. Negative factors, such as ignorance of the advantages and simplicity of use, could prevent Industry 4.0 technology from being adopted. Users may regulate real-time power usage with the Industry 4.0 system, which reduces expenses and improves operational procedures. This cutting-edge system controls smart, power-hungry devices using speech and Wi-Fi. The personal information of the users is used by smart manufacturing systems to access and operate Industry 4.0 technologies. However, hackers may access and take this data, which could have unfavorable effects—the chief of smart manufacturing worries about the items’ digital control technologies’ clarity and security. The Industry 4.0 system is linked to this problematic user material. The technological model of the digital control product consists of four main parts. (1) Optimization: New technology increases life’s control, improves the enjoyment of activities, and makes life more adaptable and effective. (2) Innovation: New technology increases the adaptability of products with digital controls by bringing new viewpoints and possible uses. (3) Insecurities: The technology’s intended use does not meet every user’s specific needs. Swaying user opinions against a product that is digitally managed. (4) Uncomfortable: The inability of digital control technology to operate smoothly causes users to feel uncomfortable. The Technology Acceptance Model (TAM) idea emphasizes the influence of both favorable and unfavorable aspects on users’ propensity to use Industry 4.0 frameworks. The theory emphasizes the significance of maximizing energy usage and minimizing waste by arguing that customers’ expectations about digitally controlled items directly influence their intention to use them constantly. According to the hypothesis, consumers are ready to embrace technology such as the Industry 4.0 system, which lowers costs and boosts productivity, and the information technology model meets users’ expectations.

### Hypothesis 4

*(H4)*: The optimization factor influences the theory of technology acceptance favorably and indirectly influences the desire of the user to use it continually.

### Hypothesis 5

*(H5)*: The hypothesis of technological acceptance is directly impacted by the innovation factor, and users’ intentions to continue using the technology are indirectly affected.

### Hypothesis 6

*(H6)*: The hypothesis of technology adoption is directly impacted by insecurity considerations, and users’ intentions to use continually are indirectly impacted.

### Hypothesis 7

*(H7)*: The main factor indirectly impacts the notion of technology acceptability and users’ intention to use continually.

### Hypothesis 8

*(H8)*: The perceived usability factor is directly influenced by the perceived ease of use factor, and the user’s intention to use continually is indirectly influenced by this component.

### Hypothesis 9

*(H9)*: The expectation confirmation factor, which has an impact on both the perceived utility factor and the user’s intention to use continually, is a key component of the theory of expectations.

### Hypothesis 10

*(H10)*: Perceived usefulness directly affects users’ inclination to use something repeatedly.

Data for the empirical study analyzing the happiness of Industry 4.0 system users was gathered from ABC Company, which has its head office in Japan and a production branch in Vietnam. To ensure objectivity and representativeness in the survey results, the research team employed random methods to collect data for the survey. After being enhanced at the induction heat treatment process and having more than a year of experience operating the induction heat treatment machine, the respondents to the survey are those who directly use and operate the Industry 4.0 system. The respondents to the poll, who work directly in production and improvement tasks, believe that enhancing the use of Industry 4.0 technologies will provide positive outcomes. The research questionnaire was completed by the continuous improvement project. It was created based on reference standards from reliable scientific studies about the real-world monitoring and functioning of the industry system, as well as the implementation of Industry 4.0 technology. 4.0 in the smart factory’s production process. There are three or more measurement variables for each research variable. The questionnaire is constructed in English and translated into Vietnamese to create an additional table. The items are rated on a Likert-5 scale. The questionnaire was changed in response to five academics’ and experts’ ideas and opinions regarding the use of Industry 4.0 in manufacturing.

Three consecutive shifts of eight hours each are used to operate the induction heat treatment procedure. Twenty direct operators, one leader, two sub-leaders, three quality control employees, two maintenance employees, one product technician, and eleven machine operators make up each shift. A total of sixty individuals work three shifts. Experts in implementing and managing Industry 4.0 systems in production are also present, including one production manager, one technical manager, and one quality assurance manager. The poll was put together and released. 63 surveys were completed, and 63 responses were received. Following the appropriateness check, 58 survey panels satisfied the requirements and were permitted to proceed with the analysis of the subsequent steps, whereas 5 survey tables had data that did not match the sampling requirements (Shown in Table 22). The five surveys that don’t match the requirements could be because the surveyor didn’t thoroughly read the questionnaire, didn’t have time to complete it due to work obligations, or used a modern evaluation instead of conducting the survey. I agree with the survey’s question content, yet the surveyor still doesn’t get it.

**Table 22:** Sample characteristics.

Variable	Items	Freq.	Per.
Gender	Male	50	86.2%
	Female	8	13.8%
Age	20–30	50	86.2%
	30–40	10	17.2%
	Over 40	3	5.2%
Academic	Upper university	3	5.2%
	University	10	17.2%
	College	5	8.6%
	High school	45	77.6%

**Fig. 27:**
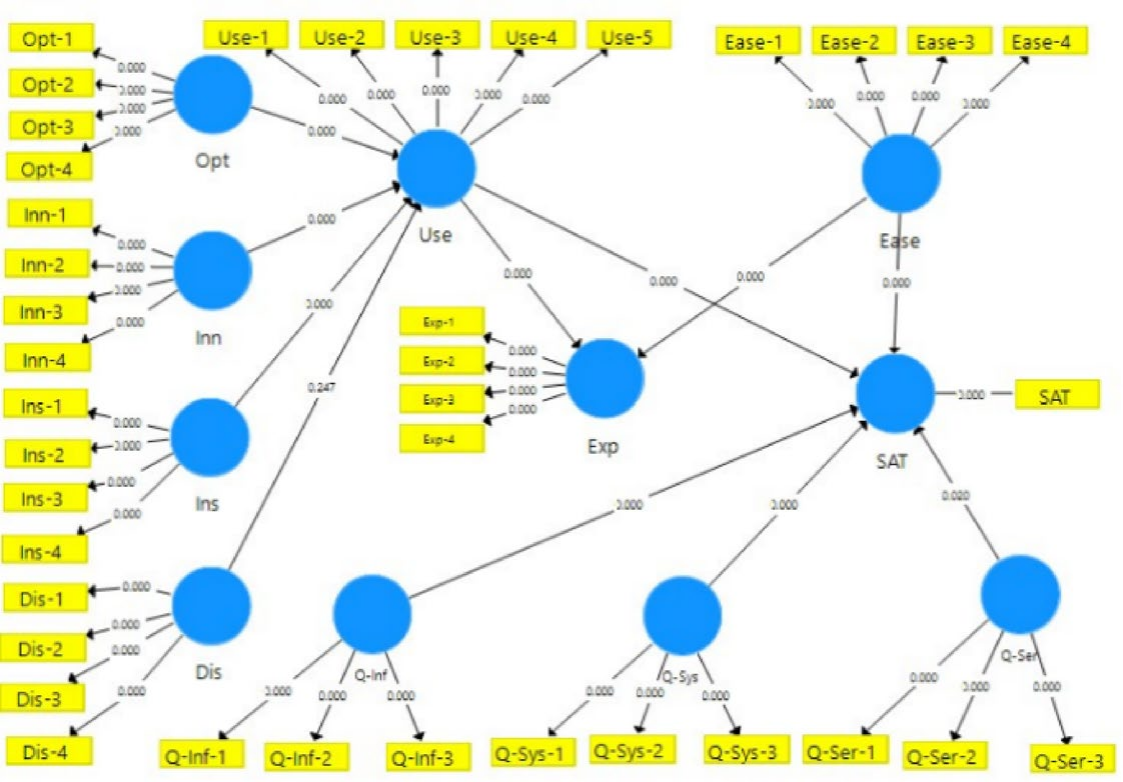
Analysis PLS-SEM Model.

The survey model was analyzed using smartPLS 3.3.0 software by entering 58 survey results. With the requirement that the CR value must be more than 0.8, the path model analysis findings compute the Composite reliability (CR) parameters (Eq. 40) to assess the scale value’s dependability. Assessing the dependability of measurement factors involves calculating the Cronbach’s Alpha value (Eq. 37), requiring the value of the Cronbach’s Alpha to be greater than 0.6. Utilizing the criterion that the AVE value must be larger than 0.5, compute the AVE value (Eq. 38) used to assess the research model’s dependability. All CR parameters are larger than 0.8, all Cronbach’s Alpha values are greater than 0.6, and all model AVE values are greater than 0.5, according to Table 23’s model analysis results (Shown in Fig. 27). This demonstrates that the data can be utilized for additional study model analysis.

**Table 23:** Convergent validity and reliability.

Const.	Ind.	Factor	CR	AVE	Cronb.
Tech.	Opt.	0.732	0.821	0.656	0.876
	Inn.	0.801			
	Ins.	0.810			
	Dis.	0.789			
TAM	Use.	0.817	0.809	0.688	0.768
	Ease	0.795			
	Exp.	0.870			
Inf	Q-inf	0.799	0.904	0.765	0.799
	Q-sys	0.803			
	Q-ser	0.809			
Iten	Int	0.870	0.877	0.789	0.843

**Table 24:** Result of hypothesis analysis.

Hypo.	Path	Est.	T-Val.	S. E	*P*-Val.	Rest.
H1	Opt.	0.393	4.94	0.067	0.010	Pass
H2	Inn.	0.493	3.96	0.039	0.011	Pass
H3	Ins.	0.395	5.83	0.049	0.000	Pass
H4	Dis.	0.483	4.94	0.034	0.001	Pass
H5	Use.	0.494	4.97	0.058	0.013	Pass
H6	Ease	0.593	3.96	0.031	0.020	Pass
H7	Exp.	0.483	5.83	0.076	0.010	Pass
H8	Q-inf	0.594	5.13	0.032	0.013	Pass
H9	Q-sys	0.492	5.24	0.046	0.014	Pass
H10	Q-ser	0.393	6.81	0.068	0.021	Pass
H11	Int	0.592	4.14	0.062	0.014	Pass

Table 24 displays comprehensive findings from the study model’s structural model analysis. P-values for every measured variable are less than 0.05. This demonstrates the satisfaction of both staff members and respondents with the use of Industry 4.0 technology in the implementation of enhanced models for the induction heat treatment process. The analysis’s findings have provided the continuous improvement research team with the drive and knowledge to keep implementing comparable changes to other business procedures to lower production costs and boost output. By implementing Industry 4.0 technologies, production operations can increase efficiency, decrease errors, and boost employee happiness. According to the technical readiness measurement variables, a PLS-SEM approach is suggested to be deployed during the Control phase of the Six Sigma method to gauge user satisfaction with the upgraded system during the high-rise thermal quenching process. Approaches, information engineering measurement components, and evaluating the utility and usefulness of enhanced systems.

The employee satisfaction survey results before and after the Active Power Filter (SAPF) implementation showed significant improvements in many aspects related to the working environment, work performance, and system stability. Before the implementation of SAPF, the average employee satisfaction with work was only 3.2 points on a 5-point scale, while after the implementation, this increased to 4.5 points, corresponding to an improvement of 40.63%. Employee performance also changed positively as the job performance rating increased from 3.5 to 4.7, showing an improvement of 34.29%, thanks to the stability of the power system and the reduction of interruptions during operation. Notably, the level of awareness of power quality among employees increased sharply from 2.8 to 4.6, an improvement of 64.29%, thanks to the reduction of harmonics and voltage stability, which helped equipment operate more efficiently without causing unexpected errors. In addition, the level of employee stress due to technical problems during the production process also improved significantly, with the stress reduction index increasing from 3.0 to 4.4, equivalent to an improvement of 46.67%, showing that SAPF helped reduce voltage instability, limit system interruptions, and reduce pressure on operating staff. Most importantly, the awareness of production process stability increased from 3.1 to 4.8, an improvement of 54.84%, thanks to ensuring continuous power supply, minimizing heat treatment errors, and limiting abnormal technical problems. These results not only show the positive impact of SAPF in improving power quality but also help improve the working environment, reduce pressure on employees, increase labor productivity, and improve the stability of the production process. The application of SAPF not only brings technical benefits but also has a positive impact on the human factor in the enterprise, helping to improve the overall quality of labor and production efficiency in the modern industrial environment.

All factors are met, according to the measurements and analysis of the aim to employ the enhanced system in the high-rise thermal quenching process. Prove that the Hybrid Six Sigma invention satisfies the necessary criteria. Expert and process operator interviews provide a source of real data that feeds into the MADM technique, allowing it to examine and prioritize issues that require improvement fairly and sensibly. Effective at using the MDAM approach to analyze real-world problems in various manufacturing processes throughout the Six Sigma method’s analysis phase. The Taguchi approach is used to fairly standardize the parameter selection for the conditions in the high-rise thermal quenching process. The outcomes of this condition are then applied to the high-layer tempering process to ensure stability, assessed under thermal conditions, and guaranteeing that the product meets customer specifications for post-heat permeability size, which ranges from 1.8 mm to 2.2 mm. The Taguchi approach yields great business efficiency and is suggested for use in the enhancement phase of Six Sigma. It responds well to the optimization of machining conditions in the production process.

**Fig. 28:**
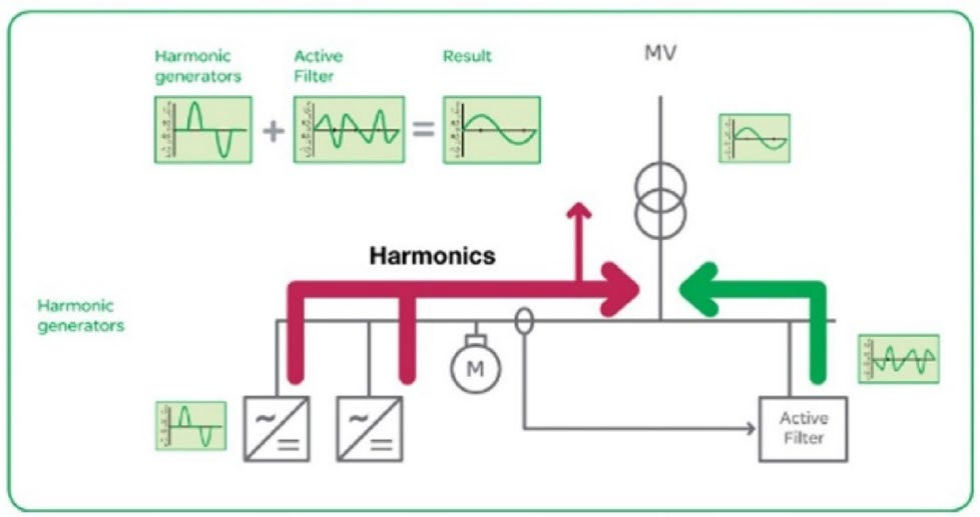
Recommended to install an Active Harmonic Filter.

To reduce the harmonics in the system, it’s recommended to install an active harmonic filter (Shown in Fig. 28). Devices for measuring load current, calculating deviations from user-specified objectives, and injecting the appropriate amount of current to achieve supply current objective levels for harmonics, displacement PF, or load balancing are all part of the electronic power quality suite. The logic determines the harmonic current spectrum, that is, the amplitude and phase angle for each harmonic up to the 50th order, after measuring the load current when harmonic mitigation is necessary. The amplitude to be injected at the opposite phase angle for each harmonic order chosen for mitigation is then determined by logic. The semiconductors (IGBT) are then instructed to replicate the control signal by injecting current into the supply once a control signal has been established. The supply-side harmonic current is significantly decreased in this way.

**Fig. 29:**
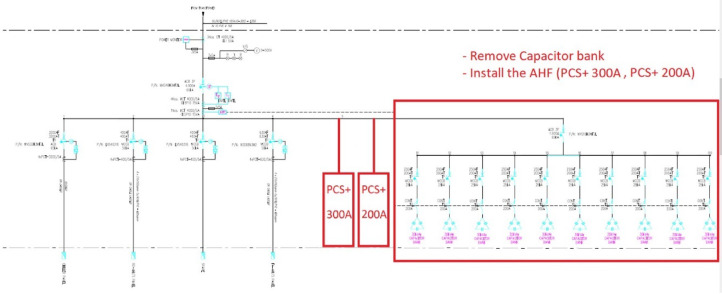
A detailed connection diagram accuSine PCS+, will be connected to the existing MSB switchboard.

Provide Active Harmonic Filter with Characteristics Active Harmonic Filter AccuSine PCS + 300A, including Encloser IP31, Main incoming MCCB 630A, AccuSine harmonic filter integrated 300A, Ventilation Fan, USB communication port, Ethernet Cable 5m, Display: HMI 5.7’’ touch screen with detail information about current, voltage, THDi, THDv, event logs, and Manufacture: Schneider Electric (Shown in Fig. 29).

**Fig. 30:**
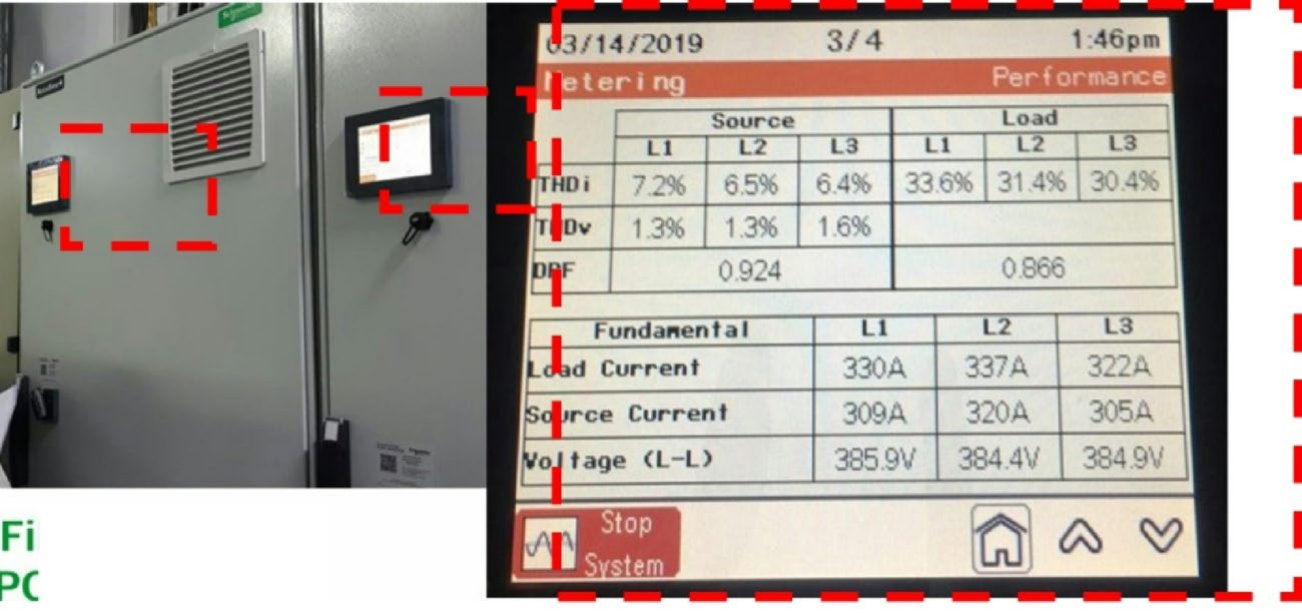
Metering of harmonic active filter.

The benefits of installing a harmonic filter are that the system meets IEEE 519: 2022 standards and meets the standards according to Circular 39/2015 regulations of the Ministry of Industry and Trade. Besides, the harmonic filter solution will help the customer improve system stability and performance (energy management) by reducing current spikes on the system, reducing distortion caused by harmonics, and eliminating the effects of harmonics on the system. Minimize the impact on equipment operating life (Asset Management) by reducing heat generation caused by harmonics, eliminating the risk of protective devices mis-tripping, and increasing the longevity and life cycle of devices. Optimize costs (Financial Management) such as power factor, always ensuring compliance with electricity industry regulations, and saving energy. Based on the measurement results of current, harmonics, and power factor values ​​when the filter operates. The harmonic values ​​of current THDi and voltage harmonics THDv not only meet the regulations of Circular 39/2015 BCT, IEEE 519:2022, but also meet the plant’s requirements, THDi ≤ 8% and THDv ≤ 4% (Shown in Fig. 30).

**Fig. 31:**
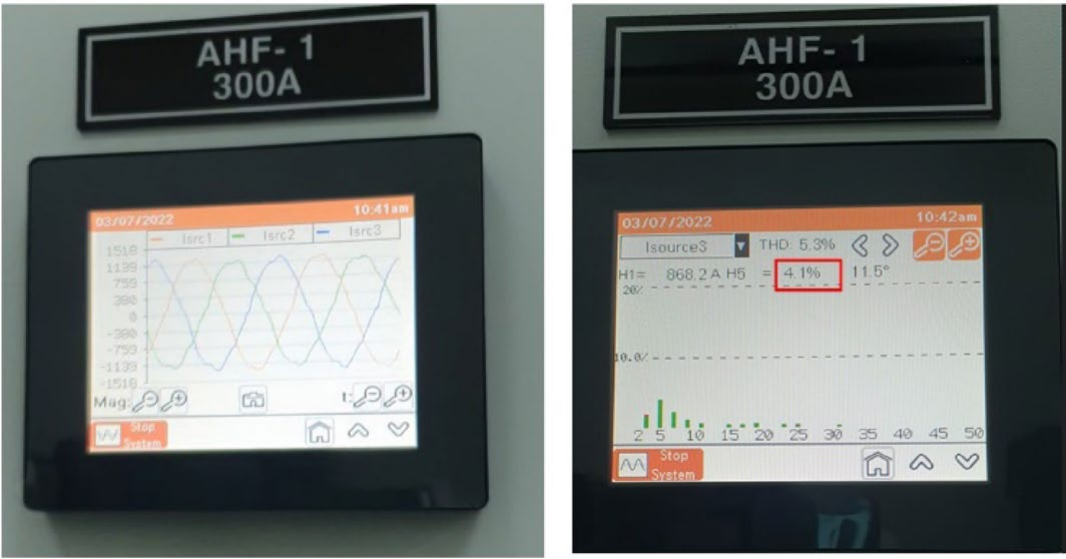
Actual THD real-time measurement.

The online measurement system saves the measurement data. The automatic maintenance system sets up the power quality measurement at the source and at the start of each production shift. The measurement value of power quality is the THD value within the 5% standard that complies with the IEEE 519:2022 standard (Shown in Fig. 31). Using RFID technology and the DNC method, the online measurement system is directly connected to the Industry 4.0 system, allowing data to be collected at the source and saved on the company’s common online measurement system. Production procedures integrate all phases like an Industry 4.0 system, assisting in the control of processing conditions and product quality.

The study applied the Six Sigma method combined with Active Power Filter (SAPF) to improve production quality in induction heating. Before implementing the improvement, the production process had problems with temperature and firing time control accuracy, resulting in 90% of the products having hardness defects, mainly due to unstable fluctuations in the current and unoptimized parameters. Applying the DMAIC (Define, Measure, Analyze, Improve, Control) method in Six Sigma helped identify important factors affecting hardness and make specific adjustments. In the Measure phase, data were collected from power quality monitoring sensors and hardness measurement systems, showing large fluctuations in the firing temperature and the supplied current. The Analyze step used DOE (Design of Experiments) analysis to determine that the most important factors affecting hardness were: heating time, current intensity, and distance between the induction coil and the material. Next, the Improve step made optimal adjustments by tightly controlling the heating time between 6.8 and 7.2 s, maintaining the current intensity at 90–95 A, and keeping the coil distance stable at 5 mm. At the same time, SAPF was implemented to reduce current fluctuations and ensure stability during the heating process. Experimental data after implementing the improvement showed that the defective product rate was reduced from 90% to only 1%, thanks to maintaining the precision in the heat treatment process. The stability of the sintering temperature has been significantly improved, reducing the fluctuation from ± 50 °C to ± 5 °C, while the current fluctuation has also been reduced from ± 15 A to ± 2 A, allowing for precise control of the hardness of the material. This proves that the combination of Six Sigma and SAPF not only improves product quality but also optimizes the manufacturing process, reduces the cost of defective products, and improves overall efficiency.

The implementation of the Active Power Filter (SAPF) has proven to be effective in improving power quality, which in turn leads to improved product quality and production efficiency in the induction heating process. The main mechanism by which SAPF improves power quality lies in its ability to reduce harmonics, increase power factor, and stabilize input voltage, enabling the high-frequency heating system to operate with higher precision, reduce heating temperature fluctuations, and better control the hardness of the material. One of the major challenges of induction heating systems is the fluctuation of current and voltage, especially when high-order harmonics are present. Before SAPF was applied, the total harmonic distortion (THDi) was up to 36.35%, causing unstable current supply to the induction coil, changing the magnetic field density, and leading to uneven heat distribution in the material. When the sintering temperature fluctuates too much (± 50 °C), the hardness of the material becomes inconsistent, which significantly increases the rate of substandard products (90% defective before improvement). After SAPF was implemented, THDi was reduced to 5.21%, which makes the current supplied to the coil more stable, ensures uniform magnetic field density, and better controls the sintering temperature within ± 5 °C, thereby helping the material achieve optimal, more uniform hardness and reduces the product defect rate to only 1%. In addition, increasing the power factor (Cos Φ) from 0.89 to 0.98 also plays an important role in optimizing energy efficiency and reducing electricity loss. When the power factor is low, the large reactive energy leads to low efficiency of the induction coil, causing unnecessary heat loss and reducing heating efficiency. After SAPF improved the power factor, all the energy supplied to the induction coil was converted into heat more efficiently, making the system operate at a higher efficiency while reducing the power consumption from 55 kW to 48 kW, saving 12.73% of energy. This helps the enterprise save on electricity costs and extends the life of the high-frequency heating equipment by reducing unnecessary heat loads. Another important factor that SAPF directly affects product quality is the stability of the input voltage. Before the improvement, the voltage supplied to the high-frequency heating system fluctuated significantly due to the impact of nonlinear loads, reducing temperature control accuracy. When the voltage fluctuated suddenly, the current flowing through the induction coil was also affected, causing overheating or underheating at some stages of the treatment process. This reduced the heat treatment quality and led to undesirable changes in the material’s microstructure. After the integration of SAPF, the input voltage is stabilized, which allows for more precise control of the heating and cooling process, thereby ensuring the optimal phase transformation ratio from austenite to martensite, resulting in uniform hardness, better load-bearing capacity, and improved service life. The impact of SAPF is not only limited to improving product quality but also positively affecting the working environment and operator performance. When the system operates more stably, the number of machine stops due to technical errors is significantly reduced, allowing operators to intervene less in the system and reducing workload and stress levels during equipment monitoring. This was demonstrated by the employee satisfaction survey results, in which the perceived stability of the production process increased from 3.1 to 4.8, and the stress reduction increased by 46.67%. These factors help improve work efficiency, ensure smoother production processes, reduce human errors, and reduce equipment maintenance costs. Overall, SAPF brings comprehensive improvements in power quality, heat treatment performance, and product quality, as well as working environment and operating costs. Improvements in current and voltage control help optimize the heating process, reduce product defects, save energy, and improve the overall stability of the production system. This shows that incorporating SAPF into induction heat treatment systems has not only technical benefits but also brings significant economic value and labor efficiency, opening up wider application potential in industries requiring precise temperature control and efficient energy consumption.

The implementation of the Active Power Filter (SAPF) not only improves power quality but also brings significant economic benefits, helping businesses save operating costs and improve production efficiency. Before the SAPF was implemented, the annual energy cost of the high-frequency heating system was up to 120,000 USD, mainly due to power losses from harmonics and low power factor. After the SAPF was implemented, the power consumption was significantly reduced, reducing this cost to 96,000 USD, equivalent to a 20% savings due to the improvement of the power factor from 0.89 to 0.98 and the reduction of energy loss. In addition, the cost of production downtime (downtime losses) also changed significantly. Before the SAPF was integrated, voltage instability and temperature fluctuations in the high-frequency heating system caused a loss of about 35,000 USD per year due to equipment downtime for troubleshooting. Thanks to SAPF maintaining a stable current and minimizing interruptions in the heat treatment process, this loss was reduced to only $10,000, a reduction of 71.4%, allowing the system to operate more continuously and significantly reducing equipment downtime. At the same time, reducing harmonics and ensuring voltage stability also directly impacts product quality, helping to reduce the rate of defective products significantly. Before implementing SAPF, the rate of substandard products was up to 90%, causing a loss of about $50,000 per year due to having to discard or reprocess substandard products. After applying SAPF, the defect rate dropped to only 1%, helping the business save 90% of the costs related to manufacturing defects, reducing the loss to only $5,000. Thanks to the optimization of energy costs, reduction of product errors, and limited production interruptions, the total cost savings that SAPF brings are 45.9%, helping to improve the financial efficiency of the enterprise significantly. Notably, with this level of savings, the investment recovery period (ROI) for the SAPF system is calculated in just 18 months, showing the high feasibility and long-term benefits of applying SAPF in the industrial production environment. In general, SAPF not only helps to optimize operating performance but also brings clear economic benefits, helping enterprises reduce operating costs, increase profits, and improve competitiveness in the market. With a fast payback period and significant savings, SAPF becomes a sustainable and cost-effective solution for power quality management and production process optimization.

Research on the SAPF + Six Sigma model is of great significance in improving power quality and optimizing production, especially in the precision mechanical processing and high-frequency heat treatment industries. Controlling total harmonic distortion (THD) below 5% according to IEEE 519:2022 standard helps the induction heating process to be more stable, reducing the scrap rate from 90% to only 1%, optimizing production costs, and improving product quality. Applying 4.0 technology and RFID systems in real-time monitoring helps businesses proactively adjust operating parameters, reduce material loss, and improve production efficiency. This model has the potential to expand to many other industries, such as electronics, automobiles, aviation, and renewable energy, and is especially useful for small and medium enterprises (SMEs) that want to access high technology at a reasonable cost. In the future, the integration of artificial intelligence (AI), IoT, and big data analytics will help automate monitoring, optimize processes, and detect production problems early. In addition, this study not only focuses on optimizing technology but also applies data-driven quality management methods, helping businesses make more accurate decisions. In particular, assessing the satisfaction level of operating staff through PLS-SEM helps measure system efficiency in both technical and human aspects. In general, the SAPF + Six Sigma model improves product quality and helps businesses reduce costs, increase operational efficiency, and expand application capabilities to many different fields.

Several limitations affect the generalizability of the results. First, the SAPF + Six Sigma model was only tested on a specific manufacturing process and has not been widely validated in other industries, limiting its applicability. Second, the study has not integrated AI and IoT to optimize power quality in real-time, while this is an important trend in Industry 4.0. Third, the long-term impact of SAPF on equipment life and maintenance costs has not been assessed, making it difficult to determine its sustainable effectiveness. In addition, many small and medium-sized enterprises (SMEs) face financial and technical difficulties in implementing new technologies, reducing the feasibility of the model. Furthermore, the study used a PLS-SEM model, which has limitations in sample size and distribution and may not fully reflect the overall picture of the industry. These limitations make the research results mainly relevant to specific industries, especially mechanical manufacturing with high-frequency induction quenching processes. To improve, the research needs to be expanded to other areas, integrate AI and IoT to optimize the system and conduct long-term evaluations of the operational performance and economic benefits of SAPF. These improvements will help improve practical applicability and ensure the model can be deployed more effectively in different manufacturing environments.

## Conclusion

This study proposes an innovative model by integrating the MADM (Multi-Attribute Decision Making) method into the Six Sigma (DMAIC) process, which helps identify and rank issues affecting product quality during the hardening heat treatment process. In particular, the Taguchi method is applied in the improvement stage to optimize processing conditions, minimize product defects, and ensure stable quality. In addition, the study also incorporates Industry 4.0 technology into the control stage, deploys a real-time measurement system, and uses RFID to connect to the production management system to improve accuracy and minimize errors during operation. Another breakthrough is improving power quality with the SAPF (Shunt Active Power Filter) device, which helps reduce harmonic distortion (THD), ensuring that the power source meets IEEE 519:2022 standards, thereby improving machine accuracy and reducing product defect rates. In addition, the study uses the PLS-SEM model to evaluate the level of operator satisfaction after the system is improved, helping to identify factors affecting operational efficiency. Furthermore, the study also builds a model to calculate production costs and evaluate economic efficiency, showing that this improvement helps reduce the error rate from 90% to only 1%, significantly saving raw material and energy costs, thereby improving production efficiency. Thanks to these comprehensive improvements, the research model can be expanded and applied to many other industries, especially small and medium-sized manufacturing enterprises, helping to optimize costs, improve productivity, and ensure product quality.

Based on predetermined criteria, the fuzzy MADM technique assists practitioners with continuous improvement in prioritizing improvement concerns. Managers can easily choose issues from those that require selection for improvement by using the direct problem selection outcomes of the TOPSIS fuzzy method. The fuzzy MADM technique chooses the cost of energy resources as the primary cost reduction improvement item. Using Internet of Things (IoT) devices like RFID and barcode readers to execute planned control of self-managed maintenance and preventative maintenance for coils, the Six Sigma technique innovates based on Industry 4.0 technology. Relying on the induction heat treatment operator is eliminated when using the digital numerical control technology, which automatically calls the induction heat treatment program. Process operations are made simpler using Industry 4.0 technologies to run the induction heat treatment production process. Reduce production time and improve operations. Boost the pleasure of machine operators. The Industry 4.0 system’s warning system makes system maintenance easier. The machining program is called a system of the induction heat treatment machine, which uses digital numerical control, streamlines operations, and establishes a data connection between production processes and machining machines to build big data.

IoT devices are used in Industry 4.0 technology, therefore system operation requires understanding. This restricts the requirements for hiring operators in Vietnam. Even though Vietnamese people’s educational attainment has been increasing, many people in highland areas still lack a high school diploma, which limits their employment options. Nevertheless, implementing Industry 4.0 technology in a manufacturing setting is expected to be a future trend due to the great application efficiency of this technology.

You may want to think about taking the following actions to guarantee a steady power supply for induction heat treatment: Power supply capacity: Make sure the power supply can suit your induction heating equipment’s needs. To find out the power supply capacity required for your particular application, refer to the specifications provided by the equipment manufacturer. A sufficient power supply capacity will stop problems like overloading and voltage dips. Control of voltage: To maintain a stable voltage level, install voltage control devices like voltage regulators or stabilizers. These gadgets are capable of adjusting for variations in the input voltage and giving the induction heating apparatus a steady voltage supply. Power factor correction: Installing power factor correction capacitors will increase the power factor of your induction heating system. Power factor adjustment lowers reactive power demand and optimizes power utilization, which boosts system performance and lowers energy expenses. Use harmonic filters to reduce the harmonic distortion that the induction heating system is causing. By lowering harmonic currents and voltage distortion, harmonic filters contribute to a cleaner power source and less interference with other electrical equipment. Electrical grounding: Keeping a steady power supply requires proper electrical grounding. To reduce electrical noise, increase safety, and offer a steady reference potential, ensure the electrical system, including the induction heating equipment, is properly grounded. Frequent upkeep: Plan routine maintenance for the power supply system to detect and fix any problems quickly. Examine and tidy electrical connections, keep an eye on voltage levels, and look for indications of deterioration or wear in the power supply’s components. By being proactive, you can assist in averting possible issues and guaranteeing the power supply’s long-term dependability. A backup power source in the event of power fluctuations or outages, think about investing in an uninterruptible power supply (UPS) or backup power supply. These backup systems can temporarily supply power to shield delicate equipment and avoid process disturbances. A competent electrical engineer or power supply specialist should be consulted to determine your unique needs and put the right safeguards in place to guarantee a steady power source for your induction heat treatment procedure.

The Six Sigma method, used by most manufacturing businesses for continuous improvement initiatives, is the foundation for the continuous improvement model that this research article completes. This study differs from others in that it applies both traditional techniques, like statistical charts and Taguchi optimization method, and contemporary techniques, like MDAM and DNC method, based on Industry 4.0 technology application. The devices use IOT technology in the Analysis phase and Improvement phase of the Six Sigma method. This integrated analysis method is easy to use and meets high accuracy requirements because it makes use of commercial software, Minitab 18.0, and straightforward C# programming. The operator gathers data from the real production process, installs commercial software on a PC during the production process, analyzes the data, and independently confirms the findings at the source. However, because the production condition data in the process is so complex, professionals must also help operators analyze findings. This study’s continuous improvement model is thought to be novel and straightforward, suitable for use by all manufacturing firms in their ongoing efforts to enhance production stability, boost output, enhance product quality, and enhance overall business productivity. To help decision-makers more clearly identify the actual results of improvement activities and make better decisions to improve operations again in subsequent improvements, the author uses the PLS-SEM method to measure people’s satisfaction with the results of improvement activities according to measurement variables of technical readiness, information system technology, usefulness, and utility of improvement activities. This is considered a new action in this research model.

This study demonstrated that the Active Power Filter (SAPF) has a significant impact on the power, power quality, and product quality during induction heating. Before the implementation of SAPF, the high-frequency heating system encountered a high total harmonic distortion (THDi) of 36.35%, which caused instability in the current supplied to the induction coil, resulting in temperature fluctuations of up to ± 50 °C and reduced heat treatment uniformity. After the SAPF was integrated, the THDi was reduced to 5.21%, stabilizing the current and maintaining the heating temperature within the control range of ± 5 °C, thereby improving material uniformity and the hardness of the processed product. In addition, SAPF helps increase the power factor from 0.89 to 0.98, optimizing energy efficiency, reducing unnecessary electricity consumption, and reducing power consumption from 55 kW to 48 kW, equivalent to 12.73% energy savings. This not only helps businesses cut electricity operating costs but also extends the life of high-frequency heating equipment by reducing unnecessary heat loads. In addition to the impact on energy efficiency, SAPF also has a great impact on product quality. Before applying SAPF, the defective product rate reached 90%, mainly due to uneven heat distribution and unwanted fluctuations during heat treatment. After SAPF was implemented, the defective rate dropped to only 1%, equivalent to a 98.89% improvement, helping businesses reduce material waste, reduce remanufacturing costs, and improve overall product quality. These results show that SAPF helps improve the accuracy of heating temperature control and product quality but also helps optimize operating costs, reduce energy consumption, and increase production productivity. In particular, the return on investment (ROI) period is only 18 months, proving that the application of SAPF not only brings technical benefits but also has high economic feasibility. Overall, this study confirms that SAPF is an effective solution to improve power quality, optimize power consumption, and improve induction heat treatment performance, opening up the potential for wider application in industries requiring precise temperature control and energy saving.

This study proposed a model integrating Six Sigma with Active Power Filter (SAPF) to improve power quality and optimize the induction heating process in industrial production. Experimental results showed that the total harmonic distortion (THDi) was significantly reduced, from 36.35% to less than 5.21%, ensuring compliance with the IEEE 519:2022 standard. At the same time, the model helped improve product quality by controlling the hardening depth of the material well, reducing the defective product rate from 9.8 to 1.1%. Through the application of sensitivity analysis, the study identified the most important factors affecting power quality and heat treatment performance, including current intensity, heating time, and coil spacing. These findings are not only meaningful in improving the manufacturing process but also contribute significantly to the development of intelligent control systems for Industry 4.0. One of the important contributions of this study is the application of PLS-SEM to evaluate the satisfaction level of operating staff after implementing the improved system. The analysis results show a positive correlation between power quality, operating efficiency, and employee satisfaction, indicating the practical effectiveness of the model on both product quality and the working environment. In addition, the study also conducted a cost-benefit analysis, showing that the SAPF + Six Sigma model saves 45.9% of operating costs by reducing energy loss and improving machine efficiency. This not only helps manufacturing enterprises improve economic efficiency but also helps them comply with increasingly strict energy standards. However, the study still has certain limitations. First, the model has only been verified on a specific type of production process and has not been widely tested in many other industries. Expanding the application’s scope could help comprehensively evaluate the model’s generality. Second, while SAPF has proven effective in reducing harmonics, the model has not been integrated with artificial intelligence (AI) or Internet of Things (IoT) systems to optimize real-time power control. In addition, the study has not considered the long-term impact of SAPF application on equipment life and system maintenance costs over many years of operation. Future research directions could focus on extending the SAPF + Six Sigma model to other industries, such as electronic component manufacturing, automation, and renewable energy, to evaluate the system’s effectiveness under different production conditions. In addition, integrating AI and IoT to develop an intelligent control system could help the model operate more effectively in a volatile production environment, optimizing power flow and ensuring real-time power quality. The application of machine learning algorithms to predict current fluctuations in advance can also be an important approach to further improve the performance and stability of the system. In addition, research should also focus on developing optimal maintenance strategies to extend equipment life and reduce long-term operating costs. Overall, this study has provided a feasible and effective solution to improve power quality and production processes and opened up many important research directions for the future. The SAPF + Six Sigma model not only helps optimize production operations but also lays a solid foundation for the development of intelligent energy management systems in the Industry 4.0 era.

To ensure improved heat quality at tall quenching machines, further research on temperature management and control will be done in the future. This research will focus on automatic temperature control and installing temperature sensor systems and microcontrollers. Control to monitor and adjust the temperature while at work. Research on cooling system optimization can help improve thermal performance and quality at high-rise quenching machines, ensuring effective ventilation and maximizing energy usage. Examine and apply state-of-the-art insulating materials to the thermal system of the high-rise quenching machines. Track and assess heat quality data at high-rise quenching machines using artificial intelligence and information technology to forecast and recommend changes. APF shunt should be used and investigated to improve power quality. SAPF requires exact design and configuration in addition to in-depth electrical knowledge to ensure efficacy and safety.

**Fig. 32:**
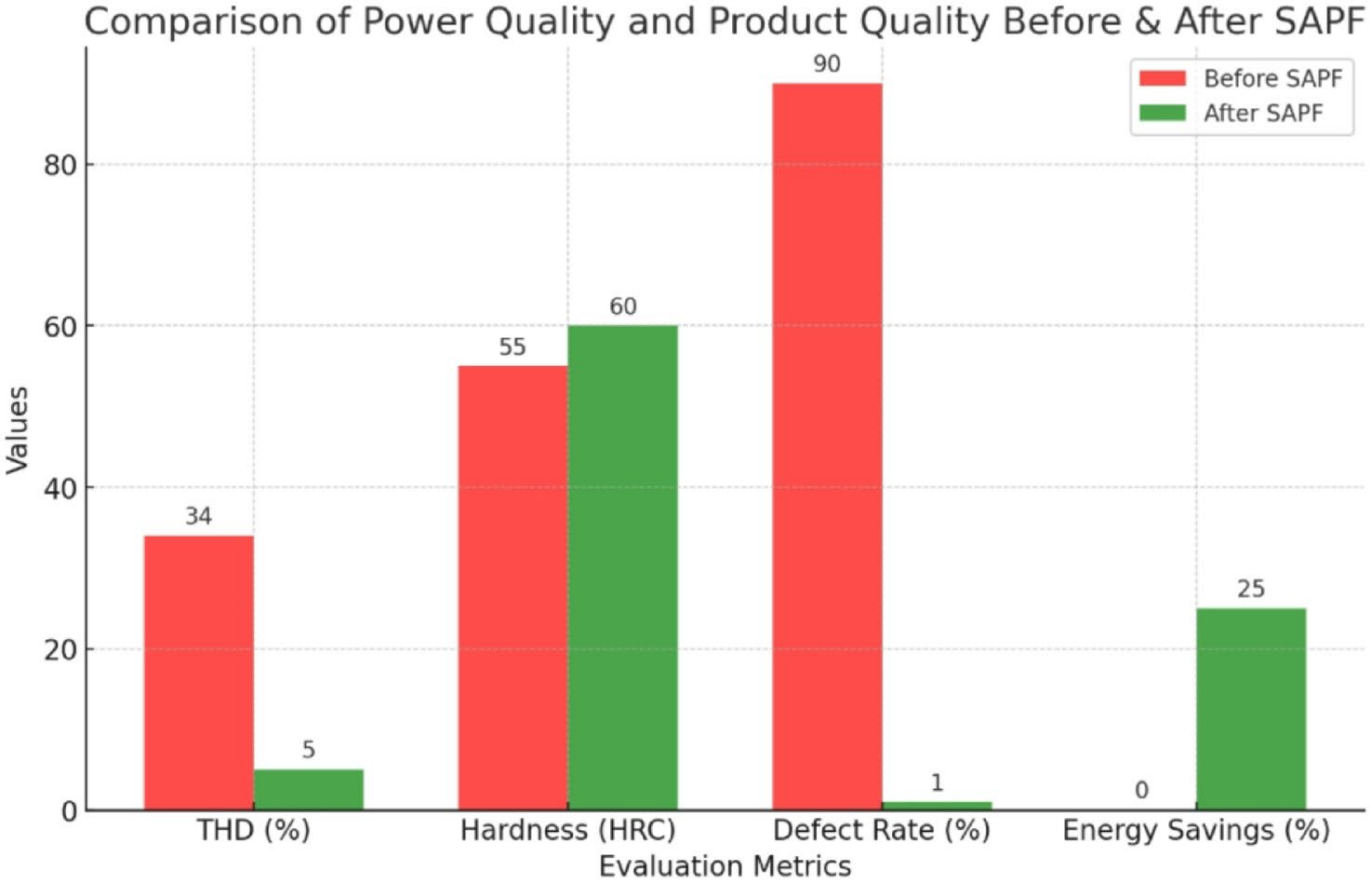
Comparison chart of power quality and product quality before and after SAPF implementation.

The power and product quality comparison chart before and after SAPF implementation shows significant improvements. Total harmonic distortion (THD (%)) decreased from 34 to 5%, helping to stabilize the power supply and improve the system’s operating efficiency. Product hardness (HRC) increased from 55 HRC to 60 HRC, ensuring better quality and meeting production standards. At the same time, the defect rate (Defect Rate (%)) decreased sharply from 90% to only 1%, demonstrating a clear improvement in production defect control. In addition, the energy savings (Energy Savings (%)) reached 25%, helping to optimize operating costs and improve electricity efficiency. These results confirm that applying SAPF improves power quality and improves product quality, reduces waste, and optimizes industry production efficiency (Fig. 32).

From the key findings of this study, manufacturing and trading companies can apply Active Power Filters (SAPFs) to improve power quality, increase production efficiency, and reduce operating costs. Integrating SAPFs into existing production systems will help reduce harmonics, improve power factor, and stabilize voltage, especially in processes that require high stability, such as heat treatment, die casting, and metal processing, thereby improving equipment efficiency and product quality. For plants with nonlinear loads, SAPFs help minimize the impact of harmonics on inverter motors, induction furnaces, and electric drive systems, limiting production interruptions, which is especially useful in continuous manufacturing industries such as automobile manufacturing, electronics, and chemicals. In addition, to optimize the effectiveness of SAPF, businesses need to strengthen training for operators, helping them understand how to use and maintain the system, thereby minimizing technical risks, extending equipment life, and improving labor efficiency. Another important strategy is to integrate SAPF into sustainable development goals, helping businesses reduce energy consumption and carbon emissions, meet environmental standards such as ISO 50,001, and enhance reputation and competitiveness in the international market. In addition, businesses can combine SAPF with IoT and AI technology to build a real-time power quality monitoring system, helping to predict and prevent incidents, optimize energy efficiency, and improve production productivity. Before deploying SAPF, businesses should also build a financial performance evaluation model, using cost savings and payback period data from this study to support the investment decision-making process, ensuring economic feasibility. In particular, SAPF is not only limited to the metallurgy and metal processing industry but can also be extended to other fields such as electronic component manufacturing, food processing, and renewable energy, where high stability in power quality is required to ensure continuous operation of electric drive systems and precision machinery. In general, manufacturing and trading companies can take advantage of the results of this research to optimize production processes, reduce waste, increase profits, and contribute to sustainable development. The implementation of SAPF not only brings short-term benefits in terms of cost savings and increased productivity but also helps businesses improve their competitiveness in the global market.

## Data Availability

Correspondence and requests for materials should be addressed to M.L.D.

## References

[1] Dalenogare, L. S., Benitez, G. B., Ayala, N. F. & Frank, A. G. The expected contribution of industry 4.0 technologies for industrial performance. *Int. J. Prod. Econ.* **204**, 384–394. 10.1016/j.ijpe.2018.08.019 (2018).

[2] Zhang, M., Zhang, Y., Liu, Y. & Zhang, Y. The evolution of production scheduling from industry 3.0 through industry 4.0. *Int. J. Prod. Res.* **59** (16), 4960–4979. 10.1080/00207543.2021.1925772 (2021).

[3] Zakoldaev, D. A., Korobeynikov, A. G., Shukalov, A. V., Zharinov, I. O. & Zharinov, O. O. Industry 4.0 vs Industry 3.0: the role of personnel in production, *IOP Conference Series: Materials Science and Engineering*, 734, 012048, (2020). 10.1088/1757-899x/734/1/012048

[4] Rosin, F., Forget, P., Lamouri, S. & Pellerin, R. Enhancing the Decision-Making process through industry 4.0 technologies. *Sustainability* **14** (461), 1–35. 10.3390/su14010461 (2022).

[5] Dalmarco, G., Ramalho, F. R., Barros, A. C. & Soares, A. L. Providing industry 4.0 technologies: the case of a production technology cluster. *J. High. Technol. Manage. Res.* **100355**, 1–9. 10.1016/j.hitech.2019.100355 (2019).

[6] Tong, D., Gu, J. & Yang, F. Numerical simulation on induction heat treatment process of a shaft part: involving induction hardening and tempering. *J. Mater. Process. Technol.* **262**, 277–289. 10.1016/j.jmatprotec.2018.06.043 (2018).

[7] Rojas-Arias, N., Coury, F. G., Vanmeensel, K., Amancio-Filho, S. T. & Gargarella, P. Heat treating additive-manufactured alloys: A comprehensive review. *J. Alloys Compd.* **1005**, 176035. 10.1016/j.jallcom.2024.176035 (2024).

[8] Fomin, A. Superhard Titania coatings produced on titanium using induction heat treatment. *Ceram. Int.* **45**, 8258–8264. 10.1016/j.ceramint.2019.01.131 (2019).

[9] Cheng, G. J., Liu, L. T., Qiang, X. J. & Liu, Y. Industry 4.0 Development and Application of Intelligent Manufacturing, *International Conference on Information System and Artificial Intelligence (ISAI)*, Hong Kong, China, 407–410, (2016) 10.1109/ISAI.2016.0092

[10] Dantas, T. E. T. et al. How the combination of circular economy and industry 4.0 can contribute towards achieving the sustainable development goals. *Sustainable Prod. Consum.* **26**, 213–227. 10.1016/j.spc.2020.10.005 (2021).

[11] Rudnev, V. Evolution of Induction Heating and Heat Treating as a Subject of Mathematical Modeling, Optimization and Design, *XXI International Conference Complex Systems: Control and Modeling* Problems *(CSCMP)*, Samara, Russia (2019). 10.1109/CSCMP45713.2019.8976826

[12] Palange, A. & Dhatrak, P. Lean manufacturing a vital tool to enhance productivity in manufacturing. *Mater. Today: Proc.* **46**, 729–736. 10.1016/j.matpr.2020.12.193 (2021).

[13] Widiwati, I. T. B., Liman, S. D. & Nurprihatin, F. The implementation of lean six Sigma approach to minimize waste at a food manufacturing industry. *J. Eng. Res.* **12**, 1, 1–10. 10.1016/j.jer.2024.01.022 (2024).

[14] Psarommatis, F. & Azamfirei, V. Zero defect manufacturing: A complete guide for advanced and sustainable quality management. *J. Manuf. Syst.* **77**, 764–779. 10.1016/j.jmsy.2024.10.022 (2024).

[15] LeBlanc, S., Yee, S. K., Scullin, M. L., Dames, C. & Goodson, K. E. Material and manufacturing cost considerations for thermoelectrics. *Renew. Sustain. Energy Rev.* **32**, 313–327. 10.1016/j.rser.2013.12.030 (2015).

[16] Nakandala, D., Rashid, M. A. & de Silva, P. G. T. The role of lean, agility and learning ambidexterity in industry 4.0 implementations. *Technol. Forecast. Soc. Chang.* **202**, 122204. 10.1016/j.techfore.2024.122204 (2024).

[17] Kuosmanen và, N. & Maczulskij, T. Going green while getting Lean: decomposing carbon and green total factor productivity. *J. Environ. Manage.* **352, 120046**, 10.1016/j.jenvman.2024.120046 (2024). 10.1016/j.jenvman.2024.120046 38194869

[18] Barrani, L., Ostadi, B. & Aghdasi, M. Developing a strategic roadmap towards integration in industry 4.0: A dynamic capabilities theory perspective. *Technol. Forecast. Soc. Chang.* **202** (122204). 10.1016/j.techfore.2024.122204 (2024).

[19] Pech, M. & Vrchota, J. Classification of Small- and Medium-Sized enterprises based on the level of industry 4.0 implementation. *Appl. Sci.* **10** (15), 1–22. 10.3390/app10155150 (2020). 5150.

[20] Agarwal, A. & Ojha, R. Prioritizing implications of Industry-4.0 on the sustainable development goals: A perspective from the analytic hierarchy process in manufacturing operations. *J. Clean. Prod.* **444**, 141189. 10.1016/j.jclepro.2024.141189 (2024).

[21] Pongboonchai-Empl, T., Antony, J., Garza-Reyes, J. A., Komkowski, T. & Tortorella, G. L. Integration of Industry 4.0 technologies into Lean Six Sigma DMAIC: A systematic review, *Production Planning & Control*, (2023). 10.1080/09537287.2023.2188496

[22] Li, G., Reimann, M. & Zhang, W. When remanufacturing Meets product quality improvement: the impact of production cost. *Eur. J. Oper. Res.* **271** (3), 913–925. 10.1016/j.ejor.2018.05.060 (2018).

[23] Vinogradova, I. Multi-Attribute Decision-Making methods as a part of mathematical optimization. *Mathematics* **7** (10), 915. 10.3390/math7100915 (2019).

[24] Kousar, S., Ansar, A., Kausar, N. & Freen, G. Multi-Criteria Decision-Making for smog mitigation: A comprehensive analysis of health, economic, and ecological impacts. *Spectr. Decis. Mak. Appl.* **2** (1), 53–67. 10.31181/sdmap2120258 (2024).

[25] Ye, J., Zhan, J. & Xu, Z. A novel multi-attribute decision-making method based on fuzzy rough sets. *Comput. Ind. Eng.* **155**, 1–20. 10.1016/j.cie.2021.107136 (2021).

[26] Jiang, J., Ren, M. & Wang, J. Interval number multi-attribute decision-making method based on TOPSIS. *Alexandria Eng. J.* **61**, 5059–5064. 10.1016/j.aej.2021.09.031 (2021).

[27] Shahba, S., Arjmandi, R., Monavari, M. & Ghodusi, J. Application of multi-attribute decision-making methods in SWOT analysis of mine waste management (case study: Sirjan’sGolgohar iron mine, Iran). *Resour. Policy*. **51**, 67–76. 10.1016/j.resourpol.2016.11.002 (2017).

[28] Zhu, J., Ma, X., Zhan, J. & Yao, Y. A three-way multi-attribute decision making method based on regret theory and its application to medical data in fuzzy environments. *Appl. Soft Comput.* **123**, 1–28. 10.1016/j.asoc.2022.108975 (2022).

[29] Dabić-Miletić, S. & Raković, K. Ranking of autonomous alternatives for the realization of intralogistics activities in sustainable warehouse systems using the TOPSIS method. *Spectr. Eng. Manage. Sci.* **1** (1), 48–57. 10.31181/sems1120234m (2023).

[30] Duc, M. L., Bilik, P. & Martinek, R. Analysis of factors affecting electric power quality: PLS-SEM and deep learning neural network analysis. *IEEE Access.* **11**, 40591–40607. 10.1109/ACCESS.2023.3268037 (2023).

[31] Sharma, M., Joshi, S., Luthra, S. & Kumar, A. Impact of digital assistant attributes on millennials’ purchasing intentions: A Multi-Group analysis using PLS-SEM, artificial neural network and FsQCA. *Inform. Syst. Front.* **26** (3), 943–966. 10.1007/s10796-022-10339-5 (2024). 10.1007/s10796-022-10339-5 PMC9510515 36185777

[32] Yumak, N. & Aslantaş, K. A review on heat treatment efficiency in metastable Β titanium alloys: the role of the treatment process and parameters. *J. Mater. Res. Technol.* **9** (6), 15360–15380. 10.1016/j.jmrt.2020.10.088 (2020).

[33] Khan, O. et al. Experimental investigation and multi-performance optimization of the leachate recirculation-based sustainable landfills using the Taguchi approach and an integrated MCDM method. *Sci. Rep.* **13**, 1–20. 10.1038/s41598-023-45885-8 (2023). 37925554 10.1038/s41598-023-45885-8 PMC10625540

[34] Minamoto, H. & Kawamura, S. Effects of material strain rate sensitivity in low-speed impact between two identical spheres. *Int. J. Impact Eng.* **36** (5), 680–686. 10.1016/j.ijimpeng.2008.10.001 (2009).

[35] Li, R., Yamashita, S., Yoshida, K. & Kita, H. Effect of counterbody on friction and wear properties of copper-MgP-graphite composites prepared by powder metallurgy, *Processes*, 10, 804, 1–12, (2022). 10.3390/pr10050804

[36] ELLIOTT, A. Heat Treatment of sodium chloride windows to increase resistance to atmospheric action, Nature 157, 299, 1–2 2, (1946). 10.1038/157299a0

[37] El-Bagoury, N., Waly, M. & Nofal, A. Effect of various heat treatment conditions on the microstructure of cast polycrystalline IN738LC alloy. *Materials Sci. Engineering: A*. **487** (1–2), 152–161. 10.1016/j.msea.2007.10.004 (2008).

[38] Xiang, C., Gupta, N., Coelho, P. & Cho, K. Effect of microstructure on tensile and compressive behavior of WE43 alloy in as cast and heat treated conditions. *Materials Sci. Engineering: A*. **710**, 74–85. 10.1016/j.msea.2017.10.084 (2018).

[39] Naar, R. & Bay, F. Numerical optimization for induction heat treatment processes. *Appl. Math. Model.* **37** (4), 2074–2085. 10.1016/j.apm.2012.04.058 (2013).

[40] Magnabosco, I., Ferro, P., Tiziani, A. & Bonollo, F. Induction heat treatment of an ISO C45 steel bar: experimental and numerical analysis. *Comput. Mater. Sci.* **35** (2), 98–106. 10.1016/j.commatsci.2005.03.010 (2006).

[41] Zakeri, S. et al. A decision analysis model for material selection using simple ranking process. *Sci. Rep.* **13**, 1–19. 10.1038/s41598-023-35405-z (2023). 8631. 37244904 10.1038/s41598-023-35405-z PMC10224978

[42] Fomin, A. et al. Simulation and experimental study of induction heat treatment of titanium disks. *Int. J. Heat Mass Transf.* **165**, 120668–120661. 10.1016/j.ijheatmasstransfer.2020.120668 (2021).

[43] Kunst, R. et al. Improving devices communication in industry 4.0 wireless networks. *Eng. Appl. Artif. Intell.* **83**, 1–12. 10.1016/j.engappai.2019.04.014 (2019).

[44] Li, L. et al. Exogenous melatonin improves peanut field productivity and quality at reduced nitrogen application. *Field Crops Res.* **319**, 109650. 10.1016/j.fcr.2024.109650 (2024).

[45] Khanduja, D., Singh, S. & Garg, R. K. Analysis of critical success factors for successful integration of lean six Sigma and industry 4.0 for organizational excellence. *TQM J.* **36**, 1 123–140. 10.1108/TQM-07-2022-0215 (2023).

[46] Ferencez, K., Domokos, J. & Kovacs, L. Review of Industry 4.0 Security Challenges, *2021 IEEE 15th International Symposium on Applied Computational Intelligence and Informatics (SACI)*, Timisoara, Romania, 245–248, (2021). 10.1109/SACI51354.2021.9465613

[47] Harikannan, N., Vinodh, S. & Antony, J. Analysis of the relationship among industry 4.0 technologies, sustainable manufacturing practices and organizational sustainable performance using structural equation modelling. *TQM J.* **35**, 1. 42–72. 10.1108/TQM-02-2023-0044 (2023).

[48] Zhong, Y., Oh, S. & Moon, H. Service transformation under industry 4.0: investigating acceptance of facial recognition payment through an extended technology acceptance model. *Technol. Soc.* **64**, 101515, 1–10. 10.1016/j.techsoc.2020.101515 (2021).

[49] Rikalovic, A., Suzic, N., Bajic, B. & Piuri, V. Industry 4.0 implementation challenges and opportunities: A technological perspective. *IEEE Syst. J.* **16**, 2, 2797–2810. 10.1109/JSYST.2021.3101673 (2022).

[50] Unhelkar, B. et al. Enhancing supply chain performance using RFID technology and decision support systems in the industry 4.0–A systematic literature review. *International J. Inform. Manage. Data Insights*, **2**, 100084, 1–12, 10.1016/j.jjimei.2022.100084

[51] Zhang, J. et al. Robust RFID based 6-DoF localization for unmanned aerial vehicles. *IEEE Access.* **7**, 77348–77361. 10.1109/ACCESS.2019.2922211 (2019).

[52] Panagoulias, D. P., Virvou, M. & Tsihrintzis, G. A. A novel framework for artificial intelligence explainability via the technology acceptance model and rapid estimate of adult literacy in medicine using machine learning. *Expert Syst. Appl.* **248**, 123375. 10.1016/j.eswa.2024.123375 (2024).

[53] Grassi, A., Guizzi, G., Carmela, L., Santillo & Vespoli, S. A Semi-Heterarchical Production Control Architecture for Industry 4.0-based manufacturing systems, *Manufacturing Letters* **24** 43–46. (2020). 10.1016/j.mfglet.2020.03.007

